# Targeting the angiopoietin-like protein 3/8 complex with a monoclonal antibody in patients with mixed hyperlipidemia: a phase 1 trial

**DOI:** 10.1038/s41591-025-03830-4

**Published:** 2025-07-10

**Authors:** Daniel Gaudet, Malgorzata Gonciarz, Xi Shen, Jennifer K. Leohr, Thomas P. Beyer, Jonathan W. Day, Garrett R. Mullins, Eugene Y. Zhen, Maryalice Hartley, Miriam Larouche, Robert J. Konrad, Olivier Benichou, Giacomo Ruotolo

**Affiliations:** 1 Department of Medicine, Université de Montréal and ECOGENE-21, Saguenay, Quebec Canada; 2 Lilly Research Laboratories, Eli Lilly and Company, Indianapolis, IN USA

**Keywords:** Metabolic disorders, Outcomes research

## Abstract

The angiopoietin-like protein 3/8 complex (ANGPTL3/8) inhibits lipoprotein lipase (LPL) activity, primarily in oxidative tissues, and does so more potently than ANGPTL3, making ANPTL3/8 an attractive target for treating dyslipidemia. This study enrolled 48 adults (36 men, 12 women) with mixed hyperlipidemia to assess the primary outcome of safety and the secondary outcomes of pharmacokinetics and pharmacodynamics of ascending doses of LY3475766, a human monoclonal antibody that specifically blocks ANGPTL3/8-mediated inhibition of LPL activity. Participants received a single dose of LY3475766 or placebo. LY3475766 was well tolerated with no severe adverse events or adverse event-related discontinuations. Compared with placebo, LY3475766 dose-dependently reduced the concentration of triglycerides (−70%), remnant cholesterol (−86%), low-density lipoprotein cholesterol (−32%), non-high-density lipoprotein cholesterol (non-HDL-C) (−35%) and apolipoprotein B (−29%) while increasing HDL-C (+27%). LY3475766 thus significantly reduced atherogenic lipoprotein levels while increasing HDL-C levels; however, the effects on cardiovascular risk remain to be established. ClinicalTrials.gov registration: NCT04052594.

## Abstract (web summary)

In a phase 1 trial, treatment of individuals with mixed hyperlipidemia with a monoclonal antibody targeting the angiopoietin-like protein 3/8 complex resulted in dose-dependent reductions in the levels of triglycerides and low-density lipoprotein cholesterol, while increasing the levels of high-density lipoprotein.

## Main

In recent years, agents inhibiting angiopoietin-like protein 3 (ANGPTL3) have been developed and have been shown to be effective in a wide spectrum of lipid disorders, including homozygous hypercholesterolemia^1–3^, refractory hypercholesterolemia^4^, mixed hyperlipidemia^5^ and severe hypertriglyceridemia with chylomicronemia provided that lipoprotein lipase (LPL) is bioavailable^6^. ANGPTL3 is part of the broad ANGPTL protein family that includes three main isoforms (ANGPTL3, ANGPTL4 and ANGPTL8) that play a pivotal role in LPL-dependent lipid metabolism and which operate in a coordinated and sophisticated manner according to prandial status^7,8^. ANGPTL3 is expressed mainly in the liver, ANGPTL8 is expressed mostly in the liver and the adipose tissue, and ANGPTL4 is expressed in the adipose tissue, liver, intestine and other tissues^7^. LPL is the key enzyme responsible for hydrolyzing triglycerides in triglyceride-rich lipoproteins to provide fatty acids for uptake into oxidative tissues, such as skeletal muscle for energy and adipose tissue for re-esterification and storage^9–12^. Recent studies on how the ANGPTL protein family modulates LPL activity have identified the mechanisms by which LPL activity is controlled in a tissue-specific manner^7,8^.

In the postprandial state, the insulin-responsive ANGPTL8 protein forms complexes with the LPL inhibitors ANGPTL3 and ANGPTL4 to differentially regulate their LPL-inhibitory activities^13,14^. It is thought that these ANGPTL3/8 and ANGPTL4/8 complexes could form intracellularly to then be released from hepatocytes and adipocytes as intact complexes. ANGPTL8 forms localized ANGPTL4/8 complexes in adipose tissue that act in an autocrine and paracrine manner to decrease the ability of ANGPTL4 to inhibit LPL and to preserve maximum LPL activity in the adipose tissue after feeding^15–18^. In contrast, when ANGPTL8 associates with ANGPTL3, the resulting ANGPTL3/8 complex that is secreted by the liver acts in an endocrine manner as a potent inhibitor of LPL activity in oxidative tissues and has a greater effect on LPL than ANGPTL3 alone^15,17,19^. The ability of ANGPTL3/8 to inhibit LPL activity has been shown to be suppressed by apolipoprotein (Apo)A-V, resulting in remnant cholesterol and triglyceride lowering^20–23^.

Naturally occurring *ANGPTL3* loss-of-function (LOF) variants are associated with lower levels of low-density lipoprotein cholesterol (LDL-C), triglycerides and high-density lipoprotein cholesterol (HDL-C), and a reduced risk of cardiovascular disease (CVD)^24–26^. Carriers of naturally occurring *ANGPTL8* LOF variants, which reduce formation of the ANGPTL3/8 complex, show decreased triglyceride and LDL-C levels, increased HDL-C levels, and a reduced risk of CVD^27^. In contrast, carriers of *APOA5* LOF mutations, which result in increased ANGPTL3/8 activity, demonstrate increased triglyceride levels and an increased risk of CVD^28^.

Collectively, these observations suggest that ANGPTL3/8 may be an attractive drug target. Therefore, we developed a fully human anti-ANGPTL3/8 antibody, LY3475766, that binds to the same ANGPTL3/8 active site epitope that is recognized by LPL and ApoA-V^29^. To do this, we immunized AlivaMab mice (which produce fully human antibodies) with recombinant human ANGPTL3/8 complex. Antibodies resulting from this immunization were counter-screened to eliminate those that bound ANGPTL3 or ANGPTL8 alone. Afterward, ANGPTL3/8-specific antibodies were tested in LPL activity assays to identify those that potently neutralized the LPL-inhibitory activity of both human and mouse ANGPTL3/8. The remaining antibodies were then screened in a series of in vitro preclinical assays to exclude those with immunogenic risk.

The single remaining antibody (LY3475766) was confirmed to bind the ANGPTL3/8 active site where ApoA-V and LPL bind by using hydrogen–deuterium exchange mass spectrometry^29^. This antibody inhibited human ANGPTL3/8 more potently than evinacumab while having no evinacumab-like effect on ANGPTL3-mediated inhibition of LPL or endothelial lipase^29^. In preclinical studies, a single dose of LY3475766 substantially decreased triglycerides in mice with hypertriglyceridemia and showed durability^29^. In the current study, we describe a single ascending dose trial conducted with LY3475766 to assess its safety, tolerability, pharmacokinetics and pharmacodynamics in participants with mixed hyperlipidemia (NCT04052594).

## Results

### Participant disposition

**Extended Data Fig. 1:**
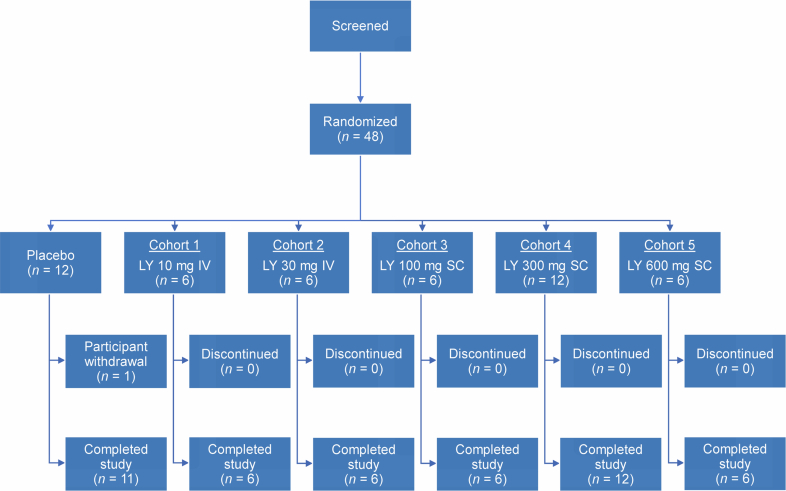
Participant disposition flow chart describing dosing strategy and discontinuations. Participants were randomized 6:2 to LY3475766 or placebo (0.9% sodium chloride) in each cohort. The 300 mg dose group included 12 participants treated with LY3475766 due to restarting during the COVID-19 pandemic. IV, intravenous; LY, LY3475766; n, number of participants; SC, subcutaneous.

All 48 participants who entered the study received one dose of study treatment, and 47 participants completed the study (Extended Data Fig. 1). Due to the COVID-19 pandemic, most of the first eight participants in the 300 mg cohort were unable to attend their outpatient visits on days 15–57. To collect sufficient data for the 300 mg LY3475766 dose, this cohort was restarted with eight new participants (who completed all visits). Because each of these groups included six participants treated with 300 mg LY3475766 and two placebo-treated participants, a total of 12 participants were given 300 mg LY3475766. The data for the 300 mg dose group represent all available data from participants dosed with 300 mg LY3475766.

### Baseline characteristics

Baseline characteristics are listed in Table 1. A total of 75% of the participants were male, with a median age of 49 years, body mass index of 30.4 kg m^−2^, triglycerides 194 mg dl^−1^ and LDL-C 143 mg dl^−1^. Treatment groups were comparable for most baseline demographics and for LDL-C, although modest imbalances were observed for median baseline triglyceride level across the groups.

**Table 1:** Baseline characteristics of healthy participants with dyslipidemia

	Pooled placebo (*n* = 12)	LY3475766				
		10 mg IV (*n* = 6)	30 mg IV (*n* = 6)	100 mg SC (*n* = 6)	300 mg SC (*n* = 12)	600 mg SC (*n* = 6)
Age (years)	52 (20, 65)	44 (25, 61)	50 (31, 62)	56 (30, 60)	52 (33, 65)	51 (31, 58)
Male sex (%)	75	100	100	50	67	67
BMI (kg m^−2^)	29.5 (25.1, 37.1)	33.4 (26.7, 36.4)	27.1 (21.5, 37.0)	29.3 (26.0, 37.5)	30.5 (24.9, 38.5)	31.4 (25.8, 36.5)
Triglycerides (mg dl^−1^)	194 (143, 331)	229 (156, 292)	143 (97, 213)	160 (128, 300)	196 (124, 429)	176 (159, 234)
LDL-C (mg dl^−1^)	142 (94, 197)	139 (85, 186)	134 (68, 179)	155 (98, 219)	138 (97, 263)	145 (102, 237)
HDL-C (mg dl^−1^)	41 (28, 64)	41 (37, 45)	38 (26, 57)	52 (42, 71)	46 (32, 64)	45 (35, 70)
Non-HDL-C (mg dl^−1^)	178 (116, 215)	181 (112, 219)	153 (87, 202)	175 (121, 278)	179 (130, 293)	164 (122, 265)
Remnant cholesterol (mg dl^−1^)	26 (18, 48)	30 (23, 54)	21 (10, 25)	24 (16, 59)	31 (25, 86)	22 (15, 36)
ApoA-I (mg dl^−1^)	119 (99, 154)	129 (106, 136)	92 (80, 151)	153 (124, 191)	146 (116, 190)	NA
ApoB (mg dl^−1^)	123 (93, 152)	119 (77, 149)	106 (63, 142)	111 (81, 169)	130 (97, 209)	116 (104, 188)
ApoC-III (mg dl^−1^)	13 (11, 20)	13 (12, 17)	10 (7, 14)	12 (8, 18)	14 (10, 29)	16 (11, 19)

Unless otherwise indicated, data are given as median (minimum, maximum). BMI, body mass index; IV, intravenous; NA, not available; SC, subcutaneous.

### Safety and tolerability

No deaths, serious adverse events (AEs), severe treatment-emergent AEs (TEAEs) or discontinuations due to TEAEs were observed. All TEAEs were mild or moderate. The proportion of participants with at least one TEAE was not higher in the LY3475766 groups than in the placebo group. The most common TEAEs related to study treatment were injection-site reactions (paresthesia and edema), headache and diarrhea. Three changes seen on electrocardiography were reported as TEAEs: a second-degree atrioventricular block (with placebo); an instance of ventricular extrasystoles (with placebo); and a single 7 second event of monomorphic ventricular tachycardia with 100 mg LY3475766 detected during planned telemetry. The frequency of all TEAEs in each group is shown in Extended Data Table 1. Of the 36 LY3475766-treated participants, no participant developed treatment-emergent antidrug antibodies.

### Pharmacokinetics and target engagement

**Fig. 1:**
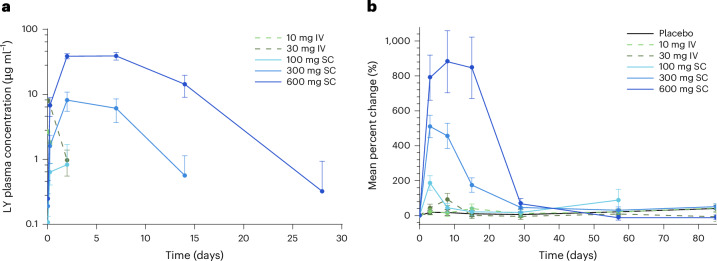
Pharmacokinetics of LY3475766 and mean percent change in ANGPTL3/8 complex concentration. **a**, LY3475766 (LY) plasma concentration over time (LY 10 mg, *n* = 6; LY 30 mg, *n* = 6; LY 100 mg, *n* = 6; LY 300 mg, *n* = 12; LY 600 mg, *n* = 6). **b**, Percent change from baseline in ANGPTL3/8 plasma concentration after either intravenous (IV; dashed lines) or subcutaneous (SC; solid lines) treatment with LY3475766 (placebo, *n* = 12; LY 10 mg, *n* = 6; LY 30 mg, *n* = 6; LY 100 mg, *n* = 6; LY 300 mg, *n* = 12; LY 600 mg, *n* = 6). Data are given as the least-squares mean (s.e.). *n*, number of participants. Source data

Following intravenous treatment, LY3475766 concentration was detectable up to 6 hours after dosing for the 10 mg group and up to 2 days after dosing for the 30 mg group (Fig. 1a). Following subcutaneous treatment, LY3475766 concentration increased with increasing dose level. Assessment of the dose proportionality for LY3475766 showed that the maximum observed drug concentration (*C*_max_) increased in a supra-proportional manner. The ratio of the dose-normalized geometric mean *C*_max_ of 600 mg versus 100 mg LY3475766 was 6.9 (90% confidence interval: 2.9–16.3). Assessment of the area under the operating curve of LY3475766 was not conducted given insufficient data. Following subcutaneous treatment, the time to LY3475766 *C*_max_ occurred later with increasing dose level: approximately 27 h for the 100 mg dose, 48 h for the 300 mg dose, and 108 h for the 600 mg dose. Given the limited data available, further analysis is needed for a reliable calculation of half-life.

**Extended Data Fig. 2:**
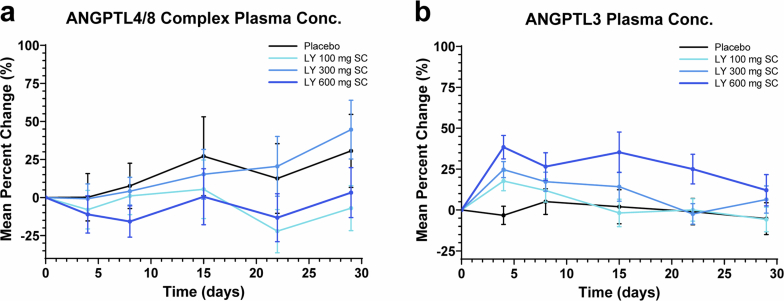
Changes in ANGPTL4/8 and ANGPTL3 concentrations in response to LY3475766 treatment. Percent changes from baseline over time following SC administration of LY3475766 are shown for (**a**) ANGPTL4/8 (placebo, n = 5; LY 100 mg, n = 6; LY 300 mg, n = 12; LY 600 mg, n = 6) and (**b**) ANGPTL3 (placebo, n = 4; LY 100 mg, n = 6; LY 300 mg, n = 10; LY 600 mg, n = 5). Data are expressed as least-squares mean (standard error). Conc., concentration; LY, LY3475766; n, number of participants; SC, subcutaneous. Source data

No change in serum ANGPTL3/8 concentration was detected in the 10 mg group, while modestly increased serum ANGPTL3/8 concentration was observed for the 30 mg and 100 mg groups (Fig. 1b). Substantially elevated ANGPTL3/8 was observed in the 300 mg and 600 mg dose groups from days 3 to 15, while a greater than 756% placebo-adjusted increase was observed in the 600 mg group from days 3 to 15 (Fig. 1b). ANGPTL3 and ANGPTL4/8 concentrations were also assessed in response to LY3475766 (Extended Data Fig. 2). The only change observed was a modest increase in ANGPTL3 level.

### Maximum percent change in lipid fractions

**Fig. 2:**
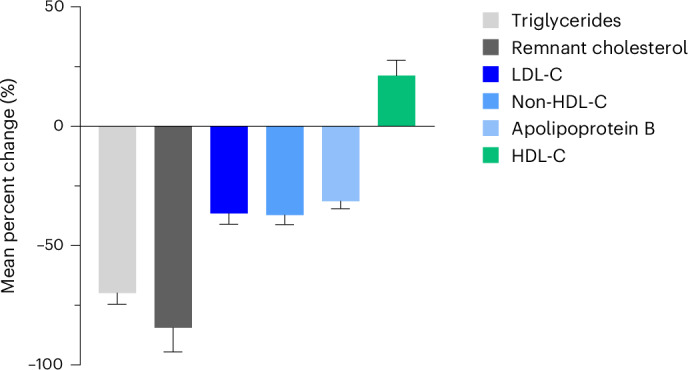
Maximum LY3475766-induced percent placebo-adjusted change in lipid fractions. The maximum triglyceride reduction observed was −70.4% (5.8%) (600 mg dose at day 8; *n* = 6). The maximum remnant cholesterol reduction observed was −85.7% (13.1%) (300 mg dose at day 15; *n* = 9). The maximum LDL-C reduction observed was −31.9% (6.3%) (600 mg dose at day 15; *n* = 6). The maximum non-HDL-C reduction observed was −34.9% (5.4%) (600 mg dose at day 15; *n* = 6). The maximum apolipoprotein B reduction observed was −29.3% (4.3%) (600 mg dose at day 15; *n* = 6). The maximum HDL-C increase observed was +26.7% (8.6%) (600 mg dose at day 15; *n* = 6). Data in the legend are given as the least-squares mean difference from baseline (s.e.). Data in the figure are given as the least-squares mean difference from placebo (s.e.). Source data

Figure 2 shows the maximum least-squares mean (s.e.) percent placebo-adjusted changes observed after treatment with LY3475766 for triglycerides (−69.9% (4.7%)), remnant cholesterol (−84.4% (10.2%)), LDL-C (−36.5% (4.6%)), non-HDL-C (−37.2% (4.1%)), ApoB (−31.4% (3.2%)) and HDL-C (+21.2% (6.5%)). These changes all occurred with the 600 mg dose at day 15 except for the maximum reductions in triglycerides (600 mg dose at day 8) and remnant cholesterol (300 mg dose at day 15).

### Effects on triglyceride-rich lipoproteins

**Fig. 3:**
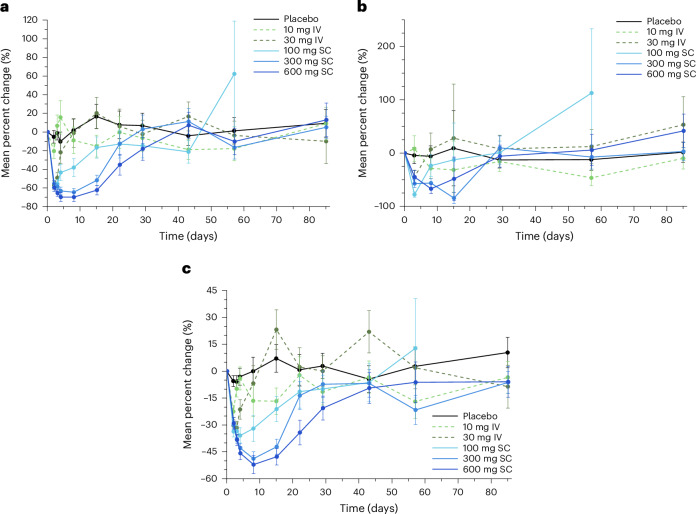
LY3475766-induced changes in triglyceride, remnant cholesterol and ApoC-III concentration. **a**–**c**, Percent change from baseline for triglycerides (**a**), remnant cholesterol (**b**) and ApoC-III (**c**) over time after either IV (dashed lines) or SC (solid lines) treatment with LY3475766 (placebo, *n* = 12; LY 10 mg, *n* = 6; LY 30 mg, *n* = 6; LY 100 mg, *n* = 6; LY 300 mg, *n* = 12; LY 600 mg, *n* = 6). Data are given as the least-squares mean (s.e.). Source data

Minimal changes in triglycerides were observed at the subtherapeutic doses of 10 mg and 30 mg, while robust reductions in triglyceride levels were observed in the 100 mg, 300 mg and 600 mg groups (Fig. 3a), with the greatest reduction occurring on day 8 in the 600 mg group (mean (s.e.) percentage change from baseline, −70.4% (5.8%)). Mean (s.e.) percentage changes in triglyceride levels were also observed on day 8 in the 100 mg group (−39.2% (11.9%)), day 15 in the 300 mg group (−58.7% (6.5%)) and day 22 in the 600 mg group (−39.7% (13.3%)). A reduction in triglyceride levels was also observed on day 2 in the 30 mg group (mean (s.e.) percentage change from baseline, −54.7% (5.8%)).

Remnant cholesterol was decreased in all groups on day 3 except in the 10 mg group (Fig. 3b). These decreases were sustained on day 8 in the 300 mg and 600 mg dose groups (mean (s.e.) percentage change from baseline, −53.4% (13.3%) and −64.8% (12.1%), respectively). On day 15 the 300 mg group had a mean (s.e.) percentage reduction in remnant cholesterol (−85.7% (13.1%)) that was the largest decrease observed in any group at any time point.

ApoC-III was reduced in all groups on day 2, with the level returning toward baseline in the 10 mg and 30 mg groups on days 3 and 4, respectively (Fig. 3c). Prolonged reduction was maintained at higher doses, and ApoC-III was maximally decreased in the 600 mg subcutaneous group on day 8 (mean (s.e.) percentage change from baseline, −52.1 (6.3%)), with reductions maintained until day 29. The ApoC-III level in the 300 mg group showed a similar response through to and including day 15 but returned toward baseline at a faster rate afterward.

Triglyceride-rich lipoprotein particles (TRL-Ps) were analyzed using nuclear magnetic resonance spectroscopy on days 15 and 22 for the 100 mg, 300 mg, 600 mg and placebo groups (Extended Data Table 2). Decreases in the concentration of large- and medium-sized TRL-Ps were observed for the higher dose groups, with the largest decreases in large- and medium-sized TRL-Ps observed on day 15. Overall, TRL-P concentration decreased in the 300 mg group on day 15 (mean (s.e.) percentage change from baseline, −47.8% (13.0%)) and in the 600 mg group on day 15 (−71.9% (14.3%)) and day 22 (−32.8% (13.8%)).

### Effects on LDL-C, non-HDL-C and ApoB

**Fig. 4:**
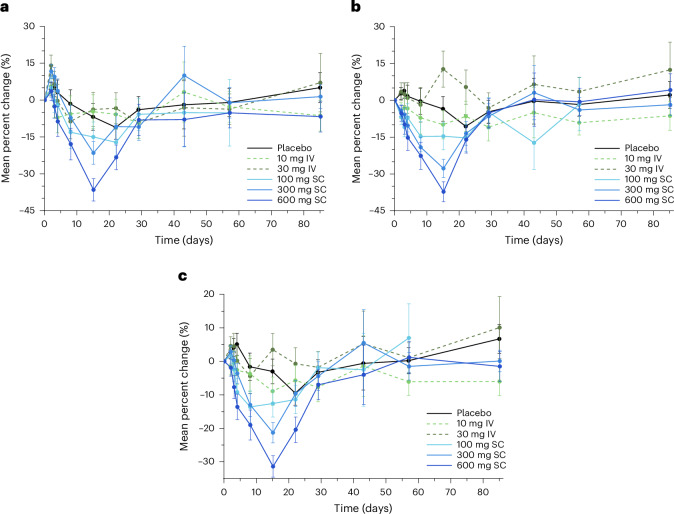
LY3475766-induced changes in LDL-C, non-HDL-C and ApoB concentration. **a**–**c**, Percent change from baseline for LDL-C (**a**), non-HDL-C (**b**) and ApoB (**c**) over time after either IV (dashed lines) or SC (solid lines) treatment with LY3475766 (placebo, *n* = 12; LY 10 mg, *n* = 6; LY 30 mg, *n* = 6; LY 100 mg, *n* = 6; LY 300 mg, *n* = 12; LY 600 mg, *n* = 6). Data are given as the least-squares mean (s.e.). Source data

Mean (s.e.) percentage reductions in LDL-C levels were observed on day 15 for participants treated with 300 mg (−15.8% (7.0%)) and 600 mg (−31.9% (6.3%)) LY3475766 (Fig. 4a). Non-HDL-C level also decreased at higher LY3475766 doses (Fig. 4b). Mean (s.e.) percentage reductions in non-HDL-C levels were observed on day 3 in the 100 mg group (−14.3% (4.6%)) and day 15 in the 300 mg group (−25.0% (5.5%)). Levels remained reduced through to and including day 15 in the 600 mg group, in which the greatest mean (s.e.) percentage reduction of non-HDL-C levels was seen (−34.9% (5.4%)). ApoB level was reduced in a dose-dependent manner with maximum reduction observed in the 600 mg group on day 15 (mean (s.e.) percentage change from baseline, −29.3% (4.3%)) (Fig. 4c). A mean (s.e.) percentage decrease in LDL particles (LDL-Ps) was observed in the 600 mg group on day 15 (−27.7% (8.3%)) and in small LDL-Ps on day 15 (−66.7% (18.1%)) and day 22 (−54.4% (18.6%)) (Extended Data Table 2).

### Effects on high-density lipoprotein

**Fig. 5:**
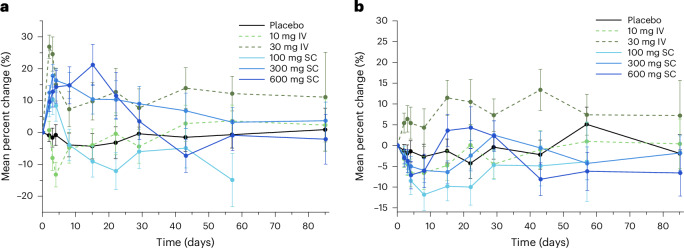
LY3475766-induced changes in HDL-C and ApoA-I concentration. **a**,**b**, Percent change from baseline for HDL-C (**a**) and ApoA-I (**b**) concentration over time after either IV (dashed lines) or SC (solid lines) treatment with LY3475766 (placebo, *n* = 12; LY 10 mg, *n* = 6; LY 30 mg, *n* = 6; LY 100 mg, *n* = 6; LY 300 mg, *n* = 12; LY 600 mg, *n* = 6). Data are given as the least-squares mean (s.e.). Source data

At the 100 mg dose there was an initial increase in HDL-C at very early time points, followed by a return to slightly below baseline at subsequent time points. Elevated HDL-C levels were observed through to and including day 15 with the 300 mg and 600 mg doses (mean (s.e.) percentage change from baseline, +15.3% (6.9%) and +26.7% (8.6%), respectively) (Fig. 5a). These changes were consistent with what would be expected based on the inverse relationship between HDL-C level and ANGPTL3/8 concentration that has previously been reported and the increased HDL-C concentration that has been reported in individuals with *ANGPTL8* LOF mutations^15,16,27,30^. No consistent trend was observed for ApoA-I (Fig. 5b). For HDL particles (HDL-Ps), mean (s.e.) percentage changes were observed in the 600 mg group, with an increase in the concentration of large HDL-Ps (+119.1% (39.6%)) on day 15 and an increase in the average size of HDL-P on day 15 (+5.6% (1.8%)) and day 22 (+2.9% (1.3%)) (Extended Data Table 2).

### Apolipoprotein profiles and lipoprotein insulin resistance

**Extended Data Fig. 3:**
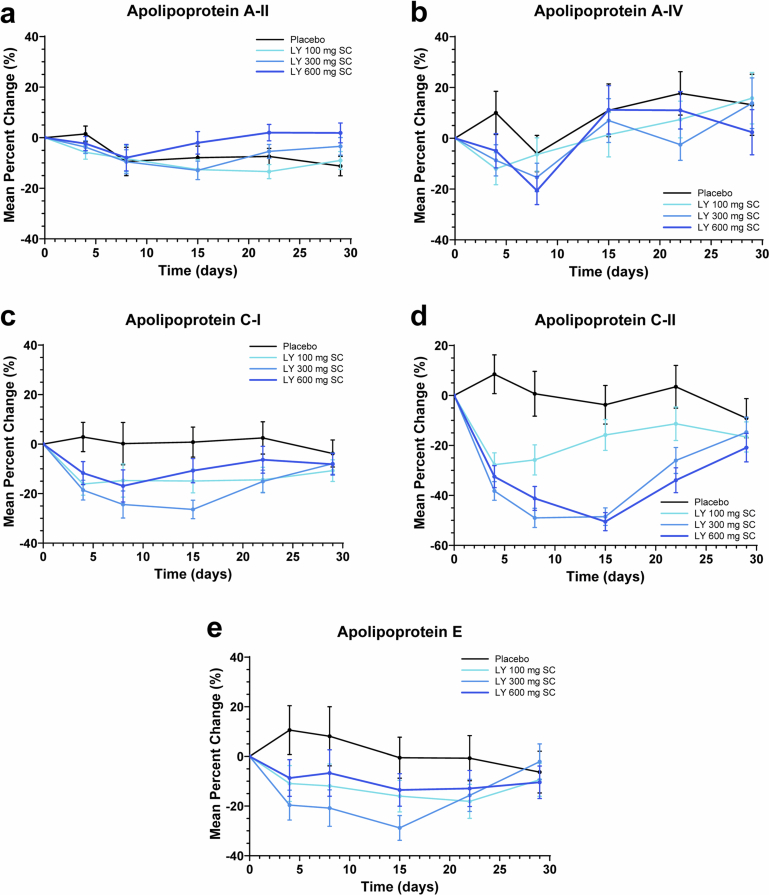
Changes in apolipoprotein concentrations in response to LY3475766 administration. Percent changes from baseline over time in (**a**) ApoA-II, (**b**) ApoA-IV, (**c**) ApoC-I, (**d**) ApoC-II, and (**e**) ApoE concentrations are shown following SC administration of LY3475766 (placebo, n = 5; LY 100 mg, n = 6; LY 300 mg, n = 7; LY 600 mg, n = 6). Data are expressed as least-squares mean (standard error). Apo, apolipoprotein; LY, LY3475766; n, number of participants; SC, subcutaneous. Source data

For all dose groups, little to no change in the levels of ApoA-II (except for the 600 mg subcutaneous group on day 29), ApoA-IV and ApoE (except for the 300 mg subcutaneous group on days 4–15) was observed in response to LY3475766 (Extended Data Fig. 3). Decreases in ApoC-I and ApoC-II levels were observed at higher doses (Extended Data Fig. 3).

**Extended Data Fig. 4:**
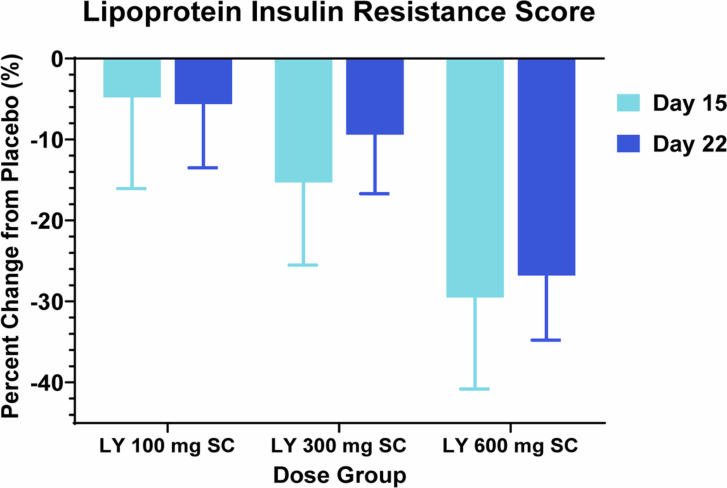
LY3475766-induced changes in lipoprotein insulin resistance scores. Percent changes in lipoprotein insulin resistance scores at day 15 following SC administration of LY3475766 (LY 100 mg, n = 6; LY 300 mg, n = 9; LY 600 mg, n = 6) and day 22 (LY 100 mg, n = 6; LY 300 mg, n = 8; LY 600 mg, n = 6) are shown for LY3475766 dose groups. Data are expressed as least-squares mean (standard error). LY, LY3475766; n, number of participants; SC, subcutaneous. Source data

The lipoprotein insulin resistance (LP-IR) score was calculated using a composite weighted score from the lipoprotein particle parameters, with changes observed relative to placebo shown in Extended Data Fig. 4. Mean (s.e.) percentage decreases in the LP-IR score were observed in the 600 mg group on day 15 (− 29.5% (11.3%)) and day 22 (− 26.8% (8.0%)).

### Cholesterol efflux capacity

**Extended Data Fig. 5:**
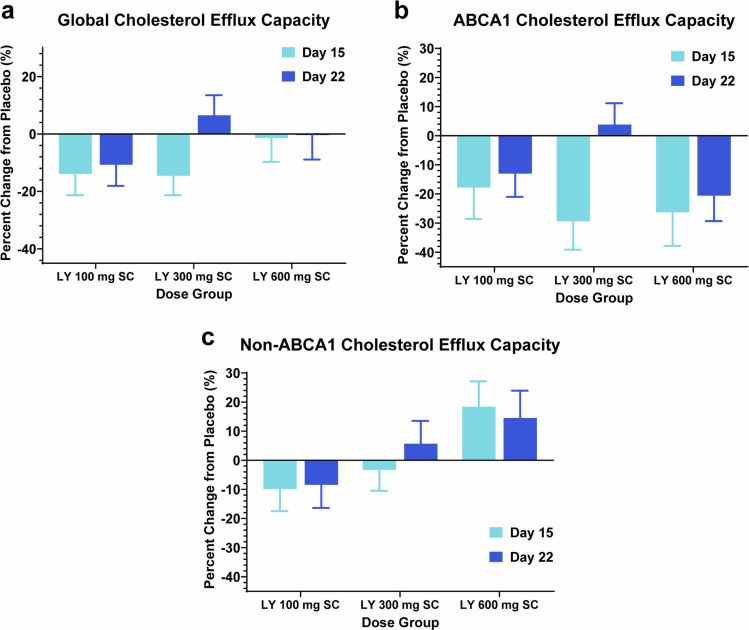
Changes in cholesterol efflux capacities following LY3475766 administration. Percent changes in (**a**) global, (**b**) ABCA1, and (**c**) non-ABCA1 cholesterol efflux capacities at day 15 following SC administration of LY3475766 (LY 100 mg, n = 5; LY 300 mg, n = 7; LY 600 mg, n = 4) and day 22 (LY 100 mg, n = 6; LY 300 mg, n = 7; LY 600 mg, n = 4) are shown for LY3475766 dose groups. Data are expressed as least-squares mean (standard error). ABCA1, ATP-binding cassette transporter A1; LY, LY3475766; n, number of participants; SC, subcutaneous. Source data

Changes in cholesterol efflux capacity were assessed in the 100 mg, 300 mg and 600 mg groups relative to placebo (Extended Data Fig. 5). Slight decreases in global cholesterol efflux were observed in the 100 mg and 300 mg subcutaneous groups on day 15. ATP-binding cassette transporter A1 (ABCA1)-dependent cholesterol efflux decreased in the 300 mg group on day 15 (mean (s.e.) percentage change from baseline, −29.4% (9.7%)) and in the 600 mg group on days 15 and 22 (−26.3% (11.5%) and −20.6% (8.7%), respectively). In comparison, a mean (s.e.) percentage increase in non-ABCA1-dependent cholesterol efflux was observed in the 600 mg subcutaneous group on day 15 (+18.4% (8.7%)).

### Lipid profile correlation analysis

Extended Data Tables 3–5 list Pearson correlation coefficients (*r*) and *P* values for correlations among changes of key measures associated with the three major lipoprotein classes (TRL, LDL and HDL) and global cholesterol efflux after treatment with LY3475766 at day 15.

## Discussion

Treatment with a single dose of the anti-ANGPTL3/8-specific monoclonal antibody LY3475766 resulted in robust, dose-dependent increases (almost ninefold) in circulating ANGPTL3/8 levels in participants with mixed hyperlipidemia. These increases occur because the antigen ANGPTL3/8 has a relatively short half-life and accumulates on LY3475766, which binds the ANGPTL3/8 complex and has a much longer half-life. Thus, these data indicate that target engagement of ANGPTL3/8 was achieved, given that ANGPTL3/8 bound to and neutralized by LY3475766 took on the relatively longer half-life of the antibody. Modest increases in ANGPTL3 observed at the higher doses of LY3475766 were likely to be due to the ANGPTL3 assay measuring total levels of ANGPTL3, including ANGPTL3 present in the ANGPTL3/8 complex. The ANGPTL3/8 complex normally circulates at much lower levels than ANGPTL3 (ref. ^15^); however, LY3475766 caused substantial dose-dependent increases in circulating ANGPTL3/8 concentrations. Consistent with these observations, LY3475766 *C*_max_ was reached 1–4.5 days after dosing and exhibited patterns suggestive of target-mediated drug disposition.

LY3475766 treatment resulted in increases in HDL-C levels (up to 26.7%) and reductions in all ApoB-containing lipid classes. LY3475766 decreased the serum concentration of triglycerides (up to −70.4%), remnant cholesterol (up to −85.7%), LDL-C (up to −31.9%), non-HDL-C (up to −34.9%), ApoB (up to −29.3%) and ApoC-III (up to −52.1%). The decreases observed in remnant cholesterol may be particularly important because elevated remnant cholesterol is perhaps more causally related to atherosclerotic CVD than is low HDL-C associated with high triglyceride levels^31^. Decreases were also observed in the total number of TRL-Ps and LDL-Ps including a reduction in small LDL-Ps (up to −66.7%) with the 600 mg dose.

The fact that the ANGPTL3/8 complex is thought to be a more potent circulating inhibitor of LPL (the key enzyme of triglyceride metabolism) than ANGPTL3 alone and acts primarily to inhibit LPL in oxidative tissues makes it an attractive target for decreasing remnant cholesterol and triglycerides in a large spectrum of phenotypes^15^. Previous in vitro experiments have shown that LY3475766, a high-affinity monoclonal antibody targeting the same active site epitope of ANGPTL3/8 that also binds LPL and ApoA-V, could reverse ANGPTL3/8-mediated inhibition of LPL^29^. In these studies, ANGPTL3/8 was found to inhibit LPL activity much more potently than ANGPTL3. In addition, ANGPTL3/8 level were found to correlate directly with LDL-C level in humans, and the ANGPTL3/8 complex blocked the ability of LPL to facilitate cholesterol-containing lipoprotein particle uptake by hepatocytes^15^, which suggests that LY3475766 should decrease LDL-C and ApoB levels in humans.

These findings prompted further preclinical studies in mice, which found that the anti-ANGPTL3/8-specific antibody LY3475766 produced marked in vivo reductions in plasma triglyceride level^29^. These results justified advancement of LY3475766 into the human trial described in the current study to establish its safety and tolerability and characterize its pharmacokinetics and pharmacodynamics. The impressive effects of LY3475766 in this study were consistent with an upregulation of LPL activity in oxidative tissues, and suggest that LY3475766 should be effective in patients with hypertriglyceridemia, provided that at least some LPL is bioavailable^6^.

At the highest dose, LY3475766 increased HDL-C level by 26.7% without affecting the level of ApoA-I, suggesting that LY3475766 may have increased the size of HDL-Ps. LY3475766 also appeared to modestly affect global cholesterol efflux, with a decrease in ABCA1-mediated cholesterol efflux counterbalanced by an increase in non-ABCA1-mediated cholesterol efflux.

The effects of ANGPTL3/8 inhibition on HDL metabolism were different from those of ANGPTL3 inhibition (as well as *ANGPTL3* LOF mutations), in which a net decrease in HDL-C has been observed^1,24,32^. Evinacumab, a monoclonal antibody described as an anti-ANGPTL3 antibody approved for treatment of homozygous familial hypercholesterolemia, was recently shown to be a more potent inhibitor of ANGPTL3/8 than of ANGPTL3^29^. Evinacumab decreases the circulating HDL-C levels, probably because evinacumab also inhibits ANGPTL3 not bound to ANGPTL8, which is an inhibitor of endothelial lipase, the enzyme that hydrolyzes phospholipids in phospholipid-rich HDL^20,33^. ANGPTL3/8 inhibits endothelial lipase to about the same extent as ANGPTL3^33^. However, ANGPTL3 is the most relevant endothelial lipase inhibitor because it is present at much higher concentrations than ANGPTL3/8. This probably explains why the reductions in HDL-C observed with ANGPTL3 inhibition were not seen with ANGPTL3/8 inhibition. Instead, the increases in HDL-C observed with ANGPTL3/8 inhibition are likely to derive from the inverse relationship between triglycerides and HDL, and the robust decreases in triglycerides observed with selective ANGPTL3/8 inhibition.

Recent data from vupanorsen, an ANGPTL3 antisense oligonucleotide (ASO), and zodasiran, an ANGPTL3 small interfering RNA (siRNA), suggest that HDL-C lowering may be a class effect with ANGPTL3 inhibitors^5,34^. In addition, the degree of LDL-C and ApoB lowering achieved with both compounds was less than that observed with evinacumab in patients with severe hypertriglyceridemia or mixed dyslipidemia despite dramatic reductions in circulating ANGPTL3 levels^3,5,34^. A reason for this might be that ANGPTL3 ASO and siRNA compounds target ANGPTL3 production in the hepatocyte, whereas evinacumab and LY3475766 are monoclonal antibodies that act at the extracellular level. Instead of inhibiting the ANGPTL3/8 complex directly, ANGPTL3 ASO and siRNA molecules indirectly inhibit the ANGPTL3/8 complex formation by decreasing ANGPTL3 availability at the source.

The question of differentiating between the clinical impacts of inhibiting a therapeutic target such as ANGPTL3 at the production site in the liver and the ANGPTL3/8 complex in the bloodstream remains up for debate. However, ANGPTL3 circulates at a much higher level than ANGPTL8, making ANGPTL3 inhibition a potentially less efficient way to reduce ANGPTL3/8 complex concentration^15^. In the current study, a single dose of LY3475766 lowered triglyceride levels by as much as 70%, compared with the approximate 36–63% reduction observed with multiple doses of vupanorsen and zodasiran, and decreased LDL-C and ApoB levels by approximately 32% and 29%, respectively, compared with the approximately 7–22% reductions observed with vupanorsen and zodasiran^5,34,35^. Interestingly, these observations align with those of a recent study that used human genetic approaches, which strongly suggested that ANGPTL3/8, rather than ANGPTL3 alone, is the key functional unit in plasma lipid regulation^36^.

Another target of interest for treating remnant cholesterol and hypertriglyceridemia is ApoC-III. The ASO olezarsen and the siRNA plozasiran directed against ApoC-III have been shown to potently decrease circulating triglycerides^37–39^. The effects on LDL-C, however, have been much less consistent compared with those observed with LY3475766 or evinacumab^37–39^. The mechanism by which ApoC-III increases triglycerides involves LPL-related and LPL-independent mechanisms but is not completely understood, given that ApoC-III ASO and siRNA molecules markedly decrease triglycerides and increase HDL-C even in patients with persistent chylomicronemia or familial chylomicronemia syndrome who have little to no functional LPL^37,38,40^. Compared with ANGPTL3 inhibitors, the triglyceride-lowering mechanism of action of LY3475766 is specifically effective against the ANGPTL3/8 complex formed postprandially and requires less bioavailable LPL after a meal than ANGPTL3 inhibitors alone to be effective, which might be of interest in the presence of persistent chylomicronemia, a cluster of postprandial disorders. ANGPTL3 inhibitors decrease ApoC-III levels mainly because of the increased clearance of ApoC-III-bound lipoproteins and remnant particles. The effect of LY3475766 on ApoC-III levels is most likely to be secondary and due to the same reason.

Of interest in this study was the reduction in the overall LP-IR score following LY3475766 treatment, which suggested that ANGPTL3/8 inhibition might improve insulin sensitivity. These data were consistent with previous observations that serum ANGPTL3/8 levels were more highly and directly correlated with fasting insulin levels in human subjects than any other biomarker that was measured^15^. Data for the 600 mg LY3475766 dose, which indicated that LY3475766 caused a robust reduction in the concentration of small LDL-Ps, were also intriguing because small LDL-Ps are known to be highly atherogenic^41^.

Further studies are needed to confirm these findings, with the next steps likely to include a multiple ascending dose study to determine the optimal dosing of LY3475766 required to achieve maximum reductions in remnant cholesterol, triglyceride, LDL-C, and ApoB levels, along with the optimal increase in HDL-C levels. A better understanding of the effects of LY3475766 on HDL-C will be particularly important because ANGPTL3/8 inhibits endothelial lipase^33^, which might lead to HDL-C increases that are less than expected. Additional studies are also needed to understand more fully the mechanisms responsible for LY3475766-induced LDL-C decreases. ANGPTL3 inhibition has been suggested to reduce LDL-C through endothelial lipase-dependent VLDL clearance^42^, however, because that study used evinacumab, which inhibits ANGPTL3/8 more potently than ANGPTL329, more investigation will be required to draw firm conclusions.

In summary, LY3475766 was well tolerated with no apparent severe adverse effects observed. A single dose of LY3475766 dose-dependently reduced the concentration of triglycerides (up to −70%), remnant cholesterol (−86%), LDL-C (−32%), non-HDL-C (−35%) and ApoB (−29%) while increasing HDL-C (+27%). These results warrant further human trials to assess the safety and efficacy of LY3475766 for the treatment of lipid disorders and for the prevention and/or treatment of atherosclerotic CVD, acute pancreatitis, or other morbidities.

## Methods

### Trial design and oversight

We conducted a phase 1, multicenter, randomized, double-blind, single ascending dose, first-in-human study. The protocol for the study J1T-MC-GZEA is provided in the Supplementary Information. In brief, participants were given LY3475766 in five single ascending dose cohorts: 10 mg intravenously, 30 mg intravenously, 100 mg subcutaneously, 300 mg subcutaneously and 600 mg subcutaneously. A 1,000 mg intravenous cohort was planned but not included as part of this study due to enrollment challenges during the COVID-19 pandemic and complications with solubility. Data from the five cohorts up to the 600 mg subcutaneous dose were considered adequate to enable assessment of safety, efficacy and durability, as well as pharmacokinetic and pharmacodynamic model development. The lower doses of 10 mg and 30 mg were given intravenously to closely monitor participants and ensure their safety during initial administrations of novel LY3475766 before proceeding to the higher doses, which were given subcutaneously. Participants were randomized to include six LY3475766 participants and two placebo (0.9% sodium chloride) participants in each cohort, and participants were followed up for 2 months after dosing. At the end of the dose treatment in each cohort, a safety review was conducted before the next cohort was given LY3475766. The trial was approved by the institutional review board or ethics committee at each site. The trial was conducted in accordance with the Declaration of Helsinki, the International Council for Harmonisation of Technical Requirements for Pharmaceuticals for Human Use Guideline for Good Clinical Practice, and applicable laws and regulations. Participants provided written informed consent. This study was registered at ClinicalTrials.gov (NCT04052594).

### Study participants

Eligible participants were overtly healthy male or female adults 18–65 years of age, with a body mass index ≥18.5 to <40 kg m^−2^, fasting triglyceride level 135–499 mg dl^−1^, and fasting LDL-C level ≥70 mg dl^−1^. The lower limit of triglycerides of 135 mg dl^−1^ was selected to facilitate efficient participant enrollment.

### Primary endpoints were safety and adverse events

Serial assessments of AEs, vital signs and results of 12-lead electrocardiograms, single-lead telemetry and clinical laboratory testing (including hematology, urinalysis, clinical chemistry and serology) were performed throughout the study. To assess immunogenicity, antidrug antibodies (ADAs) against LY3475766 were measured using an affinity capture elution bridging assay developed based on existing methods^43^. Serum for ADA measurements was collected at baseline and at regular intervals throughout the study. Treatment-emergent ADAs were defined as a titer twofold (one dilution) greater than the minimum required dilution of the assay if no ADAs had been detected at baseline (treatment-induced ADA), or a fourfold (two dilution) increase in titer compared with baseline if ADAs had been detected at baseline (treatment-boosted ADA).

### Secondary endpoints were pharmacokinetics and pharmacodynamics

Fasting plasma samples for LY3475766 were collected at 1 and 6 h and on days 3, 8, 15 and 29 after dosing, with LY3475766 concentration determined using a validated liquid chromatography–tandem mass spectrometry method with a lower limit of quantification of 50 ng ml^−1^. Pharmacokinetics were determined using noncompartmental methods (Phoenix WinNonlin version 8.1), and parameters included *C*_max_ and time to *C*_max_.

For pharmacodynamic measurements, overnight fasting samples were collected on day 1 (before dosing) and days 2, 3, 4, 8, 15, 22 and 29 after dosing to evaluate changes in triglyceride, total cholesterol, LDL-C, HDL-C, non-HDL-C, ApoA-I, ApoB and ApoC-III levels. Remnant cholesterol was calculated as total cholesterol minus LDL-C minus HDL-C. In each case, the percentage change from baseline was calculated. Because ApoA-V circulates at a much lower concentration than other apolipoproteins, immunoassays are required to measure it^44^. However, because no neutralizing antihuman ApoA-V antibodies have been described, human ApoA-V assays are susceptible to interference with ANGPTL3/8 (the levels of which increase dramatically with LY3475766 dosing). Therefore, ApoA-V level was not measured. Percent changes in lipoprotein subclasses assessed using nuclear magnetic resonance spectroscopy, changes in cholesterol efflux capacity, and changes in the LP-IR score were also evaluated^45–47^. ANGPTL3 and ANGPTL4/8 levels were measured using dedicated assays as previously described, with the ANGPTL3 assay detecting total serum level of ANGPTL3, including ANGPTL3 present in the ANGPTL3/8 complex^15^. Total circulating ANGPTL3/8 level was measured using a drug-tolerant target engagement assay based on previously described techniques^48^.

### Apolipoprotein profile and mass spectrometry

ApoA-I, ApoA-II, ApoA-IV, ApoB, ApoC-I, ApoC-II, ApoC-III and ApoE were measured using mass spectrometry. Two tryptic peptides were selected for each protein, and corresponding stable isotope-labeled peptides were synthesized. Two human serum samples with known apolipoprotein concentrations were used as external calibration standards and quality control. Four 3× serial diluted calibration standards were used to generate a standard curve for each protein. Human serum, calibration standards, or quality control samples were first diluted 20-fold using 100 mM Tris (pH 8.0). Stable isotope-labeled peptides and deoxycholate (0.4%) were added to each sample. Samples were digested using trypsin/Lys-C for 4 h at 37 °C after reduction with tris(2-carboxyethyl)phosphine and cysteine alkylated with iodoacetamide. The reaction was stopped with trifluoroacetic acid, and deoxycholate precipitates were removed using a 0.45 µm filter plate. A total of 20 µl of the sample was injected into a liquid chromatography–mass spectrometric multiple reaction monitoring system consisting of an UltiMate 3000 RSLCnano System (Thermo Scientific) coupled with a TSQ Quantiva mass spectrometer with a heated electrospray ionization source (Thermo Scientific). Peptides were separated using a 2.1 × 50 mm Hypersil GOLD column (Thermo Scientific) at a flow rate of 250 µl min^−1^, and data were analyzed using an Xcalibur Quan Browser (Thermo Scientific). The final concentration for each apolipoprotein was determined from the averaged results from the two peptides monitored.

### Nuclear magnetic resonance spectroscopy lipoprotein profiles

Lipoprotein particle concentrations and average lipoprotein particle sizes were measured using nuclear magnetic resonance spectroscopy (LabCorp). The LP-IR score was calculated using a weighted combination of six lipoprotein subclass measurements (large TRL-Ps, large HDL-Ps, small LDL-Ps, and mean size of TRL-Ps, LDL-Ps and HDL-Ps).

### Cholesterol efflux capacity

Cholesterol efflux capacities (total, ABCA1-specific and non-ABCA1-specific) were quantified after depleting serum of ApoB particles using polyethylene glycol precipitation. Non-ABCA1-specific efflux was calculated as the difference between total and ABCA1-specific efflux.

### Statistical analysis

The statistical analysis plan for the study J1T-MC-GZEA is provided in the Supplementary Information. Log-transformed *C*_max_ estimates were evaluated using a power model (in which the log dose acted as an explanatory variable) to estimate ratios of dose-normalized geometric means and corresponding 90% confidence intervals. The estimated ratio of dose-normalized geometric means of pharmacokinetic parameters between the highest and lowest subcutaneous doses was used to assess dose proportionality. Between-participant variability estimates were also assessed. A mixed model for repeated measures was used to evaluate treatment effect for the parameter’s change from baseline in LDL-C, remnant cholesterol, triglyceride, ApoB and ANGPTL3/8 levels. The model included treatment, time point and treatment × time point interaction as fixed effects, participant as a random effect, and baseline as a covariate. Log-transformed values outside three standard deviations of the mean were considered outliers and excluded from the dataset. Placebo data from each cohort were pooled across all treatment groups. Pearson correlation analysis was conducted to measure the correlation of changes from baseline at day 15 between two biomarkers. The parameters were log-transformed prior to the analysis due to the skewness of the data. The Pearson correlation test used was a two-sided test in which the null hypothesis was H0: rho = 0. It was computed as coming from a t-distribution with (*n* − 2) degrees of freedom and tested using the PROC CORR procedure in SAS with a two-sided alpha, where *n* is the sample size for each arm. Due to computational limitations in SAS, *P* values are presented in the table to only four decimal places. Data analysis was performed using SAS version 9.4.

### Reporting summary

Further information on research design is available in the Nature Portfolio Reporting Summary linked to this article.

## Online content

Any methods, additional references, Nature Portfolio reporting summaries, source data, extended data, supplementary information, acknowledgements, peer review information; details of author contributions and competing interests; and statements of data and code availability are available at 10.1038/s41591-025-03830-4.

## Supplementary information

Supplementary Information Protocol and SAP.

Reporting Summary

## Source data

Source Data Figs. 1–5 and Extended Data Figs. 2–5 Statistical source data for Figs. 1–5 and Extended Data Figs. 2–5.

## Figures and Tables

**Extended Data Table 1:**
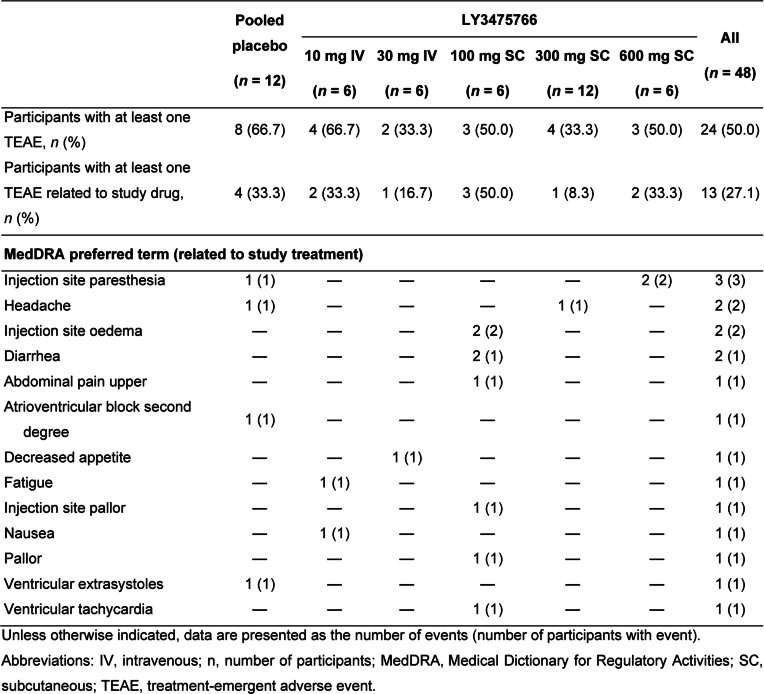
Treatment-emergent adverse events related to study treatment

**Extended Data Table 2:**
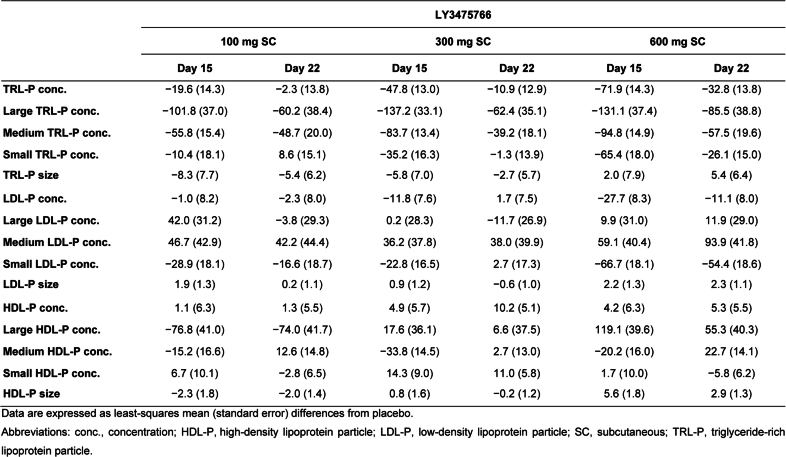
Percent changes in lipoprotein particle subclasses

**Extended Data Table 3:**
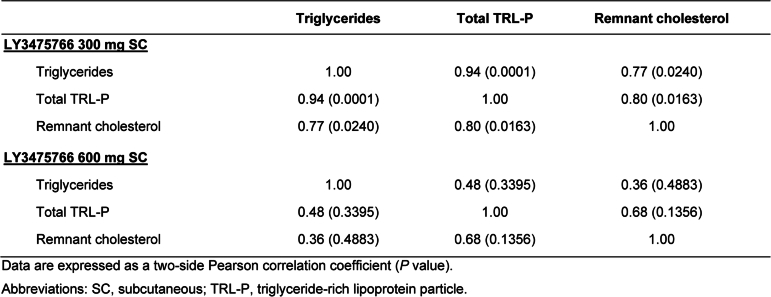
Correlation analysis of triglycerides, total TRL-P and remnant cholesterol

**Extended Data Table 4:**
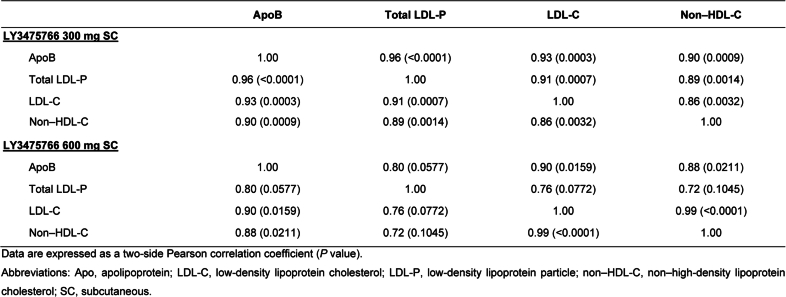
Correlation analysis of ApoB, total LDL-P, LDL-C and non-HDL-C

**Extended Data Table 5:**
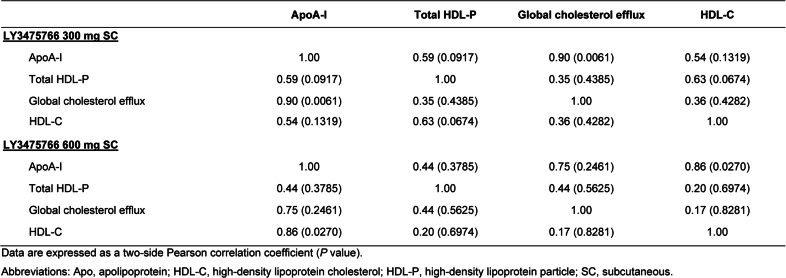
Correlation analysis of ApoA-I, total HDL-P, global cholesterol efflux and HDL-C

## Data Availability

Eli Lilly and Company provides access to all individual participant data collected during the trial, after anonymization, except for pharmacokinetic or genetic data. Data are available by request 6 months after the indication studied has been approved in the US and EU and after primary publication acceptance, whichever is later. No expiration date of data requests is currently set once data are made available. To ensure appropriate use and analysis of data, access is provided after a proposal has been approved by an independent review committee identified for this purpose and after receipt of a signed data sharing agreement and will be provided as soon as reasonably possible. Data and documents, including the study protocol, statistical analysis plan, clinical study report and blank or annotated case report forms, will be provided in a secure data sharing environment. For details on submitting a request, see the instructions provided at www.vivli.org. Source data are provided with this paper.

## Extended data

are available for this paper at 10.1038/s41591-025-03830-4.
