# Supplementary material for: Targeting the angiopoietin-like protein 3/8 complex with a monoclonal antibody in patients with mixed hyperlipidemia: a phase 1 trial
Source: Nat Med. 2025 Jul 10;31(8):2632–9. doi: 10.1038/s41591-025-03830-4 (PMC12353791; doi:10.1038/s41591-025-03830-4)
Supplement: Supplementary file 1 — Protocol and SAP. [file 41591_2025_3830_MOESM1_ESM.pdf]

nature medicine

Supplementary information https://doi.org/10.1038/s41591-025-03830-4

Targeting the angiopoietin-like protein 3/8 complex with a monoclonal antibody in patients with mixed hyperlipidemia: a phase 1 trial

In the format provided by the authors and unedited

Targeting the angiopoietin-like protein 3/8 complex with a monoclonal antibody in patients with mixed hyperlipidemia: a phase 1 trial

Daniel Gaudet, MD, PhD[1,*], Malgorzata Gonciarz, PhD[2], Xi Shen, PhD[2], Jennifer K. Leohr, PhD[2], Thomas P. Beyer, PhD[2], Jonathan W. Day, PhD[2], Garrett R. Mullins, PhD[2], Eugene Y. Zhen, PhD[2], Maryalice Hartley, PhD[2], Miriam Larouche, MSc[1], Robert J. Konrad, MD[2], Olivier Benichou, MD, PhD[2], & Giacomo Ruotolo, MD, PhD[2,*]

[1]Department of Medicine, Université de Montréal and ECOGENE-21, Chicoutimi, Québec, Canada

[2]Lilly Research Laboratories, Eli Lilly and Company, Indianapolis, IN, USA

*Corresponding author:

Dr. Daniel Gaudet, MD, PhD, can be contacted at ECOGENE-21 and Université de Montréal Community Genomics Medicine Center, 930 Jacques-Cartier E, Suite 210-B, Chicoutimi, Québec, Canada G7H 7K9. Email: daniel.gaudet@umontreal.ca.

Dr. Giacomo Ruotolo, MD, PhD can be contacted at Eli Lilly Corporate Center, Indianapolis, IN 46285, USA. Email: ruotolo_giacomo@lilly.com.

Protocol J1T-MC-GZEA(d)
A Randomized, Double-Blind, Single-Dose, Placebo-Controlled Study to Evaluate the Safety, Tolerability, Pharmacokinetics, and Pharmacodynamics of LY3475766

LY3475766

Eli Lilly and Company
Indianapolis, Indiana USA 46285

Clinical Pharmacology Protocol Electronically Signed and Approved by Lilly: 27 June 2019
Amendment (a) Electronically Signed and Approved by Lilly: 19 September 2019
Amendment (b) Electronically Signed and Approved by Lilly: 12 December 2019
Amendment (c) Electronically Signed and Approved by Lilly: 14 February 2020
Amendment (d) Electronically Signed and Approved by Lilly on approval date provided below.

Approval Date: 27-Aug-2020 GMT

Table of Contents

Protocol J1T-MC-GZEA(d) A Randomized, Double-Blind, Single-Dose, Placebo-Controlled Study to Evaluate the Safety, Tolerability, Pharmacokinetics, and Pharmacodynamics of LY3475766

Section **Page**

Protocol J1T-MC-GZEA(d) A Randomized, Double-Blind, Single-Dose, Placebo-Controlled Study to Evaluate the Safety, Tolerability, Pharmacokinetics, and Pharmacodynamics of LY3475766 1

Table of Contents 2

1. Protocol Synopsis 8
2. Schedule of Activities 10
3. Introduction 15
   3.1. Study Rationale 15
   3.2. Background 15
   3.3. Benefit/Risk Assessment 15
4. Objectives and Endpoints 16
5. Study Design 17
   5.1. Overall Design 17
   5.2. Number of Participants 20
   5.3. End of Study Definition 20
   5.4. Scientific Rationale for Study Design 20
   5.5. Justification for Dose 20
6. Study Population 24
   6.1. Inclusion Criteria 24
   6.2. Exclusion Criteria for All Participants 25
   6.3. Lifestyle and/or Dietary Requirements 28
      6.3.1. Meals and Dietary Restrictions 28
      6.3.2. Caffeine, Alcohol, and Tobacco 29
      6.3.3. Activity 29
      6.3.4. Contraceptive Requirements 29
   6.4. Screen Failures 30
7. Treatment 31
   7.1. Treatment Administered 31
      7.1.1. Packaging and Labeling 32

7.2. Method of Treatment Assignment 32
7.2.1. Selection and Timing of Doses 32
7.3. Blinding 32
7.4. Dose Modification....33
7.4.1. Dose Escalation....33
7.4.2. Special Treatment Considerations 34
7.4.2.1. Premedication for Infusions 34
7.4.2.2. Management of Infusion Reactions....34
7.5. Preparation/Handling/Storage/Accountability....35
7.6. Treatment Compliance 35
7.7. Concomitant Therapy....35
7.8. Treatment after the End of the Study 35
8. Discontinuation Criteria 36
8.1. Discontinuation from Study Treatment....36
8.1.1. Discontinuation of Inadvertently Enrolled Subjects 36
8.2. Discontinuation from the Study....36
8.3. Subjects Lost to Follow-up....36
9. Study Assessments and Procedures 37
9.1. Efficacy Assessments....37
9.2. Adverse Events 37
9.2.1. Serious Adverse Events....38
9.2.1.1. Suspected Unexpected Serious Adverse Reactions....39
9.2.2. Complaint Handling....39
9.3. Treatment of Overdose....39
9.4. Safety....39
9.4.1. Laboratory Tests 39
9.4.2. Vital Signs 39
9.4.3. Telemetry....40
9.4.4. Electrocardiograms 40
9.4.5. Injection-Site Assessments (for Subcutaneous Doses Only)....41
9.4.6. Immunogenicity Assessments 41
9.4.7. Safety Monitoring 42
9.4.7.1. Hepatic Safety 42
9.4.7.2. Hypersensitivity Reactions 42
9.5. Pharmacokinetics 43
9.5.1. Bioanalysis....43
9.6. Pharmacodynamics 43
9.6.1. Pharmacodynamic Assessments 44

9.6.1.1. Secondary and Exploratory Pharmacodynamic Endpoints....44
9.7. Genetics....44
9.8. Biomarkers....45
9.9. Health Economics....45
10. Statistical Considerations and Data Analysis....46
10.1. Sample Size Determination....46
10.2. Populations for Analyses....46
10.2.1. Study Participant Disposition....46
10.2.2. Study Participant Characteristics....46
10.3. Statistical Analyses....46
10.3.1. Safety Analyses....46
10.3.1.1. Clinical Evaluation of Safety....46
10.3.1.2. Statistical Evaluation of Safety....47
10.3.2. Pharmacokinetic Analyses....47
10.3.2.1. Pharmacokinetic Parameter Estimation....47
10.3.2.2. Pharmacokinetic Statistical Inference....47
10.3.3. Pharmacodynamic Analyses....48
10.3.3.1. Pharmacodynamic Parameter Estimation....48
10.3.3.2. Pharmacodynamic Statistical Inference....48
10.3.4. Pharmacokinetic/Pharmacodynamic Analyses....48
10.3.5. Evaluation of Immunogenicity....48
10.3.6. Data Review during the Study....48
10.3.7. Interim Analyses....49
11. References....50
12. Appendices....51

List of Tables

Table		Page
Table GZEA.1.	Objectives and Endpoints	16
Table GZEA.2.	Margin of Safety for Intravenous Administration of LY3475766 Based on Administered Dose and Predicted Exposure	22
Table GZEA.3.	Margin of Safety for Multiple Subcutaneous Administration of LY3475766 Based on Administered Dose and Predicted Exposure	23
Table GZEA.4.	Prohibited Medications that Lower Heart Rate	28
Table GZEA.5.	Treatments Administered Subcutaneously	31

List of Figures

Figure **Page**

Figure GZEA.1. Dose escalation for Protocol J1T-MC-GZEA. 18

List of Appendices

Appendix **Page**

Appendix 1. Abbreviations and Definitions 52

Appendix 2. Clinical Laboratory Tests... 56

Appendix 3. Study Governance, Regulatory, and Ethical Considerations 58

Appendix 4. Hepatic Monitoring Tests for Treatment-Emergent Abnormality 61

Appendix 5. Blood Sampling Summary... 62

Appendix 6. Protocol Amendment J1T-MC-GZEA(d) Summary
A Randomized, Double-Blind, Placebo-Controlled Study to
Evaluate the Safety, Tolerability, Pharmacokinetics, and
Pharmacodynamics of LY3475766 63

1. Protocol Synopsis

Title of Study:
A Randomized, Double-Blind, Single-Dose, Placebo-Controlled Study to Evaluate the Safety, Tolerability, Pharmacokinetics, and Pharmacodynamics of LY3475766

Rationale:
Study J1T-MC-GZEA (GZEA) aims to investigate the safety, tolerability, pharmacokinetics (PK), and pharmacodynamics (PD) of LY3475766 following single doses administered to healthy subjects. LY3475766, a human monoclonal antibody against angiopoietin-like protein (ANGPTL) 3/8 complex, is being developed to reduce the risk of cardiovascular (CV) events in patients with high triglyceride (TG) levels who are at high CV risk.

Objective(s)/Endpoints:

**Objectives**	**Endpoints**
**Primary**	
• To assess the safety and tolerability of single IV and SC doses of LY3475766 in dyslipidemic but otherwise healthy subjects.	• Incidence of AEs and SAEs<br>• Clinically significant changes in vital signs, safety laboratory parameters, and ECGs
**Secondary**	
• To characterize the PK of LY3475766 following single doses in dyslipidemic but otherwise healthy subjects.	• AUC, C_{max}, and t_{max}
• To characterize the PD of LY3475766 following single doses in dyslipidemic but otherwise healthy subjects.	• Absolute and percent change from baseline in fasting levels of TG, LDL-C, and apoB

Abbreviations: AE = adverse event; apo = apolipoprotein; AUC = area under the concentration versus time curve; C_{max} = maximum observed drug concentration; ECG = electrocardiogram; IV = intravenous; LDL-C = low-density lipoprotein cholesterol; PD = pharmacodynamic(s); PK = pharmacokinetic(s); SAE = serious adverse event; SC = subcutaneous; TG = triglycerides; t_{max} = time to maximum observed drug concentration.

Summary of Study Design:

Study GZEA is a multicenter, randomized, double-blind, single-ascending dose, first-in-human study in subjects who have elevated TG levels but are otherwise healthy.

Treatment Arms and Planned Duration for an Individual Subject:
Subjects will participate in a screening period of up to 28 days prior to Day 1.

Six cohorts will receive a single dose of LY3475766, ranging from 10 mg to 1000 mg, and be followed for up to approximately 84 days. The planned duration of the study for each subject is approximately 16 weeks.

Number of Subjects:

Up to 70 subjects may be enrolled so that 48 subjects (LY3475766: 36, Placebo: 12) have sufficient evaluable data.

Safety and Tolerability:

Safety analyses will be conducted for all enrolled subjects who take at least 1 dose of the study medication, whether or not they complete all protocol requirements.

All investigational product and protocol-procedure adverse events will be listed, and if the frequency of events allows, safety data will be summarized using descriptive methodology.

Safety parameters that will be assessed include treatment-emergent adverse events, safety laboratory parameters, vital signs, and electrocardiogram parameters.

Pharmacokinetics:

Pharmacokinetic analysis will be conducted on data from all subjects who receive at least 1 dose of the investigational product and have evaluable PK data. The primary parameters for analysis are

- maximum observed drug concentration (C_{max})
- area under the concentration versus time curve (AUC), and
- time to maximum observed drug concentration (t_{max}) of LY3475766.

Other noncompartmental parameters, such as half-life, apparent clearance, and apparent volume of distribution may be reported. Pharmacokinetic parameters will be summarized using descriptive statistics.

Pharmacodynamics:

Pharmacodynamic analysis will be conducted on data from all subjects who receive at least 1 dose of the investigational product and have evaluable PD data. Lipid biomarkers will be summarized for each dose group.

2. Schedule of Activities

Study Schedule Protocol J1T-MC-GZEA

**Procedure**	**Screening**		**Treatment Period**													**Follow-up**	**ET**	**Notes**
			**Week 1**							**Week 2**	**Week 3**	**Week 4**	**Week 5**	**Week 7**	**Week 9**	**Week 13**		
**Day**	Day -28 to Day -2	-1	1	2	3	4	5	6	7	8	15 ±2	22 ±2	29 ±2	43 ±5	57 ±5	85 ±5		
Informed consent	X																	
Review/confirm I/E criteria	X	X	X															At screening and any time between Day-1 and first dose
Randomization			X															
Admit to CRU		X																
Discharge from CRU						X				X								Discharge on Day 4 (IV cohorts) or Day 8 (SC cohorts)
Outpatient Visit										X								IV cohorts only
IP Administration			X															
Demographics	X																	
Complete physical examination	X																	Complete examination (except genital and rectal). Can be completed at either Screening or Day -1.
Directed physical examination		X	X	X	X	X	X	X	X	X	X	X	X	X	X	X	X	Symptom-directed examination; Days 5-7 for SC cohorts only
Height	X																	
Weight	X	X								X	X	X	X			X	X	Any time before breakfast

Procedure	Screening		Treatment Period													Follow-up	ET	Notes
			Week 1							Week 2	Week 3	Week 4	Week 5	Week 7	Week 9	Week 13		
**Day**	Day -28 to Day -2	-1	1	2	3	4	5	6	7	8	15 ±2	22 ±2	29 ±2	43 ±5	57 ±5	85 ±5		
BMI	X	X																
Medical history	X																	
Urine pregnancy test		X														X		Local laboratory
Screening and clinical laboratory tests	X	X	P		X					X	X		X	X	X	X	X	See Appendix 2 for details. Screening and Day -1 tests performed at local laboratories.
Screening TG and LDL-C	X	X																Local laboratory
Lipid panel			P	X	X	X				X	X	X	X	X	X	X	X	TG, total cholesterol, LDL-C, VLDL-C (calculated), HDL-C, non-HDL-C (calculated), apoA-I, apoB, and apoC-III
ANGPTL 3/8 complex			X		X					X	X		X		X	X	X	IV cohorts: On Day 1 collected at predose, end of dose, and 1 h post EoI<br>SC cohorts: On Day 1 collected at predose and 6 h postdose.
Genetic sample		X																
Stored serum samples			P			X				X	X	X	X					
PK samples for IV cohorts			P, EoI, 1 h, 6 h		X					X	X		X		X	X	X	PK sampling windows: EoI = ±5 min; 1 h = ±30 min post EoI, and 6 h = ±1 h post EoI

Procedure	Screening		Treatment Period													Follow-up	ET	Notes
			Week 1							Week 2	Week 3	Week 4	Week 5	Week 7	Week 9	Week 13		
**Day**	Day -28 to Day -2	-1	1	2	3	4	5	6	7	8	15 ±2	22 ±2	29 ±2	43 ±5	57 ±5	85 ±5		
PK samples for SC cohorts			P, 1 h, 6 h		X					X	X		X		X	X	X	PK sampling windows: 1 h = ±30 min, 6 h = ±1 h
Immunogenicity samples			P								X		X			X	X	
12-lead ECG	X		P, 6 h, 12 h	X						X			X			X	X	
Single-lead telemetry		X	X	X	X	X	X	X	X	X								Day 5 through 8 are SC only. Telemetry is continuous from 12 h predose to 72 h postdose (IV cohorts) or 168 h postdose (SC cohorts).
Seated PR	X	X	X	X	X	X	X	X	X	X	X	X	X	X	X	X	X	Day 1: Within 1 h before dosing and 5 to 15 min, 1 h (+ 30 min), and 8 h after starting dosing; Days 5-7 for SC cohorts only
Body temperature	X	X	P	X	X	X				X	X	X	X			X	X	Any time before breakfast
Seated BP	X	X	X									X		X		X	X	Day 1: 5 to 15 min and 1 h (+ 30 min) after starting dosing
AOBPM			P, 6 h	X	X	X	X	X	X	X	X		X		X			Days 5 through 7 are SC only. See Section 9.4.2. for more details.
Adverse event reporting	X	X	<===>														X	

Procedure	Screening		Treatment Period													Follow-up	ET	Notes
			Week 1							Week 2	Week 3	Week 4	Week 5	Week 7	Week 9	Week 13		
**Day**	Day -28 to Day -2	-1	1	2	3	4	5	6	7	8	15 ±2	22 ±2	29 ±2	43 ±5	57 ±5	85 ±5		
Concomitant medications	X	X	<===>														X	

Note: If multiple procedures take place at the same time point, the following order of the procedure should be used: ECG, vital signs, and venipuncture.

Abbreviations: ANGPTL = angiopoietin-like protein; AOBPM = automated office blood pressure monitoring; apo = apolipoprotein; BMI = body mass index; BP = blood pressure; CRU = clinical research unit; ECG = electrocardiogram; EoI= end of infusion; ET = early termination; h = hour(s), HDL-C = high-density lipoprotein cholesterol; I/E = inclusion/exclusion; IP = investigational product; IV = intravenous; LDL-C = low-density lipoprotein cholesterol; min = minutes; P = predose; PK = pharmacokinetic; PR = pulse rate; SC = subcutaneous; TG = triglycerides; VLDL-C = very low-density lipoprotein cholesterol.

3. Introduction

3.1. Study Rationale

LY3475766 is a human monoclonal antibody against the angiopoietin-like protein (ANGPTL) 3/8 complex. This is the first-in-human study of LY3475766, which will investigate the safety, tolerability, pharmacokinetics (PK), and pharmacodynamics (PD) of LY3475766 when administered both subcutaneously (SC) and intravenously (IV) to dyslipidemic but otherwise healthy subjects following a single dose.

3.2. Background

While statins play an important role in reducing the risk of atherosclerotic cardiovascular disease (ASCVD) events, a considerable residual risk persists among patients with ASCVD or at high risk of ASCVD. Proprotein convertase subtilisin/kexin type 9 (PCSK9) inhibitors also efficiently reduce low-density lipoprotein cholesterol (LDL-C) levels and lower the ASCVD risk, but only modestly reduce triglyceride (TG)-rich lipoprotein levels. The addition of therapies lowering TG-rich lipoproteins to standard statin treatment may mitigate this residual risk (Ganda et al. 2018).

LY3475766 binds specifically to the ANGPTL3/8 complex thereby preventing inhibition of lipoprotein lipase. As a result, LY3475766 has the potential to lower plasma TG and LDL-C levels and increase high-density lipoprotein cholesterol (HDL-C) levels. These favorable changes in the lipoprotein profile following treatment with LY3475766 may result in further reduction of ASCVD events when added to the standard of care.

Refer to the Investigator's Brochure (IB) for LY3475766 for more details.

3.3. Benefit/Risk Assessment

LY3475766 has not been administered to humans. The nonclinical safety information for LY3475766 supports the transition from preclinical status to clinical development. Based on the mechanism of action and nonclinical data, LY3475766 is not considered to be a high uncertainty compound. The only potential risk that is anticipated relates to the finding of a nondose-dependent decrease in heart rate in a 1-month Good Laboratory Practice toxicology study that was performed in cynomolgus monkeys; there were no adverse events (AEs) or any significant electrocardiogram (ECG) findings associated with the decrease in heart rate. Refer to the IB for LY3475766 for more details. This risk is considered to be monitorable and manageable at the planned doses of 10 mg, 30 mg, and 1000 mg IV and 100 mg, 300 mg, and 600 mg SC for LY3475766 in dyslipidemic but otherwise healthy subjects.

More information about the known and expected benefits, risks, serious adverse events (SAEs), and reasonably anticipated AEs of LY3475766 can be found in the IB.

4. Objectives and Endpoints

Table GZEA.1 shows the objectives and endpoints of the study.

Table GZEA.1. Objectives and Endpoints

Objectives	Endpoints
**Primary**<br>• To assess the safety and tolerability of single IV and SC doses of LY3475766 in dyslipidemic but otherwise healthy subjects.	• Incidence of AEs and SAEs<br>• Clinically significant changes in vital signs, safety laboratory parameters, and ECGs
**Secondary**<br>• To characterize the PK of LY3475766 following single doses in dyslipidemic but otherwise healthy subjects.<br>• To characterize the PD of LY3475766 following single doses in dyslipidemic but otherwise healthy subjects.	• AUC, C_{max}, and t_{max}<br>• Absolute and percent change from baseline in fasting levels of TG, LDL-C, and apoB
**Exploratory**<br>• To explore the PD effect of LY3475766 on the lipid profile of single doses in dyslipidemic but otherwise healthy subjects.	• Absolute and percent change from baseline in total cholesterol, non-HDL-C, VLDL-C, HDL-C, apoA-I, apoC-III, and fasting levels of ANGPTL3/8

Abbreviations: AE = adverse event; ANGPTL = angiopoietin-like protein; apo = apolipoprotein; AUC = area under the concentration versus time curve; C_{max} = maximum observed drug concentration; ECG = electrocardiogram; HDL-C = high-density lipoprotein cholesterol; IV = intravenous; LDL-C = low-density lipoprotein cholesterol; PD = pharmacodynamic(s); PK = pharmacokinetic(s); SAE = serious adverse event; SC = subcutaneous; TG = triglycerides; t_{max} = time to maximum observed drug concentration; VLDL-C = very low-density lipoprotein cholesterol.

5. Study Design

5.1. Overall Design

This first-in-human study will investigate the safety, tolerability, PK, and PD of LY3475766 in dyslipidemic but otherwise healthy subjects (single-ascending dose [SAD]).

Study governance considerations are described in detail in Appendix 3.

Figure GZEA.1 illustrates the study design.

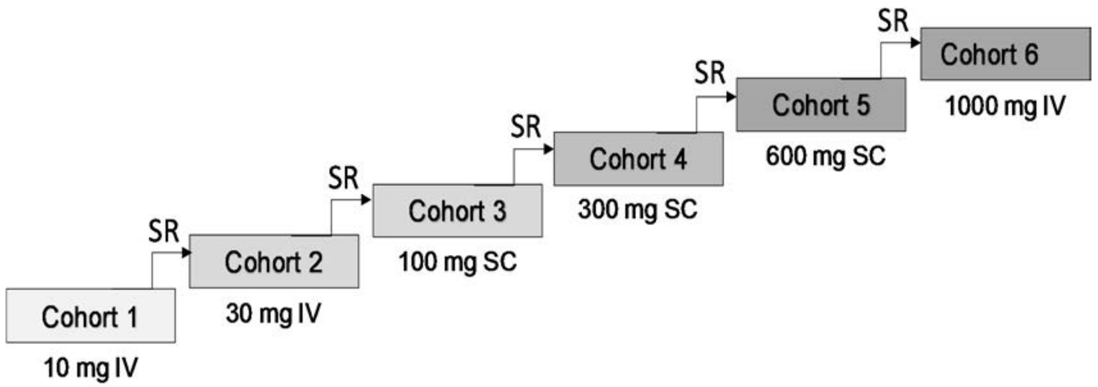

Note: For additional information, see Section 10.3.6.
Abbreviations: IV = intravenous; SC = subcutaneous; SR = safety review.

Figure GZEA.1. Dose escalation for Protocol J1T-MC-GZEA.

Potential subjects will be screened to assess their eligibility to enter the study within 28 days prior to Day 1. In case of safety concerns, participants may be required to stay at the clinical site for a longer period at the discretion of the investigator. Subjects in all cohorts will return to the clinical research unit (CRU) as outpatients at predetermined visits for up to approximately 84 days postdose for follow-up assessments. In case a subject cannot come to the site for 1 or several outpatient visits, home visits may potentially be performed instead, depending on the circumstances and if agreed upon between investigator and sponsor.

Subjects will be confined at the CRU:

- from Day -1 (the day before dosing) to Day 4 (72 hours postdose) for IV cohorts (with outpatient visits starting on Day 8), and
- from Day -1 (the day before dosing) to Day 8 (168 hours postdose) for SC cohorts.

This is a randomized double-blinded SAD study in dyslipidemic but otherwise healthy male and female subjects investigating the safety, tolerability, PD, and PK of LY3475766 versus placebo.

Subjects will be enrolled in 6 cohorts:

- Cohort 1: 10 mg LY3475766 or placebo IV
- Cohort 2: 30 mg LY3475766 or placebo IV
- Cohort 3: 100 mg LY3475766 or placebo SC
- Cohort 4: 300 mg LY3475766 or placebo SC
- Cohort 5: 600 mg LY3475766 or placebo SC
- Cohort 6: 1000 mg LY3475766 or placebo IV

Safety and tolerability will be assessed throughout the study by means of vital sign measurements, safety laboratory tests, ECGs, physical examinations, automated office blood pressure monitoring (AOBPM), and AE recording. Sampling schedules, doses, and number of cohorts may be adjusted based on ongoing review of the PK, safety, and tolerability data. Additional follow-up visits may occur depending upon the PK and/or safety data.

Doses and the number of cohorts may be adjusted based on ongoing review of the PK, safety, and tolerability data at visits described in Section 2.

Data to be reviewed prior to dose-escalation decisions, including the timing and scope of these reviews, are described in Section 7.4.1. The escalation of doses is based on the area under the concentration versus time curve (AUC) exposures to maintain approximately 3-fold between each escalating dose level except at the highest dose where the increment will be lower than 3-fold. The bioavailability (F) of an SC-administered monoclonal antibody is generally between 50% and 80%; thus, 100 mg and 300 mg SC are expected to be equivalent to 50 mg to 80 mg IV, and 150 mg to 240 mg IV in AUC, respectively.

5.2. Number of Participants

Up to 70 subjects may be enrolled so that 48 subjects (LY3475766: 36, Placebo: 12) have sufficient evaluable data.

For purposes of this study, a subject completes the study when all scheduled procedures shown in the Schedule of Activities have been completed.

Subjects who are randomized and who are discontinued from the study (providing that discontinuation did not result from a safety finding) may be replaced to ensure that enough subjects complete the study.

5.3. End of Study Definition

End of the study is the date of the last visit or last scheduled procedure shown in the Schedule of Activities (Section 2) for the last subject.

5.4. Scientific Rationale for Study Design

A subject- and investigator-blinded, randomized, placebo-controlled design has been chosen to minimize bias in the primary objective of this study.

The study is intended to estimate a maximum tolerated dose (MTD) or establish that doses exceeding the expected therapeutic dose are tolerated. Safety, tolerability, PK, and preliminary PD data will assist in identifying an appropriate dose range for subsequent clinical studies.

While recently published European Medicines Agency guideline (EMA 2017) recommends use of sentinel dosing in first-in-human studies, it also allows for flexibility in a proposed dosing approach based on the available scientific data and preclinical assessment of a given molecule. The intended and exaggerated pharmacological responses of LY3475766 have been well characterized in multiple preclinical pharmacology models. Toxicology studies have suggested that non-monitorable or clinically unmanageable concerns would be unlikely to occur in humans treated with LY3475766. Based on the available data, LY3475766 does not present an uncertainty profile necessitating a sentinel dosing approach. Furthermore, sentinel dosing with low-risk compounds, such as LY3475766, may lead to inability to interpret data due to false-positive AE findings in the absence of data from all cohorts or placebo-treated subjects.

Dyslipidemic but otherwise healthy subjects have been chosen to evaluate preliminary PD parameters in a potentially responsive population. Utilizing this subject population mitigates possible confounding effects of comorbidities and concomitant medications. Therefore, this study will provide the most unbiased assessment of the safety and tolerability while allowing for assessment of PD parameters.

5.5. Justification for Dose

Doses of 10, 30, and 1000 mg IV and 100, 300, and 600 mg SC LY3475766 were selected based on preclinical pharmacology and toxicology data.

The doses planned for this study were selected based on nonclinical toxicology studies (Table GZEA.3) and analysis of preclinical PK/PD data, with the objective of providing acceptable safety margins while allowing the identification of potentially efficacious dose levels. Preclinical PK and PK/PD data from a mouse model of elevated TG levels (cholesteryl ester transfer protein/apolipoprotein [apo] A1 double transgenic) were used to estimate a minimal anticipated biological effect level (MABEL) dose of 0.3 mg/kg IV in humans. This dose level is projected to produce less than a 10% reduction in TG at the nadir following a single dose.

LY3475766 is a humanized immunoglobulin (Ig) G4 monoclonal antibody that binds to the ANGPTL3/8 complex and thus is not expected to have any direct interaction with the immune system. Due to the anticipated low risk nature of LY3475766 and its target, it is appropriate to use the starting dose of 10 mg IV (0.14 mg/kg for a 70-kg person) in this study, which is about one-half of the estimated MABEL. The exposures (AUC) at the rat and monkey no-observed-adverse-effect levels (NOAELs) (100 mg/kg and 150 mg/kg, respectively) provide exposure multiples of 520X and 687X, respectively, relative to the projected exposure at the proposed human starting dose (Table GZEA.2).

The anticipated exposure multiples at the planned maximum human dose of 1000 mg IV (14.3 mg/kg) relative to the nonclinical NOAEL exposures are 5X and 7X for the rat and monkey, respectively (Table GZEA.2). This maximal dose was chosen to provide a robust exploration of dose response, including durability of response, while maintaining a margin of safety to the toxicology findings.

Table GZEA.2. Margin of Safety for Intravenous Administration of LY3475766 Based on Administered Dose and Predicted Exposure

	Dose (mg/kg)[a]	AUC (µg•h/mL)	Dose Multiple[b]		Exposure Multiple[b]	
			Proposed human starting dose	Maximum planned human dose	Proposed human starting dose	Maximum planned human dose
**Human**<br>Proposed starting dose (10 mg)<br>Maximum planned dose (1000 mg)	 0.14<br>14.3	 1324.9[c]<br>132485.9[c]				
**Rat[d]**<br>NOAEL	100	394,000	714×	7×	520×	5×
**Monkey[e]**<br>NOAEL	150	910,000	1071×	10×	687×	7×

Abbreviations: AUC = area under the concentration versus time curve; $AUC_{0\text{-}inf}$ = AUC from time zero to infinity; AUC_{SS} = AUC at steady state; h = hour; IV = intravenous; NOAEL = no-observed-adverse-effect level.

a Human doses expressed in mg/kg were based on a 70-kg person.

b Dose multiple is the dose in animals/dose in humans based on mg/kg. Exposure multiple is the measured AUC in animals/predicted AUC in humans. The exposure multiple is calculated using (AUC_{ss}, rat/96h)/$AUC_{0\text{-}inf}$, human/168h) for rat and (AUC_{ss}, monkey/168h)/$AUC_{0\text{-}inf}$, human/168h) for monkey.

c Plasma pharmacokinetics shown were estimated for a single dose of LY3475766 in humans. Doses shown are the proposed starting dose and the maximum planned clinical IV dose in Study J1T-MC-GZEA.

d The rat NOAEL was determined in a 1-month repeat-dose toxicity study (Study 8393896). The exposure data at the rat NOAEL were obtained at steady state from rats treated with LY3475766 100 mg/kg IV twice weekly.

e The monkey NOAEL was determined in a 1-month repeat-dose toxicity study (Study 8393897). The exposure data at the monkey NOAEL were obtained at steady state from monkeys treated with LY3475766 150 mg/kg IV once weekly.

Table GZEA.3. Margin of Safety for Multiple Subcutaneous Administration of LY3475766 Based on Administered Dose and Predicted Exposure

	Dose (mg/kg)[a]	AUC_{ss} (µg•h/mL)	Exposure Multiple[b]	
			Proposed human starting dose	Maximum planned human dose
**Human**				
Proposed starting dose (100 mg)	1.4	9911.0[c]		
Maximum planned dose (300 mg)	4.2	29796.7[c]		
**Rat[d]** NOAEL	100	394,000	139×	46×
**Monkey[e]** NOAEL	150	910,000	184×	61×

Abbreviations: AUC = area under the concentration versus time curve; $AUC_{0\text{-}inf}$ = AUC from time zero to infinity; AUC_{SS} = AUC at steady state; h = hour; IV = intravenous; NOAEL = no-observed-adverse-effect level.

a Human doses expressed in mg/kg were based on a 70-kg person.

b Dose multiple is the dose in animals/dose in humans based on mg/kg. Exposure multiple is the measured AUC in animals/predicted AUC in humans. The exposure multiple is calculated using (AUC_{SS}, rat/96h)/$AUC_{0\text{-}inf}$, human/168h) for rat and (AUC_{SS}, monkey/168h)/$AUC_{0\text{-}inf}$, human/168h) for monkey.

c Plasma pharmacokinetics shown were estimated for multiple SC doses of LY3475766 in humans at the proposed starting dose and the maximum planned clinical dose in Study J1T-MC-GZEA.

d The rat NOAEL was determined in a 1-month repeat-dose toxicity study (Study 8393896). The exposure data at the rat NOAEL were obtained at steady state from rats treated with LY3475766 100 mg/kg IV twice weekly.

e The monkey NOAEL was determined in a 1-month repeat-dose toxicity study (Study 8393897). The exposure data at the monkey NOAEL were obtained at steady state from monkeys treated with LY3475766 150 mg/kg IV once weekly.

6. Study Population

Eligibility of subjects for the study will be based on the results of screening medical history, physical examination , vital signs, clinical laboratory tests, and ECGs.

The nature of any conditions present at the time of the physical examination and any preexisting conditions will be documented. Screening may occur up to 28 days prior to enrollment. Subjects who are not enrolled within 28 days of screening may be subjected to an additional medical assessment and/or clinical measurements to confirm their eligibility. Prospective approval of protocol deviations to recruitment and enrollment criteria, also known as protocol waivers or exemptions, is not permitted.

6.1. Inclusion Criteria

Subjects are eligible for inclusion in the study only if they meet all of the following criteria at screening and/or enrollment:

[1] are overtly healthy males or females, apart from dyslipidemia, as determined through medical history and physical examination

[1a] male subjects must agree to adhere to contraception restrictions specified in Section 6.3.4

[1b] female subjects must be of nonchildbearing potential and include those who are infertile due to surgical sterilization (such as hysterectomy, bilateral salpingectomy, bilateral tubal ligation, or bilateral oophorectomy with verbal confirmation from the subject), congenital anomaly such as Mullerian agenesis, or those who are postmenopausal, defined as:

i. a woman at least 40 years of age with an intact uterus, not on hormone replacement therapy, who has had cessation of menses for at least 1 year or at least 6 months of spontaneous amenorrhea with a follicle-stimulating hormone level >40 mIU/mL; or

ii. a woman at least 55 years of age not on hormone replacement therapy who has had at least 6 months of spontaneous amenorrhea; or

iii. a woman of at least 55 years of age who has a diagnosis of menopause before starting hormone replacement therapy

[2] are between 18 and 65 years of age, inclusive

[3] have a body mass index (BMI) $\geq$18.5 and <40 kg/m^2

[4] have clinical laboratory test results within normal reference range for the population or investigative site, or results with acceptable deviations that are judged to be not clinically significant by the investigator; results will be based on laboratory samples drawn at the screening visit and on Day -1

[5] have venous access sufficient to allow for blood sampling and for IV administration as per the protocol

[6] are reliable and willing to make themselves available for the duration of the study and are willing to follow study procedures

[7] are able and willing to give signed informed consent

[8] have a fasting TG level that is 135 to 499 mg/dL, inclusive, at both screening and Day -1

[9] have a fasting LDL-C level ≥70 mg/dL at screening and Day -1

[10] have had a stable body weight for the 3 months prior to randomization (<5% body weight change)

[11] have not modified diet or adopted any nutritional lifestyle modification within 3 months of randomization

[12] Inclusion criterion [12] has been deleted.

6.2. Exclusion Criteria for All Participants

Subjects will be excluded from study enrollment if they meet any of the following criteria at screening and/or enrollment:

[13] are taking or have started taking PCSK9 inhibitors within 3 months prior to randomization; or statins, fibrates, or niacin ≤1000 mg/day within 8 weeks prior to randomization; or niacin >1000 mg/day within 16 weeks prior to randomization; or any other lipid-lowering agents (including dietary supplements or nutraceuticals containing polyunsaturated ω-3 fatty acids, red yeast rice extracts, berberine, plant sterols and stanols, soluble fibers, garlic extracts, pantethine, green tea extracts, and niacin) within 1 month prior to randomization

[14] are taking or have started taking a beta-blocker, antiarrhythmic, non-dihydropyridine calcium channel blocker, ivabradine, or any other heart rate lowering agent (see Table GZEA.4) within 1 month or 5 half-lives prior to randomization, whichever is longer

[15] intend to use over-the-counter medication in the 7 days prior to dose administration or prescription medication in the 14 days prior to dose administration (including any medications for the treatment of obesity), with the exception of hormone replacement therapy, thyroid replacement medications, antihypertensive medications (unless not allowed due to the effects on heart rate as described in exclusion criterion [14]), vitamin and mineral supplements, and occasional use of acetaminophen. If any of the above medications are taken, an otherwise suitable subject may be included following agreement between the investigator and the sponsor. If any antihypertensive medications are taken, no more than 2 such concomitant medications are allowed. For this purpose, individual components of a combination drug (for example, diuretic + calcic inhibitor) count as separate drugs.

[16] have a diagnosis of diabetes mellitus (type 1 diabetes mellitus or type 2 diabetes mellitus) or have 1 of the following at screening:

- fasting plasma glucose concentration ≥126 mg/dL (7.0 mmol/L)
- a glycated hemoglobin level ≥6.5% (48 mmol/mol)

[17] are investigative site personnel directly affiliated with this study and their immediate families. Immediate family is defined as a spouse, biological or legal guardian, child, or sibling.

[18] are Lilly employees or are employees of a third-party organization involved with the study that requires exclusion of its employees

[19] are currently enrolled in a clinical study involving an investigational product or any other type of medical research judged not to be scientifically or medically compatible with this study

[20] have participated, within the past 30 days, in a clinical study involving an investigational product. If the previous investigational product has a long half-life, 5 half-lives or 30 days (whichever is longer) should have passed before randomization in the present study

[21] have previously completed or withdrawn from this study or any other study investigating LY3475766, and have previously received the investigational product

[22] have known allergies to LY3475766, related compounds, or any components of the formulation, or history of significant atopy

[23] have significant allergies to humanized monoclonal antibodies

[24] have a seated heart rate ≤50 beats per minute

[25] have clinically significant abnormal ECG results constituting a risk while taking the investigational product, as determined by the investigator, such as a risk related to bradycardia (for example, advanced atrioventricular block, sick sinus syndrome, prolonged PR interval ≥220 msec). If an ECG parameter is out of range, the site may perform 2 repeat ECGs and average the 3 results.

[26] have a history or presence of cardiovascular, respiratory, hepatic, renal, gastrointestinal, endocrine, hematological, or neurological disorders constituting a risk while taking the investigational product, or capable of interfering with the interpretation of data, or of significantly altering drug absorption, metabolism, or elimination

[27] have an abnormal blood pressure (BP) as determined by the investigator. In case BP appears to be abnormally high during the screening period, it is possible to repeat the measure(s) one time in a quiet room to avoid an exclusion due to the white coat effect.

[28] have a significant history of or current psychiatric disorders

[29] regularly use known drugs of abuse and/or show positive findings on drug screening

[30] show evidence of human immunodeficiency virus (HIV) infection and/or positive human HIV antibodies

[31] show evidence of current hepatitis C (that is, test positive for anti-hepatitis C antibody with confirmed presence of hepatitis C virus [HCV] RNA)

Note: Patients with a previous diagnosis of hepatitis C who have been treated with antiviral therapy and achieved a sustained virological response may be eligible for inclusion in the study, provided that they have no detectable HCV RNA on the screening HCV polymerase chain reaction test for this protocol. A sustained virological response is defined as an undetectable HCV RNA level 24 weeks after completion of a full, documented course of an approved antiviral therapy for HCV.

Patients who have spontaneously cleared HCV infection, defined as (1) a positive HCV antibody test and (2) a negative HCV RNA test, with no history of HCV antibody (anti-HCV) treatment, may be eligible for inclusion in the study, provided that they have no detectable HCV RNA at screening for this study.

[32] show evidence of current hepatitis B:

- o test positive for hepatitis B surface antigen
    - and/or
- o test positive for hepatitis B core antibody and negative for hepatitis B surface antibody

[33] have donated blood of more than 500 mL within the previous 3 months of study screening, or intend to donate blood during the course of the study

[34] have an average weekly alcohol intake that exceeds 14 units per week (males) or 7 units per week (females), or are unwilling to stop alcohol consumption from 48 hours prior to admission to and while resident at the CRU (1 unit = 12 oz or 360 mL of beer; 5 oz or 150 mL of wine; 1.5 oz or 45 mL of distilled spirits)

[35] currently smoke in excess of 10 cigarettes per day or use tobacco or nicotine substitutes (within the past 6 months of screening), or subjects who are unwilling to refrain from smoking or unable to abide by CRU restrictions

[36] have received treatment with biologic agents (such as monoclonal antibodies, including marketed drugs) within 3 months or 5 half-lives (whichever is longer) prior to randomization

[37] have clinically significant multiple or severe drug allergies, or severe posttreatment hypersensitivity reactions (including, but not limited to, erythema multiforme major, linear IgA dermatosis, toxic epidermal necrolysis, or exfoliative dermatitis)

[38] have had lymphoma, leukemia, or any malignancy within the past 5 years, except for basal cell or squamous epithelial carcinomas of the skin or in situ carcinoma of the uterine cervix that have been resected with no evidence of recurrence or metastatic disease for at least 3 years

[39] have had breast cancer within the past 10 years

[40] in the opinion of the investigator or sponsor, are unsuitable for inclusion in the study

Table GZEA.4. Prohibited Medications that Lower Heart Rate

Antihypertensive	Antiarrhythmic	Psychoactive	Other
• Beta-adrenergic receptor blockers (including beta-adrenergic blocking eye drops used for glaucoma)<br>• Clonidine<br>• Methyldopa<br>• Non-dihydropyridine calcium channel blockers<br>• Reserpine	• Adenosine<br>• Amiodarone<br>• Dronedarone<br>• Flecainide<br>• Procainamide<br>• Propafenone<br>• Quinidine<br>• Sotalol	• Donepezil<br>• Lithium<br>• Opioid analgesics<br>• Phenothiazine antiemetics and antipsychotics<br>• Phenytoin<br>• Selective serotonin reuptake inhibitors<br>• Tricyclic antidepressants	• Anesthetic drugs (propofol)<br>• Cannabis<br>• Digoxin<br>• Ivabradine<br>• Muscle relaxants (e.g., succinylcholine)

6.3. Lifestyle and/or Dietary Requirements

Throughout the study, subjects may undergo medical assessments and review of compliance with requirements before continuing in the study.

6.3.1. Meals and Dietary Restrictions

The CRU will provide subjects with standardized meals while resident at the CRU. While resident in the CRU, patients may not consume any food or caloric drinks other than that provided by the CRU. When not resident in the CRU, patients may resume their regular diet.

Subjects should fast (excluding water) for 8 hours prior to predose blood sampling, performing predose ECGs, and dose administration. Subjects should remain fasting (excluding water) for at least 4 hours postdose. Postdose ECG measurements should be taken at least 5 minutes prior to any food intake.

6.3.2. Caffeine, Alcohol, and Tobacco

While confined at the CRU, subjects are required to adhere to the CRU smoking and caffeine policy during the inpatient treatment days. Subjects should otherwise not intentionally change their consumption of tobacco- or caffeine-containing products during the study.

No alcohol will be allowed during the 24 hours before and 72 hours postdose, and 24 hours before each CRU admission and each outpatient visit, and throughout the duration of each CRU visit. Between visits, daily alcohol consumption should not exceed 2 units for males and 1 unit for females (a unit is defined in Exclusion Criterion [34], Section 6.2).

6.3.3. Activity

Subjects will refrain from strenuous exercise during the period of confinement at the CRU and will otherwise maintain their normal level of physical activity throughout the entire study (i.e., will not begin a new exercise program nor participate in any unusually strenuous physical exertion).

6.3.4. Contraceptive Requirements

Male subjects (regardless of their fertility status) with nonpregnant female partners of childbearing potential must agree to either remain abstinent (if this is their preferred and usual lifestyle), or to use condoms as well as 1 additional highly effective (<1% failure rate) method of contraception (such as combination oral contraceptives, implanted contraceptives, or intrauterine devices) or effective method of contraception (such as diaphragms with spermicide or cervical sponges) from the time of first administration of the investigational product until the last study visit or 90 days following the last dose of investigational product, whichever is longer.

Men and their partners may choose to use a double-barrier method of contraception; however, barrier protection methods without concomitant use of a spermicide are not an effective or acceptable method of contraception (each barrier method must include use of a spermicide). The use of male and female condoms as a double-barrier method is not considered acceptable due to the high failure rate when these barrier methods are combined.

Male subjects with pregnant partners should use condoms during intercourse from the time of first administration of the investigational product until the last study visit or 90 days following the last dose of investigational product, whichever is longer. Male subjects should refrain from sperm donation from the time of first administration of the investigational product until the last study visit or 90 days following the last dose of investigational product, whichever is longer.

Male subjects who chose to remain abstinent (if this is their preferred and usual lifestyle) must adhere to the contraception requirements indicated above should their circumstances change.

Male subjects who are in exclusively same-sex relationships (as their preferred and usual lifestyle) are not required to use contraception.

6.4. Screen Failures

Subjects who do not qualify at screening due to a transient minor illness (such as a cold) may be rescreened 4 or more weeks after documented resolution of symptoms. Subjects who are taking any medication or medications specified in the Exclusion Criteria (Section 6.2) may be rescreened following a sufficient washout period determined by the investigator based on the exclusion criteria. Subjects who qualify at screening and Day -1 but are not dosed for the cohort may be rescreened for a future cohort. Subjects may be rescreened if they failed under previous inclusion and exclusion criteria that have been amended in the protocol, for example, TG or LDL values that would now be considered eligible under the current Inclusion Criterion [8] or [9]. When rescreening is performed, the individual must sign a new informed consent form (ICF) and will be assigned a new study identification number.

7. Treatment

7.1. Treatment Administered

The doses to be administered are presented in Section 5.1 and Table GZEA.5.

The investigational product will be administered either via an SC injection or as a slow IV infusion (over at least 30 minutes for Cohorts 1 and 2, or at least 60 minutes for Cohort 6). For SC administration, LY3475766 and placebo will be administered at a maximum volume of 2 mL per injection. A maximum of 6 injections may be necessary to achieve higher planned dose levels.

The drug product LY3475766 is supplied for clinical trial use as 75 mg of a lyophilized powder in a glass vial. Further dilution may be needed for IV administration. See Pharmacy Instructions for more information.

Placebo will be sterile saline (0.9% NaCl). Placebo doses should be held in the pharmacy for an equivalent amount of time as is required to prepare doses of LY3475766.

Investigative sites must have resuscitation equipment, emergency drugs, and appropriately trained staff available during the injection or infusion and for at least 6 hours after subjects have completed receiving their injections or infusions.

Table GZEA.5. Treatments Administered Subcutaneously

Dose Level	LY Concentration per Vial (mg/mL)	Number of Vials	Volume per Injection (mL)	Number of Injections	Total Volume of Injection (mL)
LY 100 mg/PL	50	1.3	2.0	1	2
LY 300 mg/PL	50	5	2.0	3	6
LY 600 mg/PL	50	10	2.0	6	12

Abbreviations: LY = LY3475766; PL = placebo.

Injection site selected for SC administration should be the abdominal region approximately 5 cm from the umbilicus and the treatment has to be administered through the needle applied at approximately 45° with pinching of the skin. Because subjects may receive multiple injections per dose, each injection should be administered in a different abdominal quadrant in Cohorts 3 and 4 (rotating from right upper quadrant, to right lower quadrant, to left lower quadrant, to left upper quadrant), the quadrant used should be recorded at the time of the injection. For Cohort 5 where 6 SC injections are necessary, the injections should be administered into 6 separate regions of the abdomen: upper right, middle right (same level as umbilicus), lower right, lower left, middle left (same level as umbilicus), and upper left. Administering in a clockwise or counterclockwise direction should be considered to avoid injecting into the same location twice. The abdominal location for each injection should be recorded at the time of the injection. All injections should be performed within a total of 10 minutes. Subcutaneous administration of

LY3475766 should be done by a limited number of individuals for consistency. The same type of syringe and needle should be used for all subjects to ensure all injections are delivered to a consistent depth target into the SC space.

All clinical trial materials provided to the investigator will be stored in a secure place and dispensed by appropriately trained persons. The dispensing of the investigational product will be fully documented. Detailed records of the amounts of the investigational products received, dispensed, and remaining at the end of the study will be maintained.

The investigator or designee is responsible for

- explaining the correct use of the investigational product(s) to the subject
- verifying that instructions are followed properly
- maintaining accurate records of investigational product dispensing and collection, and
- returning all unused medications to Lilly or its designee at the end of the study.

Note: In some cases, sites may destroy the material if, during the investigative site selection, the evaluator has verified and documented that the site has appropriate facilities and written procedures to dispose of clinical materials.

7.1.1. Packaging and Labeling

LY3475766 will be provided to the investigative site as bulk supply in open-label vials by Lilly or its designee. Placebo will be provided to the investigative site as commercially available saline vials by Lilly or its designee.

Clinical trial materials will be labeled according to the country's regulatory requirements, and will be stored, inventoried, reconciled, and destroyed according to applicable regulations. Clinical trial materials are manufactured in accordance with current good manufacturing practices.

The investigational products will be labeled according to the country's regulatory requirements.

7.2. Method of Treatment Assignment

Subjects who meet all criteria for enrollment will be randomized to 1 of the study treatment groups on Day 1. Subjects will be randomized in a 3:1 ratio to receive LY3475766 or placebo.

7.2.1. Selection and Timing of Doses

The actual time of all dose administrations will be recorded in the subject's electronic case report form (eCRF).

7.3. Blinding

This is a double-blinded study; subjects and investigator site personnel will be blinded to treatment assignment (and to the results of postbaseline serum lipid levels) with the exception of the site staff preparing study drug.

Blinding will be maintained throughout the conduct of the study as described in the separate Blinding Plan. Emergency codes will be available to the investigator. A code, which reveals the treatment [group] for a specific study subject, may be opened during the study only if the subject's well-being requires knowledge of the subject's treatment assignment.

If a subject's study treatment assignment is unblinded, the subject must be discontinued from the investigational product, unless the investigator obtains specific approval from a Lilly clinical pharmacologist (CP) or clinical research physician (CRP) for the study participant to continue in the study. During the study, emergency unblinding should occur only by accessing the study subject's emergency code.

In case of an emergency, the investigator has the sole responsibility for determining if unblinding of a subject's treatment assignment is warranted for medical management of the event. The subject's safety must always be the first consideration in making such a determination. If the investigator decides that unblinding is warranted, it is the responsibility of the investigator to promptly document the decision and rationale and notify Lilly as soon as possible

Upon completion of the study, all codes must be returned to Lilly or its designee.

7.4. Dose Modification

Any available PK data may be used to guide dose selection or to determine if the number of doses to be studied may be reduced in conjunction with known safety data. Safety data, in particular AEs, SAEs, and adverse laboratory abnormalities, will be independently assessed by the investigator and will be considered related to the investigational product unless there is clear evidence that the event is not related.

7.4.1. Dose Escalation

By nature of being a dose-escalation study, data will be evaluated on an ongoing basis until the highest planned dose has been administered or the MTD is determined. If the highest planned dose is not reached, the highest dose level that is tolerated will be designated as the MTD.

Safety data will be the primary criteria for the dose escalation. In addition, if available at the time of dose escalation decision, PK results (maximum observed drug concentration [C_{max}], AUC, and total body clearance of drug calculated after IV administration) will be used as supporting data for dose escalation, but such data are not required. No dose decision can occur without prior discussion and agreement between the investigator and the Lilly CP.

After review of these data, an agreement on the appropriate dose will be made by the investigator and sponsor for the next cohort/dose level. The magnitude of dose escalations may be adjusted following data review, but subsequent escalations cannot be increased by more than approximately 3-fold (a half-log increment) in PK exposure or predicted AUC.

Dose-escalation decisions will primarily be based on available safety and tolerability data obtained up to Day 8 from Cohorts 1, 2, and 6 and up to Day 15 from Cohorts 3, 4, and 5 (Section 10.3.6). Additionally, any available PK data may be used to guide dose selection or to determine if the number of doses to be studied may be reduced.

If any of the following scenarios occur, dosing at the current level and further dose escalation will be interrupted until a further sponsor decision:

1) Three or more LY3475766-treated subjects develop AEs that are considered to be related to study treatment and graded as at least moderate, clinically significant, and not responsive to supportive care
2) One or more LY3475766-treated subject develops AEs that are considered to be related to study treatment and graded as severe
3) One or more LY3475766-treated subjects develop SAEs that are considered to be related to study treatment

In case any of the 3 scenarios above occurs at a given site (independently of treatment assignment since site investigators are blinded), this site is not allowed to continue dosing at the current level without written agreement from the sponsor.

In addition to safety reasons, the sponsor may decide to stop further dose escalation if pharmacodynamic data obtained from the previous cohorts are considered sufficient.

7.4.2. Special Treatment Considerations

7.4.2.1. Premedication for Infusions

Premedication for the infusions is not planned. However, if an infusion reaction occurs, appropriate medication may be used as determined by the study investigator(s). If infusion reactions are observed, but review of the data suggests that dose escalation may continue, administration of acetaminophen, 500 to 1000 mg and/or an antihistamine may be administered orally 30 to 60 minutes prior to the start of infusion for subsequent subjects.

The decision to implement premedication for infusions in subsequent cohorts will be made by the investigator and sponsor and recorded in the study documentation, along with the dose-escalation decision.

Any premedications given will be documented as a concomitant therapy (see Section 7.7).

7.4.2.2. Management of Infusion Reactions

There is a risk of infusion reaction with any biological agent; therefore, all subjects should be monitored closely. Symptoms and signs that may occur as part of an infusion reaction include, but are not limited to fever, chills, nausea, headache, bronchospasm, hypotension, angioedema, throat irritation, rash, pruritus, myalgia, and dizziness. In the event that a significant infusion reaction occurs, the following guidance should be followed:

- The investigational product infusion should be slowed down (for example, reduce infusion rate by 50% [for example, an infusion rate of 12 mL/h becomes 6 mL/h or slower]) or stopped, depending on the symptoms/signs present:
    - if slowed down, the infusion should be completed at the slower rate, as tolerated

    - if determined by the investigator that the infusion should no longer continue, no further attempts to dose the subject should be made

- Supportive care should be employed in accordance with the symptoms/signs

7.5. Preparation/Handling/Storage/Accountability

The investigator or designee must confirm that appropriate temperature conditions have been maintained, as communicated by the sponsor, during transit for all investigational products received and any discrepancies are reported and resolved before use of the study treatment.

Only participants enrolled in the study may receive investigational products or study materials, and only authorized site staff may supply or administer investigational products. All investigational products should be stored in an environmentally controlled and monitored (manual or automated) area in accordance with the labeled storage conditions with access limited to the investigator and authorized site staff.

The investigator, institution, or the head of the medical institution (where applicable) is responsible for study treatment accountability, reconciliation, and record maintenance (such as receipt, reconciliation, and final disposition records).

7.6. Treatment Compliance

The investigational product will be administered at the clinical site, and documentation of treatment administration will occur at the site.

Subjects who are significantly noncompliant (missed at least 1 dose of study drug) will be discontinued from the study.

7.7. Concomitant Therapy

Lipid-lowering drugs (Section 6.2, Criterion [13]) and drugs potentially expected to decrease the heart rate (Table GZEA.4) are not allowed during the study. In general, concomitant medication should be avoided; however, acetaminophen (1 g, maximum 3 g/24 hours) may be administered at the discretion of the investigator for treatment of headaches etc. Additional drugs are to be avoided during the study unless required to treat an AE or for the treatment of an ongoing medical problem (for example, estrogen/progesterone as hormone replacement therapy and thyroid medications). If the need for concomitant medication arises, inclusion or continuation of the subject may be at the discretion of the investigator in consultation with a Lilly CP or CRP. Any additional medication used during the course of the study must be documented.

7.8. Treatment after the End of the Study

Not applicable.

8. Discontinuation Criteria

Subjects discontinuing prematurely from the treatment or from the study for any reason should complete AE and other follow-up procedures per the Schedule of Activities (Section 2) of this protocol.

8.1. Discontinuation from Study Treatment

Not applicable for this the study.

8.1.1. Discontinuation of Inadvertently Enrolled Subjects

If the sponsor or investigator identifies a subject who did not meet enrollment criteria and was inadvertently enrolled, a discussion must occur between the Lilly CP and the investigator to determine if the subject may continue in the study. If both agree it is medically appropriate to continue, the investigator must obtain documented approval from the Lilly CP to allow the inadvertently enrolled subject to continue in the study with or without continued treatment with investigational product.

8.2. Discontinuation from the Study

Subjects will be discontinued under the following circumstances:

- Enrollment in any other clinical study involving an investigational product or enrollment in any other type of medical research judged not to be scientifically or medically compatible with this study
- Participation in the study needs to be stopped for medical, safety, regulatory, or other reasons consistent with applicable laws, regulations, and good clinical practice
- Investigator Decision
    - the investigator decides that the subject should be discontinued from the study
    - if the subject, for any reason, requires treatment with another therapeutic agent that has been demonstrated to be effective for treatment of the study indication, discontinuation from the study occurs prior to introduction of the new agent
- Subject Decision
    - the subject, or legal representative, requests to be withdrawn from the study.

8.3. Subjects Lost to Follow-up

A subject will be considered lost to follow-up if he or she repeatedly fails to return for scheduled visits and is unable to be contacted by the study site. Site personnel are expected to make diligent attempts to contact subjects who fail to return for a scheduled visit or were otherwise unable to be followed up by the site.

9. Study Assessments and Procedures

Section 2 lists the Schedule of Activities, detailing the study procedures and their timing (including tolerance limits for timing).

Appendix 2 lists the laboratory tests that will be performed for this study.

Appendix 5 provides a summary of the maximum number and volume of invasive samples, for all sampling, during the study.

Unless otherwise stated in subsections below, all samples collected for specified laboratory tests will be destroyed within 60 days of receipt of confirmed test results. Certain samples may be retained for a longer period, if necessary, to comply with applicable laws, regulations, or laboratory certification standards.

9.1. Efficacy Assessments

Pharmacodynamic assessments are described in Section 9.6.

9.2. Adverse Events

Investigators are responsible for monitoring the safety of subjects who have entered this study and for alerting Lilly or its designee to any event that seems unusual, even if this event may be considered an unanticipated benefit to the subject.

The investigator is responsible for the appropriate medical care of subjects during the study.

Investigators must document their review of each laboratory safety report.

The investigator remains responsible for following, through an appropriate health care option, AEs that are serious or otherwise medically important, considered related to the investigational product or the study, or that caused the subject to discontinue the investigational product before completing the study. The subject should be followed up until the event resolves, stabilizes with appropriate diagnostic evaluation, or is reasonably explained. The frequency of follow-up evaluations of the AE is left to the discretion of the investigator.

The investigator will record all relevant AE and SAE information in the eCRF. After the ICF is signed, study site personnel will record, via eCRF, the occurrence and nature of each subject's preexisting conditions, including clinically significant signs and symptoms of the disease under treatment in the study. Additionally, site personnel will record any change in the condition(s) and the occurrence and nature of any AEs.

The investigator will interpret and document whether or not an AE has a reasonable possibility of being related to study treatment or a study procedure, taking into account the disease, concomitant treatment, or pathologies.

A "reasonable possibility" means that there is a potential cause and effect relationship between the investigational product and/or study procedure and the AE.

Planned surgeries should not be reported as AEs, unless the underlying medical condition has worsened during the course of the study.

If a subject's investigational product is discontinued as a result of an AE, study site personnel must report this to Lilly or its designee via eCRF.

9.2.1. Serious Adverse Events

An SAE is any AE from this study that results in 1 of the following:

- death
- initial or prolonged inpatient hospitalization
- a life-threatening experience (that is, immediate risk of dying)
- persistent or significant disability/incapacity
- congenital anomaly/birth defect
- important medical events that may not be immediately life-threatening or result in death or hospitalization but may jeopardize the subject or may require intervention to prevent 1 of the other outcomes listed in the definition above.

Study site personnel must alert the Lilly CRP/CP, or its designee, of any SAE as soon as practically possible.

Additionally, study site personnel must alert Lilly Global Patient Safety, or its designee, of any SAE within 24 hours of investigator awareness of the event via a sponsor-approved method. If alerts are issued via telephone, they are to be immediately followed up with official notification on study-specific SAE forms. This 24-hour notification requirement refers to the initial SAE information and all follow-up SAE information.

Although all AEs are recorded in the eCRF after signing informed consent, SAE reporting to the sponsor begins after the subject has signed informed consent and has received investigational product. However, if an SAE occurs after signing informed consent, but prior to receiving investigational product, AND is considered reasonably possibly related to a study procedure then it MUST be reported.

Investigators are not obligated to actively seek AEs or SAEs in subjects once they have discontinued from and/or completed the study (the subject summary case report form has been completed). However, if the investigator learns of any SAE, including a death, at any time after a subject has been discharged from the study, and he/she considers the event reasonably possibly related to the study treatment or study participation, the investigator must promptly notify Lilly.

Pregnancy (maternal or paternal exposure to investigational product) does not meet the definition of an AE. However, to fulfill regulatory requirements any pregnancy should be reported following the SAE process to collect data on the outcome for both mother and fetus.

9.2.1.1. Suspected Unexpected Serious Adverse Reactions

Suspected unexpected serious adverse reactions (SUSARs) are serious events that are not listed in the IB and that the investigator reports as related to investigational product or procedure. Lilly has procedures that will be followed for the recording and expedited reporting of SUSARs that are consistent with global regulations and the associated detailed guidances.

9.2.2. Complaint Handling

Lilly collects product complaints on investigational products and drug delivery systems used in clinical trials to ensure the safety of study participants, monitor quality, and to facilitate process and product improvements.

Subjects should be instructed to contact the investigator as soon as possible if they have a complaint or problem with the investigational product so that the situation can be assessed.

9.3. Treatment of Overdose

For the purposes of this study, an overdose of LY3475766 is considered any dose higher than the dose assigned through randomization. The treatment for overdose is supportive care.

Refer to the IB for LY3475766.

9.4. Safety

9.4.1. Laboratory Tests

For each subject, laboratory tests detailed in Appendix 2 should be conducted according to the Schedule of Activities (Section 2).

With the exception of test results that may unblind the study, Lilly or its designee will provide the investigator with the results of safety laboratory tests analyzed by a central vendor, if a central vendor is used for the study.

9.4.2. Vital Signs

For each subject, vital sign measurements should be conducted according to the Schedule of Activities (Section 2).

Blood pressure and pulse rate should be measured after the subject has been sitting for at least 5 minutes comfortably in a quiet environment, in a chair with back support, legs uncrossed, feet touching the floor, and arm resting at heart level.

Unscheduled orthostatic vital signs should be assessed, if possible, during any AE of dizziness or posture-induced symptoms. If orthostatic measurements are required, subjects should be supine for at least 5 minutes and stand for at least 2 minutes. If the subject feels unable to stand, supine vital signs only will be recorded. Additional vital signs may be measured if considered necessary by the investigator.

Body temperature will be measured, as specified in the Schedule of Activities (Section 2), and as clinically indicated.

The AOBPM will be performed according to the Schedule of Activities (Section 2). Site investigative personnel should be trained on the correct positioning of the AOBPM device, use of correct cuff size, and monitor calibration prior to the start of the study.

The subject should avoid eating, drinking (except water), or smoking 30 minutes before AOBPM. The subject is seated comfortably in a chair with back support, legs uncrossed, feet touching the floor, and arm resting at heart level.

The AOBPM will be performed by CRU staff that have appropriate training in collecting BP. After resting for 5 minutes, the BP measurements will occur.

Four BP readings are recorded 1 minute apart; the latter 3 are averaged, and the first reading is discarded. The average of the 3 BP measurements is considered for analysis.

The AOBPM done after Day 1 should be done at approximately the same time of day as predose and done under similar conditions.

9.4.3. Telemetry

For each subject, single-lead ECG telemetry will be performed during each stay at the CRU involving at least 1 night (see Schedule of Activities in Section 2). The telemetry system has standard alarms to alert the clinical staff to any detected abnormalities, for example, cardiac arrhythmias, prolongation of QT, during which additional 12-lead ECGs may be obtained at the investigator's discretion. These may also be performed in the event of technical or logistical impediments to the use of telemetry. In order to bathe, subjects may remove the telemetry device for up to 1 hour, which should be documented.

9.4.4. Electrocardiograms

For each subject, a single 12-lead digital ECG will be collected according to the Schedule of Activities (Section 2). Electrocardiograms must be recorded before collecting any blood samples. Subjects must be supine for approximately 5 to 10 minutes before ECG collection and remain supine but awake during ECG collection. Electrocardiograms may be obtained at additional times, when deemed clinically necessary. All ECGs recorded should be stored at the investigational site.

Electrocardiograms will be interpreted by a qualified physician at the site as soon after the time of ECG collection as possible, and ideally while the subject is still present, to determine whether the subject meets entry criteria at the relevant visit(s) and for immediate subject management, should any clinically relevant findings be identified. The screening ECG may be initially reviewed by a qualified non-physician designated by the investigator but must be reviewed by a physician to confirm eligibility before randomization.

Any clinically significant findings from ECGs that result in a diagnosis and that occur after the subject receives the first dose of the investigational product should be reported to Lilly, or its designee, as an AE via eCRF.

If a clinically significant finding is identified (including, but not limited to, changes in QT/QT interval corrected for heart rate [QTc] interval from baseline) after enrollment, the investigator will determine if the subject can continue in the study. The investigator, or qualified designee, is responsible for determining if any change in subject management is needed, and must document his/her review of the ECG printed at the time of collection.

Collection of ECG replicates at a particular time point will be permitted to ensure high-quality records.

Starting at Day 1, all digital ECGs (scheduled and unscheduled) will be electronically transmitted to a central ECG laboratory designated by Lilly. The central ECG laboratory will perform a basic quality control check (for example, demographics and study details) and then store the ECGs in a database. At a future time, the stored ECG data may be overread at the central ECG laboratory for further evaluation of machine-read measurements or to meet regulatory requirements.

The machine-read ECG intervals and heart rate may be used for data analysis and report writing purposes unless a cardiologist overread of the ECGs is conducted prior to completion of the final study report (in which case the overread data would be used).

9.4.5. Injection-Site Assessments (for Subcutaneous Doses Only)

If an AE of injection-site reaction (ISR) is reported by SC dosing cohorts, a form will be triggered in the eCRF to capture specific information about this reaction (for example, erythema, induration, categorical pain, pruritus, edema). If there is more than 1 symptom of an ISR that meets the definition of an AE (for instance erythema and pruritus), a single AE of ISR will be recorded on the AE page of the eCRF.

9.4.6. Immunogenicity Assessments

At the visits and times specified in the Schedule of Activities (Section 2), serum samples will be collected and stored for potential future analysis to determine antibody production against LY3475766. Antibodies may be further characterized for their ability to neutralize the activity of LY3475766. To interpret the results of immunogenicity, a venous blood sample will be collected at the same time points to determine the plasma concentrations of LY3475766. All samples for immunogenicity should be taken predose when applicable and possible.

Treatment-emergent anti-drug antibodies (TE-ADAs) are defined in Section 10.3.5. In the case that anti-drug antibody (ADA) samples are tested before end of study and the immunogenicity sample at the last scheduled assessment or discontinuation visit is treatment-emergent positive, additional samples may be taken until the signal returns within 2-fold of baseline signal, or up to 1 year.

Samples will be retained for a maximum of 15 years after the last subject visit, or for a shorter period if local regulations and ethical review boards (ERBs) allow, at a facility selected by the sponsor. The duration allows the sponsor to respond to future regulatory requests related to the LY3475766. Any samples remaining after 15 years will be destroyed.

Every attempt should be made to contact subjects for the follow-up immunogenicity assessment; however, if subjects are unwilling or unable to return for the visit, this is not considered a protocol deviation.

9.4.7. Safety Monitoring

The Lilly CP or CRP/scientist will monitor safety data throughout the course of the study.

Lilly will review SAEs within time frames mandated by company procedures. The Lilly CP or CRP will periodically review the following data:

- trends in safety data
- laboratory analytes
- AEs

When appropriate, the Lilly CP or CRP will consult with the functionally independent Global Patient Safety therapeutic area physician or clinical research scientist.

In the event that safety monitoring uncovers an issue that needs to be addressed by unblinding at the group level, additional analyses of the safety data will be conducted by the personnel included in the separate Blinding Plan.

9.4.7.1. Hepatic Safety

If a study subject experiences elevated alanine aminotransferase (ALT) ≥3× upper limit of normal (ULN), alkaline phosphatase (ALP) ≥2× ULN, or elevated total bilirubin level (TBL) ≥2× ULN, liver tests (Appendix 4) should be repeated within 3 to 5 days including ALT, AST, ALP, TBL, direct bilirubin, gamma-glutamyl transferase, and creatinine kinase to confirm the abnormality and to determine if it is increasing or decreasing. If the abnormality persists or worsens, clinical and laboratory monitoring should be initiated by the investigator based on consultation with the Lilly CP or CRP. Monitoring should continue until levels normalize and/or are returning to approximate baseline levels.

Additional safety data should be collected if 1 or more of the following conditions occur:

- elevation of serum ALT to ≥5× ULN on 2 or more consecutive blood tests
- elevation of serum TBL to ≥2× ULN (except for cases of known Gilbert’s syndrome)
- elevation of serum ALP to ≥2× ULN on 2 or more consecutive blood tests
- subject discontinued from treatment due to a hepatic event or abnormality of liver tests
- hepatic event considered to be an SAE.

9.4.7.2. Hypersensitivity Reactions

Standard of care should be used in case of any hypersensitivity reaction. In the event of anaphylaxis or generalized urticaria, additional laboratory samples should be collected as close to the event as possible (ideally in 1 to 2 hours and no more than 12 hours after the event) to evaluate tryptase, complement levels, cytokines, ADA, and PK, whenever possible. If a tryptase

sample is not obtained in 1 to 2 hours of the event, urine collection (24-hour urine collection or spot urine sample) for N-methylhistamine testing should be obtained. Follow-up samples to re-evaluate those measurements should be obtained at the next regularly scheduled visit or after 4 weeks, whichever is later. Specific instructions for the collection and handling of samples will be provided by the sponsor.

9.5. Pharmacokinetics

At the visits and times specified in the Schedule of Activities (Section 2), venous blood samples of approximately 3 mL each will be collected to determine the plasma concentrations of LY3475766. The proposed sampling schedule may be modified during the study based on the results from the interim PK data snapshots. The sampling duration may be extended if required.

A maximum of 3 samples may be collected at additional time points during the study if warranted and agreed upon between both the investigator and sponsor. Drug concentration information that would unblind the study will not be reported to study sites or blinded personnel.

Blood samples are requested to be taken at the specified time. However, deviations from the specified sampling times will not be considered protocol deviations as long as the samples are taken and the actual sampling time is recorded. It is essential that the actual times of doses and samples are recorded accurately on the appropriate forms.

Instructions for the collection and handling of blood samples will be provided by the sponsor.

When there is a scheduling conflict between PK sample collection and other study activities, PK samples are to take priority over all other study activities and should be collected at the nominal time with ECGs and vital signs (in that order) to be collected prior to blood collection, except for safety interventions in the case of an AE. The actual time of PK sample collection should be recorded to the nearest minute, as close as possible to the times shown in Schedule of Activities (Section 2). The timing of ECG and vital sign recording (and other study activities) can be altered slightly, if necessary to accommodate PK sampling.

9.5.1. Bioanalysis

Samples will be analyzed at a laboratory approved by the sponsor and stored at a facility designated by the sponsor.

Concentrations of LY3475766 will be assayed using a validated enzyme-linked immunosorbent assay. Analyses of samples collected from subject who receive placebo are not planned.

Bioanalytical samples collected to measure investigational product concentrations will be retained for a maximum of 1 year following the last subject visit for the study. During this time, samples remaining after the bioanalyses may be used for exploratory analyses such as bioanalytical assay validation or cross-validation exercises.

9.6. Pharmacodynamics

Supplies required for the collection and shipment of the subjects' samples will be provided by

the sponsor. Sample handling and shipment to the central laboratory will occur per instructions given to the study site. The sample(s) will be identified by the subject number (coded) and stored for up to a maximum of 15 years after the last subject visit for the study at a facility selected by the sponsor.

9.6.1. Pharmacodynamic Assessments

9.6.1.1. Secondary and Exploratory Pharmacodynamic Endpoints

At times specified in the Schedule of Activities (Section 2), venous blood samples will be collected and used to determine the PD effects of LY3475766. Fasting blood samples will be taken to evaluate the effects of LY3475766 on the following PD parameters:

- TG
- total cholesterol, LDL-C, and HDL-C
- calculated non-HDL-C and very low-density lipoprotein cholesterol (VLDL-C)
- apolipoproteins including apoA-I, apoB, and apoC-III
- ANGPTL 3/8 complex

9.7. Genetics

A blood sample will be collected for pharmacogenetic analysis as specified in the Schedule of Activities (Section 2), where local regulations allow.

Samples will not be used to conduct unspecified disease or population genetic research either now or in the future. Samples will be used to investigate variable exposure or response to LY3475766. Assessment of variable response may include evaluation of AEs or differences in efficacy.

All samples will be coded with the subject number. These samples and any data generated can be linked back to the subject only by the investigative site personnel.

Samples will be retained for a maximum of 15 years after the last subject visit, or for a shorter period if local regulations and/or ERBs impose shorter time limits, for the study at a facility selected by Lilly or its designee. This retention period enables use of new technologies, response to regulatory questions, and investigation of variable response that may not be observed until later in the development of LY3475766 or after LY3475766 is commercially available.

Molecular technologies are expected to improve during the 15-year storage period and therefore cannot be specifically named. However, existing approaches include whole genome or exome sequencing, genome-wide association studies, multiplex assays, and candidate gene studies. Regardless of technology utilized, data generated will be used only for the specific research scope described in this section.

9.8. Biomarkers

Biomarker research is performed to address questions of relevance to drug disposition, target engagement, PD, mechanism of action, variability of subject response (including safety), and clinical outcome. Sample collection is incorporated into clinical studies to enable examination of these questions through measurement of biomolecules including DNA, RNA, proteins, lipids, and other cellular elements. Nuclear magnetic resonance-determined lipoprotein subclasses and HDL-mediated cholesterol efflux may be evaluated on stored serum samples if deemed necessary by the study team.

Blood samples for non-pharmacogenetic biomarker research will be collected at the times specified in the Schedule of Activities (Section 2) where local regulations allow.

Samples will be used for research on the drug target, disease process, variable response to LY3475766, pathways associated with lipid metabolism, mechanism of action of LY3475766, and/or research method, or for validating diagnostic tools or assay(s) related to lipid metabolism.

All samples will be coded with the subject number. These samples and any data generated can be linked back to the subject only by the investigative site personnel.

Samples will be retained for a maximum of 15 years after the last subject visit, or for a shorter period if local regulations and/or Independent Review Boards (IRBs) impose shorter time limits, at a facility selected by Lilly or its designee. This retention period enables use of new technologies, response to regulatory questions, and investigation of variable response that may not be observed until later in the development of LY3475766 or after LY3475766 is commercially available.

9.9. Health Economics

This section is not applicable for this study.

10. Statistical Considerations and Data Analysis

10.1. Sample Size Determination

The sample size is customary for Phase 1 studies evaluating safety and PK, and is not powered on the basis of statistical hypothesis testing.

Subjects who are randomized and who are discontinued from the study (providing that discontinuation was not as a result of a safety finding) may be replaced to ensure that enough subjects complete the study.

10.2. Populations for Analyses

10.2.1. Study Participant Disposition

A detailed description of subject disposition will be provided at the end of the study.

All subjects who discontinue from the study will be identified, and the extent of their participation in the study will be reported. If known, a reason for their discontinuation will be given. A disposition table for all enrolled subjects will be provided.

10.2.2. Study Participant Characteristics

The subject's age, sex, weight, BMI, height, race, and other demographic data will be recorded and summarized using descriptive statistics.

10.3. Statistical Analyses

Statistical analysis of this study will be the responsibility of Eli Lilly and Company or its designee.

Pharmacokinetic and PD analyses will be conducted on data from all subjects who receive at least 1 dose of the investigational product and have evaluable PK and PD data.

Safety analyses will be conducted for all enrolled subjects who took at least 1 dose of the study medication, whether or not they complete all protocol requirements.

Additional exploratory analyses of the data will be conducted as deemed appropriate. Study results may be pooled with the results of other studies for population PK analysis purposes to avoid issues with post hoc analyses and incomplete disclosures of analyses.

10.3.1. Safety Analyses

10.3.1.1. Clinical Evaluation of Safety

All investigational product and protocol-procedure AEs will be listed, and if the frequency of events allows, safety data will be summarized using descriptive methodology.

The incidence of symptoms for each treatment will be presented by severity and by association with investigational product as perceived by the investigator. Symptoms reported to occur prior to the first dose will be distinguished from those reported as new or increased in severity during

the study. Each symptom will be classified by the most suitable term from the Medical Dictionary for Regulatory Activities.

The number of investigational product-related SAEs will be reported.

10.3.1.2. Statistical Evaluation of Safety

Safety parameters that will be assessed include safety laboratory parameters, vital signs, and ECG parameters. The parameters will be listed and summarized using standard descriptive statistics.

The relationship between systolic BP, diastolic BP, and heart rate from AOBPM and the PK concentration will be evaluated through a longitudinal linear model with independent variables of PK concentration as a continuous variable and time relative to the first dose time as a categorical variable. A compound-symmetry variance-covariance structure will be used to model the within-subject correlation.

The mean systolic BP, diastolic BP, and heart rate by dose will be plotted against time. The maximum value (for each subject) within the 9 weeks after the first dose will be summarized and compared between dose groups using a linear model with dose as a factor and baseline mean value of the corresponding analysis variable as a covariate.

Additional analysis will be performed if warranted upon review of the data.

Baseline for safety parameters will be defined as the last evaluable value before the first dose.

10.3.2. Pharmacokinetic Analyses

10.3.2.1. Pharmacokinetic Parameter Estimation

Pharmacokinetic parameter estimates for LY3475766 will be calculated using standard noncompartmental methods of analysis.

The primary parameters for analysis will be AUC from time 0 to infinity (AUC[0-∞]), AUC from time 0 to the time of the last quantifiable concentration (AUC[0-t_{last}]), C_{max}, and time to reach C_{max} (t_{max}). Additionally, the bioavailability (F) following SC dose will be assessed. Other noncompartmental parameters, such as half-life, apparent clearance, and apparent volume of distribution, may be reported. If deemed necessary, additional model-based analysis may be performed.

10.3.2.2. Pharmacokinetic Statistical Inference

The descriptive statistics (geometric mean and coefficient of variation) for the PK parameters will be provided for each dose level. Log-transformation will be applied to C_{max} and AUC in calculation of geometric mean and coefficient of variation.

The dose proportionality for LY3475766 will be assessed for AUC and C_{max} using a power model. The power parameter will be evaluated to determine the dose proportionality.

10.3.3. Pharmacodynamic Analyses

10.3.3.1. Pharmacodynamic Parameter Estimation

The primary parameters for this analysis will be fasting TG levels, fasting LDL-C levels, other lipid biomarkers, and fasting ANGPTL 3/8 complex levels. Pharmacodynamic data will be summarized as reported values and as changes and percentage changes from baseline using descriptive statistics.

10.3.3.2. Pharmacodynamic Statistical Inference

The actual value, change from baseline, and percentage change from baseline for TG, LDL-C, and other PD parameters will be summarized for each dose level. Exploratory dose-response analysis may be performed as necessary. Change from baseline or percent change from baseline for PD parameters (TG, LDL, apoB, and ANGPTL3/8) will be analyzed by a longitudinal linear model. A log-transformation may be applied if the distribution of the variable is considered skewed.

10.3.4. Pharmacokinetic/Pharmacodynamic Analyses

Exploratory graphical PK/PD analyses relating serum exposure of LY3475766 to TG, LDL-C, and apoB reduction will be conducted. Additionally, exploratory graphical PK/PD analyses relating serum exposure of LY3475766 to the changes to the other PD endpoints (total cholesterol, non-HDL, VLDL-C, HDL-C, apoA-1, apoC-III, and ANGPTL 3/8 complex) may be conducted. Pharmacokinetic/PD models exposure-response may be explored to characterize the relationship of LY3475766 concentrations and the time course of reduction in TG and LDL-C.

10.3.5. Evaluation of Immunogenicity

Upon full assay validation, TE-ADAs will be assessed. The frequency and percentage of subjects with preexisting ADAs and with TE-ADAs to LY3475766 may be tabulated.

Treatment-emergent ADAs are defined as those with a titer 2-fold (1 dilution) greater than the minimum required dilution if no ADAs were detected at baseline (treatment-induced ADA) or those with a 4-fold (2 dilutions) increase in titer compared to baseline if ADAs were detected at baseline (treatment-boosted ADA).

The frequency of neutralizing antibodies may also be tabulated in TE-ADA+ subjects, when available.

Additional clinical analyses of ADA results will be performed, as appropriate, to assess the relationship between the presence of antibodies and the PK, efficacy, and safety parameters as recommended in Shankar et al. 2014.

10.3.6. Data Review during the Study

Interim access to available safety, PK, and PD data are planned for this study. The purpose of these reviews is to enable dose escalation, to guide dose selection for subsequent cohorts, and to inform the design of subsequent studies. The investigator (blinded) and the Lilly sponsor team (unblinded) will make decisions regarding dose escalation based upon their data review.

Access to safety data will occur when at least 7 subjects have completed study activities through

- Day 8 in Cohorts 1, 2, and 6, and
- Day 15 for Cohorts 3, 4, and 5.

Future Studies

To influence the design of future studies, a review of all available safety and tolerability, PK, and PD data will occur. The review will include

- any available data from Cohorts 1 to 5, and
- any available data from subjects in Cohort 6 who have completed all requirements in the Schedule of Activities (Section 2) up to Day 15.

10.3.7. Interim Analyses

No interim analyses are planned for this study. If an unplanned interim analysis is deemed necessary, the Lilly CP, CRP/investigator, or designee will consult with the appropriate medical director or designee to determine if it is necessary to amend the protocol.

11. References

[EMA] European Medicines Agency. EMEA/CHMP/SWP/289367/07 Rev. 1. Guideline on strategies to identify and mitigate risks for first-in-human and early clinical trials with investigational medicinal products. Available at: https://www.ema.europa.eu/en/strategies-identify-mitigate-risks-first-human-early-clinical-trials-investigational-medicinal. Published 20 July 2017. Accessed June 17, 2019.

Ganda OP, Bhatt DL, Mason RP, Miller M, Boden WE. Unmet need for adjunctive dyslipidemia therapy in hypertriglyceridemia management. *J Am Coll Cardiol*. 2018;72(3):330-343.

Shankar G, Arkin S, Cocea L, Devanarayan V, Kirshner S, Kromminga A, Quarmby V, Richards S, Schneider CK, Subramanyam M, Swanson S, Verthelyi D, Yim S, American Association of Pharmaceutical Scientists. Assessment and reporting of the clinical immunogenicity of therapeutic proteins and peptides—harmonized terminology and tactical recommendations. *AAPS J*. 2014;16(4):658-673.

12. Appendices

Appendix 1. Abbreviations and Definitions

Term	Definition
**ADA**	anti-drug antibody
**AE**	adverse event: Any untoward medical occurrence in a patient or clinical investigation subject administered a pharmaceutical product that does not necessarily have a causal relationship with this treatment. An AE can therefore be any unfavorable and unintended sign (including an abnormal laboratory finding), symptom, or disease temporally associated with the use of a medicinal (investigational) product, whether or not related to the medicinal (investigational) product.
**ALP**	alkaline phosphatase
**ALT**	alanine aminotransferase
**ANGPTL**	angiopoietin-like protein
**AOBPM**	automated office blood pressure monitoring
**apo**	apolipoprotein
**ASCVD**	atherosclerotic cardiovascular disease
**AST**	aspartate aminotransferase
**AUC**	area under the concentration versus time curve
$\mathbf{AUC_{(0-\infty)}}$	area under the concentration versus time curve from time 0 to infinity
$\mathbf{AUC_{(0-t_{last})}}$	area under the concentration versus time curve from time 0 to the time of the last quantifiable concentration
**blinding**	A procedure in which one or more parties to the study are kept unaware of the treatment assignment(s). Unless otherwise specified, blinding will remain in effect until final database lock.<br><br>A single-blind study is one in which the investigator and/or his or her staff are aware of the treatment but the subject is not, or vice versa, or when the sponsor is aware of the treatment but the investigator and/his or staff and the subject are not. A double-blind study is one in which neither the subject nor any of the investigator or sponsor staff who are involved in the treatment or clinical evaluation of the subjects are aware of the treatment received
**BMI**	body mass index
**BP**	blood pressure
$\mathbf{C_{max}}$	maximum observed concentration

**complaint**	A complaint is any written, electronic, or oral communication that alleges deficiencies related to the identity, quality, purity, durability, reliability, safety or effectiveness, or performance of a drug or drug delivery system.
**compliance**	Adherence to all the study-related requirements, good clinical practice (GCP) requirements, and the applicable regulatory requirements.
**confirmation**	A process used to confirm that laboratory test results meet the quality requirements defined by the laboratory generating the data and that Lilly is confident that results are accurate. Confirmation will either occur immediately after initial testing or will require that samples be held to be retested at some defined time point, depending on the steps required to obtain confirmed results.
**CP**	clinical pharmacologist
**CRF**	case report form
**CRP**	clinical research physician: Individual responsible for the medical conduct of the study. Responsibilities of the CRP may be performed by a physician, clinical research scientist, global safety physician, or other medical officer.
**CRU**	clinical research unit
**CV**	cardiovascular
**ECG**	electrocardiogram
**eCRF**	electronic case report form
**EMA**	European Medicines Agency
**enroll**	The act of assigning a subject to a treatment. Subjects who are enrolled in the study are those who have been assigned to a treatment.
**enter**	Subjects entered into a study are those who sign the informed consent form directly or through their legally acceptable representatives.
**ERB**	ethical review board
**F**	bioavailability
**GCP**	good clinical practice
**HCV**	hepatitis C virus
**HDL-C**	high-density lipoprotein cholesterol
**HIV**	human immunodeficiency virus
**IB**	Investigator's Brochure
**ICF**	informed consent form
**ICH**	International Council for Harmonisation

**Ig**	immunoglobulin
**informed consent**	A process by which a subject voluntarily confirms his or her willingness to participate in a particular study, after having been informed of all aspects of the study that are relevant to the subject's decision to participate. Informed consent is documented by means of a written, signed, and dated informed consent form.
**interim analysis**	An interim analysis is an analysis of clinical study data, separated into treatment groups, that is conducted before the final reporting database is created/locked.
**investigational product (IP)**	A pharmaceutical form of an active ingredient or placebo being tested or used as a reference in a clinical study, including products already on the market when used or assembled (formulated or packaged) in a way different from the authorized form, or marketed products used for an unauthorized indication, or marketed products used to gain further information about the authorized form.
**investigator**	A person responsible for the conduct of the clinical study at a study site. If a study is conducted by a team of individuals at a study site, the investigator is the responsible leader of the team and may be called the principal investigator.
**IRB**	Independent Review Board
**ISR**	injection-site reaction
**IV**	intravenous; intravenously
**LDL-C**	low-density lipoprotein cholesterol
**legal representative**	An individual or judicial or other body authorized under applicable law to consent, on behalf of a prospective subject, to the subject's participation in the clinical study.
**MABEL**	minimal anticipated biological effect level
**MTD**	maximum tolerated dose
**NOAEL**	no-observed-adverse-effect level
**PCSK9**	proprotein convertase subtilisin/kexin type 9
**PD**	pharmacodynamic(s)
**PK**	pharmacokinetic(s)
**QTc**	QT interval corrected for heart rate
**randomize**	the process of assigning subjects/patients to an experimental group on a random basis
**SAD**	single-ascending dose
**SAE**	serious adverse event
**SC**	subcutaneous; subcutaneously

**screen**	The act of determining if an individual meets minimum requirements to become part of a pool of potential candidates for participation in a clinical study.
**SUSAR**	suspected unexpected serious adverse reaction
**TBL**	total bilirubin level
**TE-ADA**	treatment-emergent anti-drug antibody
**TG**	triglyceride(s)
**t_{max}**	time to maximum observed drug concentration
**ULN**	upper limit of normal
**VLDL-C**	very low-density lipoprotein cholesterol

Appendix 2. Clinical Laboratory Tests

Screening and Safety Laboratory Tests

**Hematology**	**Clinical Chemistry**
Hematocrit	Sodium
Hemoglobin	Potassium
Erythrocyte count (RBC)	Bicarbonate
Mean cell volume	Chloride
Mean cell hemoglobin	Calcium
Mean cell hemoglobin concentration	Phosphorus
Leukocytes (WBC)	Magnesium
Platelets	
	Triglycerides[a]
Absolute counts of:	Total cholesterol
Neutrophils	LDL-C[a, g]
Lymphocytes	Amylase
Monocytes	Lipase
Eosinophils	Fasting glucose
Basophils	Blood urea nitrogen (BUN)
	Creatinine
**Urinalysis**	CPK
Specific gravity[b]	Uric acid
pH[b]	
Protein[b, e]	Total protein
Glucose[b]	Albumin
Ketones[b]	Total bilirubin
Bilirubin[b]	Alkaline phosphatase (ALP)
Urobilinogen[b]	Aspartate aminotransferase (AST)
Blood[b, e]	Alanine aminotransferase (ALT)
Nitrite[b, e]	Gamma-glutamyl transferase (GGT)
	**Serology**
	Hepatitis B surface antigen[b]
	Hepatitis B core antibody[b]
	Hepatitis B surface antibody[b]
	Hepatitis C antibody[b,c]
	Hepatitis C virus (HCV) ribonucleic acid (RNA)[b,c]
	HIV[b]
	**Other**
	Ethanol testing[a]
	Urine drug screen[a]
	Pregnancy test[d] (serum[b] and urine[f])
	FSH[b,d]
	Thyroid-stimulating hormone[b]
	HbA1c[b]

Abbreviations: CPK = creatinine phosphokinase; FSH = follicle-stimulating hormone; HbA1c = hemoglobin A1c; HIV = human immunodeficiency virus; LDL-C = low-density lipoprotein cholesterol; RBC = red blood cell; TG = triglyceride; WBC = white blood cell.

[a] Performed at screening and Day -1.
[b] Performed at screening only.
[c] A positive hepatitis C antibody laboratory assessment will be confirmed with a test for HCV RNA.
[d] For female subjects only.
[e] Perform microscopic reflex if positive.
[f] Perform as indicated in Section 2.
[g] Since LDL-C is largely underestimated by the Friedewald formula (or Martin-Hopkins calculation) at high TG levels, it will be directly measured when TG levels are >400 mg/dL.

Appendix 3. Study Governance, Regulatory, and Ethical Considerations

Informed Consent

The investigator is responsible for:

- ensuring that the subject understands the nature of the study, the potential risks and benefits of participating in the study, and that their participation is voluntary.
- ensuring that informed consent is given by each subject or legal representative. This includes obtaining the appropriate signatures and dates on the informed consent form (ICF) prior to the performance of any protocol procedures and prior to the administration of investigational product.
- answering any questions the subject may have throughout the study and sharing in a timely manner any new information that may be relevant to the subject's willingness to continue his or her participation in the study.
- providing a copy of the ICF to the participant or the participant's legal representative and retaining a copy on file.

Recruitment

Lilly or its designee is responsible for the central recruitment strategy for patients. Individual investigators may have additional local requirements or processes. Study-specific recruitment material should be approved by Lilly.

Ethical Review

The investigator or appropriate local representative must give assurance that the ethical review board (ERB) was properly constituted and convened as required by International Council for Harmonisation (ICH) guidelines and other applicable laws and regulations.

Documentation of ERB approval of the protocol and the ICF must be provided to Lilly before the study may begin at the investigative site(s). Lilly or its representatives must approve the ICF before it is used at the investigative site(s). All ICFs must be compliant with the ICH guideline on good clinical practice (GCP).

The study site's ERB(s) should be provided with the following:

- the current Investigator's Brochure (IB) and updates during the course of the study
- ICF
- relevant curricula vitae

Regulatory Considerations

This study will be conducted in accordance with the protocol and with:

1) consensus ethics principles derived from international ethics guidelines, including the Declaration of Helsinki and Council for International Organizations of Medical Sciences International Ethical Guidelines
2) applicable ICH GCP Guidelines
3) applicable laws and regulations

Some of the obligations of the sponsor will be assigned to a third-party organization.

Protocol Signatures

The sponsor's responsible medical officer will approve the protocol, confirming that, to the best of his or her knowledge, the protocol accurately describes the planned design and conduct of the study.

After reading the protocol, each principal investigator will sign the protocol signature page and send a copy of the signed page to a Lilly representative.

Final Report Signature

The final report coordinating investigator or designee will sign the clinical study report for this study, indicating agreement that, to the best of his or her knowledge, the report accurately describes the conduct and results of the study.

The investigator with the most qualified, analyzable, and enrolled subjects will serve as the final report coordinating investigator. If this investigator is unable to fulfill this function, another investigator will be chosen by Lilly to serve as the final report coordinating investigator.

The sponsor's responsible medical officer and statistician will sign and approve the final clinical study report for this study, confirming that, to the best of his or her knowledge, the report accurately describes the conduct and results of the study.

Data Quality Assurance

To ensure accurate, complete, and reliable data, Lilly or its representatives will do the following:

- provide instructional material to the study sites, as appropriate.
- provide training to instruct the investigators and study coordinators. This training will give instruction on the protocol, the completion of the case report forms (CRFs), and study procedures.
- make periodic visits to the study site.
- be available for consultation and stay in contact with the study site personnel by mail, telephone, and/or fax.

- review and evaluate CRF data and/or use standard computer edits to detect errors in data collection.
- conduct a quality review of the database.

In addition, Lilly or its representatives will periodically check a sample of the subject data recorded against source documents at the study site. The study may be audited by Lilly and/or regulatory agencies at any time. Investigators will be given notice before an audit occurs.

The investigator will keep records of all original source data. This might include laboratory tests, medical records, and clinical notes. If requested, the investigator will provide the sponsor, applicable regulatory agencies, and applicable ERBs with direct access to the original source documents.

Data Collection Tools/Source Data

An electronic data capture system will be used in this study. The site must define and retain all source records and must maintain a record of any data where source data are directly entered into the data capture system.

Data Protection

Data systems used for the study will have controls and requirements in accordance with local data protection law.

The purpose and use of subject personal information collected will be provided in a written document to the subject by the sponsor.

Study and Site Closure

Discontinuation of Study Sites

Study site participation may be discontinued if Lilly or its designee, the investigator, or the ERB of the study site judges it necessary for medical, safety, regulatory, or other reasons consistent with applicable laws, regulations, and GCP.

Discontinuation of the Study

The study will be discontinued if Lilly or its designee judges it necessary for medical, safety, regulatory, or other reasons consistent with applicable laws, regulations, and GCP.

Appendix 4. Hepatic Monitoring Tests for Treatment-Emergent Abnormality

Selected tests may be obtained in the event of a treatment-emergent hepatic abnormality and may be required in follow-up with patients in consultation with Lilly or its designee clinical research physician.

Hepatic Monitoring Tests

**Hepatic Hematology**[a]	**Haptoglobin**[a]
Hemoglobin	
Hematocrit	**Hepatic Coagulation**[a]
RBC	Prothrombin time
WBC	Prothrombin time, INR
Neutrophils	
Lymphocytes	**Hepatic Serologies**[a,b]
Monocytes	Hepatitis A antibody, total
Eosinophils	Hepatitis A antibody, IgM
Basophils	Hepatitis B surface antigen
Platelets	Hepatitis B surface antibody
	Hepatitis B core antibody
**Hepatic Chemistry**[a]	Hepatitis C antibody
Total bilirubin	Hepatitis E antibody, IgG
Conjugated bilirubin	Hepatitis E antibody, IgM
Alkaline phosphatase	
ALT	**Anti-nuclear antibody**[a]
AST	**Alkaline phosphatase isoenzymes**[a]
GGT	**Anti-smooth muscle antibody (or anti-actin antibody)**[a]
CPK	

Abbreviations: ALT = alanine aminotransferase; AST = aspartate aminotransferase; CPK = creatinine phosphokinase; GGT = gamma-glutamyl transferase; Ig = immunoglobulin; INR = international normalized ratio; RBC = red blood cell; WBC = white blood cell.

a Assayed by Lilly-designated or local laboratory.

b Reflex/confirmation dependent on regulatory requirements and/or testing availability.

Appendix 5. Blood Sampling Summary

The following tables summarize the approximate number of venipunctures and blood volumes for all blood sampling (screening, safety laboratories, and bioanalytical assays) during the study.

Protocol J1T-MC-GZEA Sampling Summary

		Number of Blood Samples		Total Volume (mL)	
**Purpose**	**Blood Volume per Sample (mL)**	**IV Cohorts**	**SC Cohorts**	**IV Cohorts**	**SC Cohorts**
Screening tests[a]	20.5	1	1	20.5	20.5
Clinical laboratory tests[a]	8	9	9	72	72
Pharmacokinetics	3	10	9	30	27
Blood discard for cannula patency	1	1	0	1	0
Pharmacodynamics					
ApoA-I, apoB, apoC-III	4	11	11	44	44
Lipid panel[b]	4	11	11	44	44
ANGPTL 3/8 complex	2.5	9	8	22.5	20
Immunogenicity	10	4	4	40	40
Pharmacogenetics	10	1	1	10	10
Stored serum samples	6	6	6	36	36
Total				320	313.5
Total for clinical purposes				320	320

Abbreviations: ANGPTL = angiopoietin-like protein; apo = apolipoprotein; IV = intravenous; HDL-C = high-density lipoprotein cholesterol; LDL-C = low-density lipoprotein cholesterol; SC = subcutaneous; VLDL-C = very-low-density lipoprotein cholesterol.

a Additional samples may be drawn if needed for safety purposes.

b The lipid panel includes triglycerides, total cholesterol, LDL-C, VLDL-C (calculated), HDL-C, and non-HDL-C (calculated).

Appendix 6. Protocol Amendment J1T-MC-GZEA(d) Summary

A Randomized, Double-Blind, Placebo-Controlled Study to Evaluate the Safety, Tolerability, Pharmacokinetics, and Pharmacodynamics of LY3475766 Overview

Protocol J1T-MC-GZEA, A Randomized, Double-Blind, Placebo-Controlled Study to Evaluate the Safety, Tolerability, Pharmacokinetics, and Pharmacodynamics of LY3475766, has been amended. The new protocol is indicated by Amendment (d) and will be used to conduct the study in place of any preceding version of the protocol.

This amendment is considered a substantial protocol amendment

The present protocol amendment is intended to change the route of administration in Cohort 5 from IV to SC. The rationale for this change is to enable a better characterization of the duration of pharmacodynamic effects on lipid parameters. The Cohort 5 dose is limited to 600 mg because it requires 6 SC injections given the drug product concentration of 50 mg/mL.

In addition, the maximal number of subjects that can be enrolled in the study was increased from 55 to 70 (Section 5.2) to account for the

1. fact that Cohort 4 needs to be repeated because limited follow-up data are available due to the absence of site visit during multiple weeks around the peak of the COVID-19 pandemic, and
2. potential need to replace subjects from future cohorts in case they are unable to attend site visits due to COVID-19.

The change to performing the complete physical examination either at the screening visit or at check-in/Day -1 was to provide allowance for COVID-19 mitigations depending on local guidance at each CRU.

Finally, changes were made to allow for the possibility of home visits to capture safety and pharmacodynamic parameters in case subjects cannot come to the sites for 1 or several visits, given the pandemic context.

Revised Protocol Sections

**Note:**	All deletions have been identified by ~~strikethroughs~~.<br>All additions have been identified by the use of underscore.

1. Protocol Synopsis

Number of Subjects:

Up to ~~55~~70 subjects may be enrolled so that 48 subjects (LY3475766: 36, Placebo: 12) have sufficient evaluable data.

2. Schedule of Activities

Study Schedule Protocol J1T-MC-GZEA

Procedure	Screening		Treatment Period													Follow-up	ET	Notes
			Week 1							Week 2	Week 3	Week 4	Week 5	Week 7	Week 9	Week 13		
**Day**	**Day -28 to Day -2**	**-1**	**1**	**2**	**3**	**4**	**5**	**6**	**7**	**8**	**15 ±2**	**22 ±2**	**29 ±2**	**43 ±5**	**57 ±5**	**85 ±5**		
Complete physical examination	X																	Complete examination (except genital and rectal). Can be completed at either Screening or Day -1.

3.3 Benefit/Risk Assessment

LY3475766 has not been administered to humans. The nonclinical safety information for LY3475766 supports the transition from preclinical status to clinical development. Based on the mechanism of action and nonclinical data, LY3475766 is not considered to be a high uncertainty compound. The only potential risk that is anticipated relates to the finding of a nondose-dependent decrease in heart rate in a 1-month Good Laboratory Practice toxicology study that was performed in cynomolgus monkeys; there were no adverse events (AEs) or any significant electrocardiogram (ECG) findings associated with the decrease in heart rate. Refer to the IB for LY3475766 for more details. This risk is considered to be monitorable and manageable at the planned doses of 10 mg, 30 mg, ~~450 mg,~~ and 1000 mg IV and 100 mg, 300 mg, and 600 mg SC for LY3475766 in dyslipidemic but otherwise healthy subjects.

5.1. Overall Design

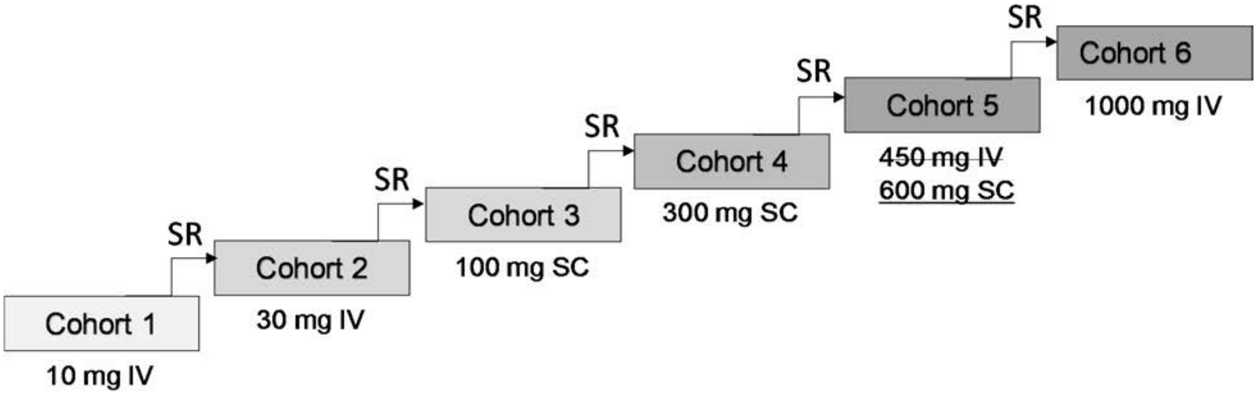

Figure GZEA.1. Dose escalation for Protocol J1T-MC-GZEA

Potential subjects will be screened to assess their eligibility to enter the study within 28 days prior to Day 1. In case of safety concerns, participants may be required to stay at the clinical site for a longer period at the discretion of the investigator. Subjects in the IV cohorts will be confined at the CRU from Day -1 (the day before dosing) to Day 4 (72 hours postdose) but will return to the CRU on Day 8 for an outpatient visit. Subjects in the SC cohorts will be confined at the CRU from Day -1 (the day before dosing) to Day 8 (168 hours postdose). Subjects in all cohorts will return to the clinical research unit (CRU) as outpatients at predetermined visits for up to approximately 84 days postdose for follow-up assessments. In case a subject cannot come to the site for 1 or several outpatient visits, home visits may potentially be performed instead, depending on the circumstances and if agreed upon between investigator and sponsor.

Subjects will be confined at the CRU:

- from Day -1 (the day before dosing) to Day 4 (72 hours postdose) for IV cohorts, and
- from Day -1 (the day before dosing) to Day 8 (168 hours postdose) for SC cohorts.

This is a randomized double-blinded SAD study in dyslipidemic but otherwise healthy male and female subjects investigating the safety, tolerability, PD, and PK of LY3475766 versus placebo.

Subjects will be enrolled in 6 cohorts:

- Cohort 1: 10 mg LY3475766 or placebo IV
- Cohort 2: 30 mg LY3475766 or placebo IV
- Cohort 3: 100 mg LY3475766 or placebo SC
- Cohort 4: 300 mg LY3475766 or placebo SC
- Cohort 5: 600 mg LY3475766 or placebo ~~IV~~ SC
- Cohort 6: 1000 mg LY3475766 or placebo IV

~~Potential subjects will be screened to assess their eligibility to enter the study within 28 days prior to Day 1. In case of safety concerns, participants may be required to stay at the clinical site for a longer period at the discretion of the investigator. Subjects in the IV cohorts will be confined at the CRU from Day -1 (the day before dosing) to Day 4 (72 hours postdose) but will return to the CRU on Day 8 for an outpatient visit. Subjects in the SC cohorts will be confined at the CRU from Day -1 (the day before dosing) to Day 8 (168 hours postdose). Subjects in all cohorts will return to the clinical research unit (CRU) as outpatients at predetermined visits for up to approximately 84 days postdose for follow-up assessments~~.

5.2. Number of Participants

Up to ~~55~~70 subjects may be enrolled so that 48 subjects (LY3475766: 36, Placebo: 12) have sufficient evaluable data.

5.5. Justification of Dose

Doses of 10, 30~~, 450~~ and 1000 mg IV and 100, 300, and 600 mg SC LY3475766 were selected based on preclinical pharmacology and toxicology data.

6.1. Inclusion Criteria

[1b] female subjects must be of nonchildbearing potential and include those who are infertile due to surgical sterilization (such as hysterectomy, bilateral salpingectomy, bilateral tubal ligation, or bilateral oophorectomy with verbal confirmation from the subject), congenital anomaly such as Mullerian agenesis, or those who are postmenopausal, defined as:

i. a woman at least ~~50~~ 40 years of age with an intact uterus, not on hormone replacement therapy, who has had cessation of menses for at least 1 year or at least 6 months of spontaneous amenorrhea with a follicle-stimulating hormone level >40 mIU/mL; or

6.3.2. Caffeine, Alcohol and Tobacco

While confined at the CRU, subjects are required to adhere to the CRU smoking and caffeine policy during the inpatient treatment days. Subjects should otherwise not intentionally change their consumption of tobacco- or caffeine-containing products during the study.

No alcohol will be allowed during the 24 hours before and 72 hours postdose, and 24 hours before each CRU admission and each outpatient visit, and throughout the duration of each CRU visit. Between ~~CRU~~ visits, daily alcohol consumption should not exceed 2 units for males and 1 unit for females (a unit is defined in Exclusion Criterion [34], Section

7.1. Treatment Administered

The investigational product will be administered either via an SC injection or as a slow IV infusion (over at least 30 minutes for Cohorts 1 and 2, or at least 60 minutes for Cohort~~s 5 and~~ 6). For SC administration, LY3475766 and placebo will be administered at a maximum volume

of 2 mL per injection. A maximum of 46 injections may be necessary to achieve higher planned dose levels.

Table GZEA.5. Treatments Administered Subcutaneously

Dose Level	LY Concentration per Vial (mg/mL)	Number of Vials	Volume per Injection (mL)	Number of Injections	Total Volume of Injection (mL)
LY 100 mg/PL	50	1.3	2.0	1	2
LY 300 mg/PL	50	45	2.0	3	6
LY 600 mg/PL	50	10	2.0	6	12

Abbreviations: LY = LY3475766; PL = placebo.

Injection site selected for SC administration should be the abdominal region approximately 5 cm from the umbilicus and the treatment has to be administered through the needle applied at approximately 45° with pinching of the skin. Because subjects may receive multiple injections per dose, each injection should be administered in a different abdominal quadrant in Cohorts 3 and 4 (rotating from right upper quadrant, to right lower quadrant, to left lower quadrant, to left upper quadrant), the quadrant used should be recorded at the time of the injection. For Cohort 5 where 6 SC injections are necessary, the injections should be administered into 6 separate regions of the abdomen: upper right, middle right (same level as umbilicus), lower right, lower left, middle left (same level as umbilicus), and upper left. Administering in a clockwise or counterclockwise direction should be considered to avoid injecting into the same location twice. The abdominal location for each injection should be recorded at the time of the injection. All injections should be performed within a total of 510 minutes. Subcutaneous administration of LY3475766 should be done by a limited number of individuals for consistency. The same type of syringe and needle should be used for all subjects to ensure all injections are delivered to a consistent depth target into the SC space.

7.4.1 Dose Escalation

Dose-escalation decisions will primarily be based on available safety and tolerability data obtained up to Day 8 from Cohorts 1, 2, 5and 6 and up to Day 15 from Cohorts 3, 4, and 5 (Section 10.3.6). Additionally, any available PK data may be used to guide dose selection or to determine if the number of doses to be studied may be reduced.

In addition to safety reasons, the sponsor may decide to stop further dose escalation if pharmacodynamic data obtained from the previous cohorts are considered sufficient.

10.3.6. Data Review during the Study

Access to safety data will occur when at least 7 subjects have completed study activities through

- Day 8 in Cohorts 1, 2, ~~5~~ and 6, and
- Day 15 for Cohorts 3, 4, and 5.

Leo Document ID = 3dd20392-1052-416e-8870-e6f45180bafe

PPD

Approval Date & Time: 26-Aug-2020 12:14:59 GMT

Signature meaning: Approved

PPD

Approval Date & Time: 27-Aug-2020 17:00:10 GMT

Signature meaning: Approved

STATISTICAL ANALYSIS PLAN

A Randomized, Double-Blind, Placebo-Controlled Study to Evaluate the Safety, Tolerability, Pharmacokinetics, and Pharmacodynamics of LY3475766

Statistical Analysis Plan Status: Final V3
Statistical Analysis Plan Date: 19-April-2021

Study Drug: LY3475766

Sponsor Reference: J1T-MC-GZEA
Covance CRU Study: 1000071-8409160

Clinical Phase I

Approval Date: 23-Apr-2021 GMT

1. TABLE OF CONTENTS

1. TABLE OF CONTENTS 2
2. ABBREVIATIONS 3
3. INTRODUCTION 4
4. STUDY OBJECTIVES 4
4.1 Primary Objectives 4
4.2 Secondary Objectives 4
4.3 Exploratory Objectives 5
5. STUDY DESIGN 5
6. TREATMENTS 5
7. SAMPLE SIZE JUSTIFICATION 6
8. DEFINITION OF ANALYSIS POPULATIONS 6
9. STATISTICAL METHODOLOGY 6
9.1 General 6
9.2 Demographics and Subject Disposition 7
9.3 Pharmacokinetic Assessment 7
9.3.1 Pharmacokinetic Analysis 7
9.3.2 Pharmacokinetic Statistical Methodology 11
9.4 Pharmacodynamic Assessment 12
9.4.1 Pharmacodynamic Analysis 12
9.5 Safety and Tolerability Assessments 13
9.5.1 Adverse events 13
9.5.2 Concomitant medication 13
9.5.3 Clinical laboratory parameters 13
9.5.4 Vital signs 13
9.5.5 Electrocardiogram (ECG) 15
9.5.6 Hepatic Monitoring 15
9.5.7 Immunogenicity Assessments 15
9.5.8 Injection-Site Reactions 15
9.5.9 Other assessments 16
10. INTERIM ANALYSES 16
11. CHANGES FROM THE PROTOCOL SPECIFIED STATISTICAL ANALYSES 16
12. REFERENCES 16
13. DATA PRESENTATION 16
13.1 Derived Parameters 16
13.2 Missing Data 16
13.3 Insufficient Data for Presentation 16

2. ABBREVIATIONS

Abbreviations pertain to the Statistical Analysis Plan (SAP) only (not the tables, figures and listings [TFLs]).

AE	Adverse event
AOBPM	Automated office blood pressure monitoring data
AUC	Area under the concentration versus time curve
AUC(0-t_{last})	Area under the concentration versus time curve from time zero to time t, where t is the last time point with a measurable concentration
AUC(0-∞)	Area under the concentration versus time curve from time zero to infinity
BQL	Below the lower limit of quantification
C_{max}	Maximum observed drug concentration
CI	Confidence interval
CRU	Clinical Research Unit
CSR	Clinical Study Report
ECG	Electrocardiogram
e.g.	For example (Latin: *exempli gratia*)
ICH	International Conference on Harmonisation
IV	Intravenous
LDL-C	Low-density lipoprotein cholesterol
MAD	Multiple-Ascending Dose
PD	Pharmacodynamic
PK	Pharmacokinetic
QTcF	The QT interval corrected using Fridericia's formula
SAD	Single-Ascending Dose
SAP	Statistical Analysis Plan
SC	Subcutaneous
SD	Standard deviation
TFLs	Tables, Figures, and Listings
TG	Triglyceride
$t_{1/2}$	Half-life associated with the terminal rate constant (λ_z) in non-compartmental analysis
t_{max}	Time of maximum observed drug concentration
ULN	Upper limit of normal

3. INTRODUCTION

This SAP has been developed after review of the Clinical Study Protocol (final version dated 27 June 2019), amendment (a) (final version dated 19 September 2019), amendment (b) (final version dated 12 December 2019), amendment (c) (final version dated 14 February 2020), amendment (d) (final version dated 27 August 2020), final SAP version 1 (dated 25 September 2019), and SAP version 2 (dated 04 September 2020).

This SAP describes the planned analysis of the safety, tolerability, pharmacokinetic (PK) and pharmacodynamic (PD) data from this study. A detailed description of the planned TFLs to be presented in the clinical study report (CSR) is provided in the accompanying TFL shell document.

The intent of this document is to provide guidance for the statistical, PK and PD analyses of data. In general, the analyses are based on information from the protocol, unless they have been modified with Eli Lilly and Company. A limited amount of information concerning this study (e.g., objectives, study design) is given to help the reader's interpretation. This SAP must be signed off prior to first subject treatment administration for this study. When the SAP and TFL shells are agreed upon and finalized, they will serve as the template for this study's CSR.

This SAP supersedes the statistical considerations identified in the protocol; where considerations are substantially different, they will be so identified. If additional analyses are required to supplement the planned analyses described in this SAP, they may be performed and will be identified in the CSR. Any substantial deviations from this SAP will be agreed upon with Eli Lilly and Company and identified in the CSR. Any minor deviations from the TFLs may not be documented in the CSR.

This SAP is written with consideration of the recommendations outlined in the International Conference on Harmonisation (ICH) E9 Guideline entitled Guidance for Industry: Statistical Principles for Clinical Trials[1] and the ICH E3 Guideline entitled Guidance for Industry: Structure and Content of Clinical Study Reports[2].

4. STUDY OBJECTIVES

4.1 Primary Objectives

- To assess the safety and tolerability of single intravenous (IV) and subcutaneous (SC) doses of LY3475766 in dyslipidemic but otherwise healthy subjects.

4.2 Secondary Objectives

- To characterize the PK of LY3475766 following single doses in dyslipidemic but otherwise healthy subjects.
- To characterize the PD of LY3475766 following single doses in dyslipidemic but otherwise healthy subjects.

4.3 Exploratory Objectives

- To explore the PD effect of LY3475766 on the lipid profile of single doses in dyslipidemic but otherwise healthy subjects.

5. STUDY DESIGN

This first-in-human study will investigate the safety, tolerability, PK, and PD of LY3475766 in dyslipidemic but otherwise healthy subjects (single-ascending dose [SAD]).

Figure GZEA.1. illustrates the study design.

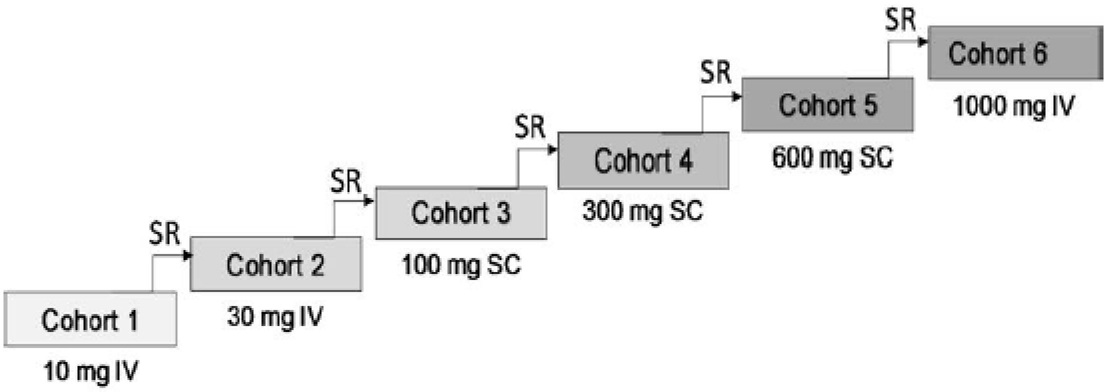

Abbreviations: IV = intravenous; SC = subcutaneous; SR = safety review.

Figure GZEA.1. Dose escalation for Protocol J1T-MC-GZEA.

This is a randomized double-blinded SAD design in dyslipidemic but otherwise healthy male and female subjects, investigating the safety, tolerability, PD, and PK of LY3475766 versus placebo.

Subjects will be enrolled in 6 cohorts:

- Cohort 1: 10 mg LY3475766 or placebo IV
- Cohort 2: 30 mg LY3475766 or placebo IV
- Cohort 3: 100 mg LY3475766 or placebo SC
- Cohort 4: 300 mg LY3475766 or placebo SC
- Cohort 5: 600 mg LY3475766 or placebo SC
- Cohort 6: 1000 mg LY3475766 or placebo IV

Doses and the number of cohorts may be adjusted based on ongoing review of the PK, safety, and tolerability data.

6. TREATMENTS

The following is a list of the study treatment names that will be used in the TFLs.

Study Treatment Name	Treatment order in TFL
Placebo	1
10 mg LY3475766 IV	2
30 mg LY3475766 IV	3
100 mg LY3475766 SC	4
300 mg LY3475766 SC	5
600 mg LY3475766 SC	6
1000 mg LY3475766 IV	7

Placebo data will be pooled. The above dose levels will be updated on the TFLs for the actual dose received.

7. SAMPLE SIZE JUSTIFICATION

Up to 70 subjects may be enrolled so that 48 subjects (LY3475766: 36, Placebo: 12) have sufficient evaluable data.

The sample size is customary for Phase 1 studies evaluating safety and PK, and is not powered on the basis of statistical hypothesis testing.

Subjects who are randomized and who are discontinued from the study (providing that discontinuation was not as a result of a safety finding) may be replaced to ensure that enough subjects complete the study.

8. DEFINITION OF ANALYSIS POPULATIONS

The "Safety" population will consist of all enrolled subjects who took at least 1 dose of the study medication, whether or not they completed all protocol requirements.

The "Pharmacokinetic" population will consist of all subjects who received at least one dose of LY3475766 and have evaluable postdose PK data.

The "Pharmacodynamic" population will consist of all subjects who received at least one dose of LY3475766 and have evaluable postdose PD data.

All protocol deviations that occur during the study will be considered for their severity/impact and will be taken into consideration when subjects are assigned to analysis populations.

9. STATISTICAL METHODOLOGY

9.1 General

Data listings will be provided for all data that is databased. Summary statistics and statistical analysis will only be presented for data where detailed in this SAP. For continuous data, summary statistics will include the arithmetic mean, arithmetic standard deviation (SD), median, min, max and N; for log-normal data (e.g. the PK parameters: Area under the concentration

versus time curve [AUCs] and maximum observed drug concentration [C_{max}]) the geometric mean and geometric coefficient of variation (CV%) will also be presented. For categorical data, frequency count and percentages will be presented. Data listings will be provided for all subjects up to the point of withdrawal, with any subjects excluded from the relevant population highlighted. Summary statistics and statistical analyses will generally only be performed for subjects included in the relevant analysis population. For the calculation of summary statistics and statistical analysis, unrounded data will be used.

Mean change from baseline is the mean of all individual subjects' change from baseline values. Each individual change from baseline will be calculated by subtracting the individual subject's baseline value from the value at the timepoint. The individual subject's change from baseline values will be used to calculate the mean change from baseline using a SAS procedure such as Proc Univariate.

Data analysis will be performed using SAS® Version 9.4 or greater.

9.2 Demographics and Subject Disposition

Subject disposition will be listed. The demographic variables age, sex, race, ethnicity, country of enrolment, body weight, height and body mass index will be summarized by treatment, and listed. Screening triglyceride (TG) and low-density lipoprotein cholesterol (LDL-C) will also be summarized by treatment, and listed. All other demographic variables will be listed only.

A disposition for all enrolled subjects will also be provided.

9.3 Pharmacokinetic Assessment

9.3.1 Pharmacokinetic Analysis

The PK parameter estimates will be determined using non-compartmental procedures in validated software program (Phoenix WinNonlin Version 8.1 or later).

Plasma concentrations of LY3475766 will be used to determine the following PK parameters, when possible:

Parameter	Units	Definition
$AUC(0\text{-}t_{last})$	h*µg/mL	area under the concentration versus time curve from time zero to time t, where t is the last time point with a measurable concentration
$AUC(0\text{-}\infty)$	h*µg/mL	area under the concentration versus time curve from time zero to infinity
$\%AUC(t_{last}\text{-}\infty)$	%	percentage of $AUC(0\text{-}\infty)$ extrapolated
C_{max}	µg/mL	maximum observed drug concentration
t_{max}	h	time of maximum observed drug concentration
$t_{1/2}$	h	half-life associated with the terminal rate constant (λ_z) in non-compartmental analysis
CL	L/h	total body clearance of drug calculated after IV administration
CL/F	L/h	apparent total body clearance of drug calculated after extra-vascular administration
V_z	L	volume of distribution during the terminal phase after IV administration
V_z/F	L	apparent volume of distribution during the terminal phase after extra-vascular administration
F	%	bioavailability based on $AUC(0\text{-}\infty)$ $$F = \frac{\text{AUC [SC]} \times \text{Dose[IV]}}{\text{AUC [IV]} \times \text{Dose[SC]}} \times 100$$

Additional PK parameters may be calculated, as appropriate.

The software and version used for the final analyses will be specified in the CSR. Any exceptions or special handling of data will be clearly documented within the final study report.

Formatting of tables, figures and abbreviations will follow the Eli Lilly Global PK/PD/TS Tool: NON-COMPARTMENTAL PHARMACOKINETIC STYLE GUIDE. The version of the tool effective at the time of PK analysis will be followed.

General PK Parameter Rules

- Actual sampling times will be used in the final analyses of individual PK parameters, except for non-bolus pre-dose sampling times which will be set to zero. For non-bolus, multiple dose profiles, the pre-dose time will be set to zero unless a time deviation falls outside of the protocol blood collection time window which is considered to impact PK parameter derivation.

- C_{max} and t_{max} will be reported from observed values. If C_{max} occurs at more than one time point, t_{max} will be assigned to the first occurrence of C_{max}.

- AUC parameters will be calculated using a combination of the linear and logarithmic trapezoidal methods (linear-log trapezoidal rule). The linear trapezoidal method will be

applied up to t_{max} and then the logarithmic trapezoidal method will be used after t_{max}. The minimum requirement for the calculation of AUC will be the inclusion of at least three consecutive concentrations above the lower limit of quantification (LLOQ), with at least one of these concentrations following C_{max}.

- AUC(0-∞) values where the percentage of the total area extrapolated is more than 20% will be flagged. Any AUC(0-∞) value excluded from summary statistics will be noted in the footnote of the summary table.

- Half-life ($t_{1/2}$) will be calculated, when appropriate, based on the apparent terminal log-linear portion of the concentration-time curve. The start of the terminal elimination phase for each subject will be defined by visual inspection and generally will be the first point at which there is no systematic deviation from the log-linear decline in plasma concentrations. Half-life will only be calculated when a reliable estimate for this parameter can be obtained comprising of at least 3 data points. If $t_{1/2}$ is estimated over a time window of less than 2 half-lives, the values will be flagged in the data listings. Any $t_{1/2}$ value excluded from summary statistics will be documented in the footnote of the summary table.

- A uniform weighting scheme will be used in the regression analysis of the terminal log-linear portion of the concentration-time curve.

- The parameters based on the predicted last observed concentration will be reported.

Individual PK Parameter Rules

- Only quantifiable concentrations will be used to calculate PK parameters with the exception of special handling of certain concentrations reported below the lower limit of quantitation (BQL). Plasma concentrations reported as BQL will be set to a value of zero when all of the following conditions are met:
    - The compound is non-endogenous.
    - The samples are from the initial dose period for a subject or from a subsequent dose period following a suitable wash-out period.
    - The time points occur before the first quantifiable concentration.

- All other BQL concentrations that do not meet the above criteria will be set to missing.

- Also, where two or more consecutive concentrations are BQL towards the end of a profile, the profile will be deemed to have terminated and therefore any further quantifiable concentrations will be set to missing for the calculation of the PK parameters unless it is considered to be a true characteristic of the profile of the drug.

- For multiple-dosing data, when pre-dose concentrations are missing, the value to be substituted will be minimum observed concentration for the dosing interval.

Individual Concentration vs. Time Profiles

- Individual concentrations will be plotted utilizing actual sampling times.
- The terminal point selections will be indicated on a semi-logarithmic plot.

Average Concentration vs. Time Profiles

- The average concentration profiles will be graphed using scheduled (nominal) sampling times.
- The average concentration profiles will be graphed using arithmetic average concentrations.
- The pre-dose average concentration for single-dose data from non-endogenous compounds will be set to zero. Otherwise, only quantifiable concentrations will be used to calculate average concentrations.
- Concentrations at a sampling time exceeding the sampling time window specified in the protocol, or ± 10%, will be excluded from the average concentration profiles.
- Concentrations excluded from the mean calculation will be documented in the final study report.
- A concentration average will be plotted for a given sampling time only if 2/3 of the individual data at the time point have quantifiable measurements that are within the sampling time window specified in the protocol or ± 10%. An average concentration estimated with less than 2/3 but more than 3 data points may be displayed on the mean concentration plot if determined to be appropriate and will be documented within the final study report.

Treatment of Outliers during Pharmacokinetic Analysis

Application of this procedure to all PK analyses is not a requirement. Rather, this procedure provides justification for exclusion of data when scientifically appropriate. This procedure describes the methodology for identifying an individual value as an outlier for potential exclusion, but does not require that the value be excluded from analysis. The following methodology will not be used to exclude complete profiles from analysis.

<u>Data within an Individual Profile</u>

A value within an individual profile may be excluded from analysis if any of the following criteria are met:

- For PK profiles during single dosing of non-endogenous compounds, the concentration in a pre-dose sample is quantifiable.

- For any questionable datum that does not satisfy the above criteria, the profile will be evaluated and results reported with and without the suspected datum.

Data between Individual Profiles

1. If n<6, then the dataset is too small to conduct a reliable range test. Data will be analyzed with and without the atypical value, and both sets of results will be reported.

2. If n≥6, then an objective outlier test will be used to compare the atypical value to other values included in that calculation:

   a. Transform all values in the calculation to the logarithmic domain.

   b. Find the most extreme value from the arithmetic mean of the log transformed values and exclude that value from the dataset.

   c. Calculate the lower and upper bounds of the range defined by the arithmetic mean ±3*SD of the remaining log-transformed values.

   d. If the extreme value is within the range of arithmetic mean ±3*SD, then it is not an outlier and will be retained in the dataset.

   e. If the extreme value is outside the range of arithmetic mean ±3*SD, then it is an outlier and will be excluded from analysis.

If the remaining dataset contains another atypical datum suspected to be an outlier and n ≥ 6 following the exclusion, then repeat step 2 above. This evaluation may be repeated as many times as necessary, excluding only one suspected outlier in each iteration, until all data remaining in the dataset fall within the range of arithmetic mean ±3*SD of the log-transformed values.

Reporting of Excluded Values

Individual values excluded as outliers will be documented in the final report. Approval of the final report will connote approval of the exclusion.

9.3.2 Pharmacokinetic Statistical Methodology

PK dose proportionality will be assessed using a power model. Log-transformed C_{max}, AUC(0-t_{last}) and AUC(0-∞) estimates will be evaluated using a power model (where log-dose acts as an explanatory variable) to estimate ratios of dose-normalized geometric means and corresponding 90% confidence intervals (CIs).

The estimated ratio of dose-normalized geometric means of PK parameters between the highest and lowest doses will be used to assess dose proportionality. Between subject variability estimates will also be provided.

Example SAS code for the analysis:

```
proc mixed data=xxx;
model log_pk = log_dose / alpha=0.1 cl solution outpred=resids ddfm=kr;
estimate 'xx mg' intercept 1 log_dose yy / alpha=0.1 cl; /*Log value of xx*/
estimate 'zz mg - xx mg' log_dose pp / alpha=0.1 cl; /*Difference in log
values of zz and xx*/
ods output solutionf=est;
ods output estimates=estims;
run;
```

9.4 Pharmacodynamic Assessment

9.4.1 Pharmacodynamic Analysis

The following PD parameters will be listed and summarized by treatment and timepoint (as appropriate):

- Fasting lipid panel (TG, total cholesterol, low-density lipoprotein cholesterol [LDL-C] (direct assay from central lab), very low-density lipoprotein cholesterol [calculated], high-density lipoprotein cholesterol, non-high-density lipoprotein cholesterol [calculated], apolipoproteins [apoA-I, apoB, and apoC-III]), change from baseline (Day 1 predose) and percentage change from baseline.
- Fasting angiopoietin-like protein 3/8 complex levels, change from baseline (Day 1 predose) and percentage change from baseline.

For the fasting lipid panel (except for apoB and angiopoietin-like protein 3/8 complex levels), summary statistics will include arithmetic mean, standard deviation, geometric mean, CV%, median, minimum, maximum, Q1, Q3 (due to the distribution of the data). For apoB and angiopoietin-like protein 3/8 complex levels, standard summary statistics will be presented.

In addition, a mixed-model repeated-measure model will be used to evaluate treatment effect for the parameters change from baseline LDL-C, TG, apoB and angiopoietin-like protein 3/8. The model will include treatment, timepoint, and treatment by time point interaction as fixed effects, subject as a random effect and baseline as a covariate. An unstructured covariance structure will be used and a compound symmetric structure can be used if the model fails to converge.

Example of SAS code (for the change from baseline analysis) as follows.

```
proc mixed data=xxx;
class treatment;
model change = baseline treatment timepoint treatment*timepoint
/residual ddfm=kr;
repeated timepoint / subject=subject type=UN;
lsmeans treatment*timepoint / cl pdiff alpha=0.1;
ods output lsmeans=lsm diffs=estims;
run;
```

For the above analysis, TG and LDL-C will be log transformed prior to analysis, and back-transformed to calculate percent changes from baseline.

Absolute and change from baseline arithmetic mean time profiles of the PD parameters will be produced.

9.5 Safety and Tolerability Assessments

9.5.1 Adverse events

Where changes in severity are recorded in the Case Report Form (CRF), each separate severity of the adverse event (AE) will be reported in the listings, only the most severe will be used in the summary tables. A pre-existing condition is defined as an AE that starts before the subject has provided written informed consent and is ongoing at consent. A non-treatment emergent AE is defined as an AE which starts after informed consent but prior to dosing. A treatment-emergent AE is defined as an AE which occurs postdose or which is present prior to dosing and becomes more severe postdose.

All AEs will be listed. Treatment-emergent AEs will be summarized by treatment, severity and relationship to the study drug. The frequency (the number of AEs, the number of subjects experiencing an AE and the percentage of subjects experiencing an AE) of treatment-emergent AEs will be summarized by treatment, Medical Dictionary for Regulatory Activities (MedDRA) version 22.0 system organ class and preferred term. The summary and frequency AE tables will be presented for all causalities and those considered related to the study drug. Any serious AEs will be listed.

Discontinuations due to AEs will be listed.

9.5.2 Concomitant medication

Concomitant medication will be coded using the World Health Organization (WHO) drug dictionary (Version March 2019). Concomitant medication will be listed.

9.5.3 Clinical laboratory parameters

All clinical chemistry and hematology data will be summarized by parameter and treatment, together with changes from baseline, where baseline is defined as the scheduled assessment immediately prior to dosing, and listed. Urinalysis data will be listed. Additionally, clinical chemistry, hematology and urinalysis data outside the reference ranges will be listed.

Values for any clinical chemistry, liver panel, hematology and urinalysis values outside the reference ranges will be flagged on the individual subject data listings.

9.5.4 Vital signs

Automated office blood pressure monitoring data (AOBPM) (systolic blood pressure, diastolic blood pressure and pulse rate) will be recorded. Four records are recorded, the first is discarded and for the latter 3 records, the mean will be calculated and used for reporting and analysing.

Vital signs data and AOBPM data will be summarized by treatment and timepoint together with changes from baseline, where baseline is defined as the Day 1 predose assessment. Figures of

mean vital signs and mean changes from baseline profiles over time will be presented by treatment.

Values for individual subjects will be listed.

The relationship between time-matched systolic blood pressure, diastolic blood pressure, and heart rate from AOBPM data and the LY3475766 PK concentration will be evaluated graphically using a scatter plot. The plot will include a longitudinal linear model with independent variables, PK concentration as a continuous variable and time relative to the first dose time as a categorical variable. A compound-symmetry variance-covariance structure will be used to model the within-subject correlation. If the model fails to converge, an alternative structure will be investigated instead. The estimated regression line and associated 90% CI will be fitted on the plot and the p-value for the slope reported.

Example of SAS code as follows.

```
proc mixed data=xxx;
class time subject;
model vital = pk_conc time /residual ddfm=kr;
repeated time / subject=subject type=cs alpha=0.1;
run;
```

For the AOPBM data, a repeated-measures statistical analysis of absolute and change from baseline vital sign measurements will be produced with fixed effects for treatment, timepoint, and the interaction treatment and timepoint and baseline as a covariate (for the change from baseline analysis). An unstructured covariance structure will be used and a compound symmetric structure can be used if the model fails to converge. The primary contrasts of interest are each dose level versus placebo at each timepoint. Least squares means as well as a 90% CI of the mean difference between each dose level and placebo will be obtained.

Example of SAS code as follows.

```
proc mixed data=xxx;
class treatment;
model change = baseline treatment timepoint treatment*timepoint
/residual ddfm=kr;
repeated timepoint / subject=subject type=UN;
lsmeans treatment*timepoint / cl pdiff alpha=0.1;
ods output lsmeans=lsm diffs=estims;
run;
```

For the AOPBM data, for each subject, the maximum value within the 3 and 9 weeks after the first dose will be summarized by treatment, and listed.

In addition, the calculated maximums values will be compared between dose groups using a linear model with treatment as a fixed effect and baseline value as a covariate. The analysis will compare each dose of LY3475766 against placebo. Least-squares means as well as 90% CIs for the difference of LY3475766 compared with placebo will be reported.

Example of SAS code as follows.

```
proc mixed data=xxx;
class treatment;
model max = baseline treatment /residual ddfm=kr;
lsmeans treatment / cl pdiff alpha=0.1;
ods output lsmeans=lsm diffs=estims;
run;
```

9.5.5 Electrocardiogram (ECG)

ECGs will be performed for safety monitoring purposes only and will not be presented. Any clinically significant findings from ECGs will be reported as an AE.

9.5.6 Hepatic Monitoring

If a subject experiences elevated alanine aminotransferase (ALT) ≥3× upper limit of normal (ULN), alkaline phosphatase (ALP) ≥2× ULN, or elevated total bilirubin (TBL) ≥2× ULN, liver tests will be performed to confirm the abnormality. Additional safety data may be collected if required, as defined in the protocol. Where applicable, the following will be presented.

The subjects' liver disease history and associated person liver disease history data will be listed. Any concomitant medication that have potential for hepatotoxicity, including acetaminophen will be listed. Results from any hepatic monitoring procedures, such as a magnetic resonance elastography (MRE) scan, and biopsy assessments will be listed, if performed.

Hepatic risk factor assessment data will be listed. Liver related signs and symptoms data will be summarized by treatment, and listed. Alcohol and recreational drug use data will also be listed.

All hepatic chemistry, hematology, coagulation, and serology data will be listed. Values outside the reference ranges will be flagged on the individual subject data listings.

9.5.7 Immunogenicity Assessments

The frequency and percentage of subjects with pre-existing antidrug antibody (ADA) and with treatment-emergent ADAs (TE ADA) to LY3475766 will be tabulated and listed.

For subjects who are ADA negative at baseline, TE ADAs (treatment induced) are defined as those with a titer 2-fold (1 dilution) greater than the minimum required dilution of the assay (1:10). For subjects who are ADA positive at baseline, TE ADAs (treatment boosted) are defined as those with a 4-fold (2 dilution) increase in titer compared to baseline. The frequency and percentage of subjects with cross-reactive and neutralizing antibodies, if measured, may also be tabulated for subjects with TE ADA.

9.5.8 Injection-Site Reactions

Injection-site reaction AE data will be listed and summarized by treatment in frequency tables (if appropriate).

9.5.9 Other assessments

Body weight data will be listed and summarized by treatment and timepoint.

All other safety assessments not detailed in this section will be listed but not summarized or statistically analyzed.

10. INTERIM ANALYSES

No interim analyses are planned for this study.

11. CHANGES FROM THE PROTOCOL SPECIFIED STATISTICAL ANALYSES

There were no changes from the protocol specified statistical analyses.

12. REFERENCES

1. International Conference on Harmonization of Technical Requirements for Registration of Pharmaceuticals for Human Use, ICH Harmonized Tripartite Guideline, Statistical Principles for Clinical Trials (E9), 5 February 1998.

2. International Conference on Harmonization of Technical Requirements for Registration of Pharmaceuticals for Human Use, ICH Harmonized Tripartite Guideline, Structure and Content of Clinical Study Reports (E3), 30 November 1995.

13. DATA PRESENTATION

13.1 Derived Parameters

Individual derived parameters (e.g. PK parameters) and appropriate summary statistics will be reported to three significant figures. Observed concentration data, e.g. C_{max}, should be reported as received. Observed time data, e.g. t_{max}, should be reported as received. N and percentage values should be reported as whole numbers. Median values should be treated as an observed parameter and reported to the same number of decimal places as minimum and maximum values.

13.2 Missing Data

Missing data will not be displayed in listings.

13.3 Insufficient Data for Presentation

Some of the TFLs may not have sufficient numbers of subjects or data for presentation. If this occurs, the blank TFL shell will be presented with a message printed in the centre of the table, such as, “No serious adverse events occurred for this study.”

Leo Document ID = 6c3559eb-e54f-4c3f-9ac1-f95cc2577b79

PPD

Approval Date & Time: 19-Apr-2021 16:28:58 GMT

Signature meaning: Approved

PPD

Approval Date & Time: 20-Apr-2021 10:20:49 GMT

Signature meaning: Approved

PPD

Approval Date & Time: 20-Apr-2021 17:38:44 GMT

Signature meaning: Approved

PPD

Approval Date & Time: 21-Apr-2021 08:53:32 GMT

Signature meaning: Approved

PPD

Approval Date & Time: 23-Apr-2021 14:40:05 GMT

Signature meaning: Approved
