# Supplementary material for: Targeting the angiopoietin-like protein 3/8 complex with a monoclonal antibody in patients with mixed hyperlipidemia: a phase 1 trial
Source: Nat Med. 2025 Jul 10;31(8):2632–9. doi: 10.1038/s41591-025-03830-4 (PMC12353791; doi:10.1038/s41591-025-03830-4)
Supplement: Supplementary file 2 — Reporting Summary [file 41591_2025_3830_MOESM2_ESM.pdf]

Corresponding author(s): Daniel Gaudet
Giacomo Ruotolo

Last updated by author(s): May 21, 2025

Reporting Summary

Nature Portfolio wishes to improve the reproducibility of the work that we publish. This form provides structure for consistency and transparency in reporting. For further information on Nature Portfolio policies, see our Editorial Policies and the Editorial Policy Checklist.

Statistics

For all statistical analyses, confirm that the following items are present in the figure legend, table legend, main text, or Methods section.

n/a	Confirmed	
☐	☒	The exact sample size (n) for each experimental group/condition, given as a discrete number and unit of measurement
☒	☐	A statement on whether measurements were taken from distinct samples or whether the same sample was measured repeatedly
☐	☒	The statistical test(s) used AND whether they are one- or two-sided<br>*Only common tests should be described solely by name; describe more complex techniques in the Methods section.*
☐	☒	A description of all covariates tested
☐	☒	A description of any assumptions or corrections, such as tests of normality and adjustment for multiple comparisons
☐	☒	A full description of the statistical parameters including central tendency (e.g. means) or other basic estimates (e.g. regression coefficient) AND variation (e.g. standard deviation) or associated estimates of uncertainty (e.g. confidence intervals)
☐	☒	For null hypothesis testing, the test statistic (e.g. F, t, r) with confidence intervals, effect sizes, degrees of freedom and P value noted<br>*Give P values as exact values whenever suitable.*
☒	☐	For Bayesian analysis, information on the choice of priors and Markov chain Monte Carlo settings
☒	☐	For hierarchical and complex designs, identification of the appropriate level for tests and full reporting of outcomes
☐	☒	Estimates of effect sizes (e.g. Cohen's d, Pearson's r), indicating how they were calculated

Our web collection on statistics for biologists contains articles on many of the points above.

Software and code

Policy information about availability of computer code

Data collection	No specific software was used for data collection. The collected data were entered using the Veeva Vault Clinical Data Management suite electronic data collection system.
Data analysis	Data analysis was performed using SAS version 9.4.

For manuscripts utilizing custom algorithms or software that are central to the research but not yet described in published literature, software must be made available to editors and reviewers. We strongly encourage code deposition in a community repository (e.g. GitHub). See the Nature Portfolio guidelines for submitting code & software for further information.

Data

Policy information about availability of data

All manuscripts must include a data availability statement. This statement should provide the following information, where applicable:

- Accession codes, unique identifiers, or web links for publicly available datasets
- A description of any restrictions on data availability
- For clinical datasets or third party data, please ensure that the statement adheres to our policy

Eli Lilly and Company provides access to all individual participant data collected during the trial, after anonymization, except for pharmacokinetic or genetic data. Data are available to request 6 months after the indication studied has been approved in the US and EU and after primary publication acceptance, whichever is later. No expiration date of data requests is currently set once data are made available. In order to ensure appropriate use and analysis of data, access is provided

after a proposal has been approved by an independent review committee identified for this purpose and after receipt of a signed data sharing agreement and will be provided as soon as reasonably possible. Data and documents, including the study protocol, statistical analysis plan, clinical study report, and blank or annotated case report forms, will be provided in a secure data sharing environment. For details on submitting a request, see the instructions provided at www.vivli.org.

Research involving human participants, their data, or biological material

Policy information about studies with human participants or human data. See also policy information about sex, gender (identity/presentation), and sexual orientation and race, ethnicity and racism.

Reporting on sex and gender	Provided in manuscript, Table 1; Participants were enrolled irrespective of their sex and gender. Any data on sex and gender was collected at each clinical trial site.
Reporting on race, ethnicity, or other socially relevant groupings	Provided in manuscript, Table 1; Participants were enrolled irrespective of their race, ethnicity, or other socially relevant groupings. Any data on ace, ethnicity, or other socially relevant groupings was collected at each clinical trial site.
Population characteristics	Provided in manuscript, Table 1.
Recruitment	Recruitment occured at the medical research sites in the United States. Sites used their own database and advertisements which just mentioned demographics and compensation. There was no identifiable self-selection or other bias.
Ethics oversight	The trial was approved by Midlands IRB, Lenexa KS, USA

Note that full information on the approval of the study protocol must also be provided in the manuscript.

Field-specific reporting

Please select the one below that is the best fit for your research. If you are not sure, read the appropriate sections before making your selection.

☒ Life sciences ☐ Behavioural & social sciences ☐ Ecological, evolutionary & environmental sciences

For a reference copy of the document with all sections, see nature.com/documents/nr-reporting-summary-flat.pdf

Life sciences study design

All studies must disclose on these points even when the disclosure is negative.

Sample size	Up to 70 subjects could have been enrolled so that 48 subjects (LY3475766: 36, Placebo: 12) have sufficient evaluable data. The sample size is customary for Phase 1 studies evaluating safety and PK, and is not powered on the basis of statistical hypothesis testing. Subjects who are randomized and who are discontinued from the study (providing that discontinuation was not as a result of a safety finding) may be replaced to ensure that enough subjects complete the study.
Data exclusions	No data were excluded
Replication	Replicate measurements were not obtained for clinical or laboratory data. However there was replicate confirmation for statistical analysis. Each statistical analysis was conducted independently by two analysts: a primary analyst and a validation analyst. The validation analysts independently verified the results, and all analyses yielded matching outcomes between the two analysts.
Randomization	At study completion, 4 cohorts of 8 subjects each (6 LY3475766 : 2 placebo), and 1 cohort (Cohort 4) of 16 subjects (12 LY3475766 : 4 placebo), were enrolled and randomized to receive SC or IV LY3475766, or placebo. Cohort 6 was not conducted due to recruitment issues. The number of subjects enrolled in Cohort 4 was doubled, from that which was planned in the protocol, as a result of the COVID-19 global pandemic
Blinding	All participants, investigators, and the sponsors were masked to treatment assignment.

Reporting for specific materials, systems and methods

We require information from authors about some types of materials, experimental systems and methods used in many studies. Here, indicate whether each material, system or method listed is relevant to your study. If you are not sure if a list item applies to your research, read the appropriate section before selecting a response.

Materials & experimental systems

n/a	Involved in the study
☒	☐ Antibodies
☒	☐ Eukaryotic cell lines
☒	☐ Palaeontology and archaeology
☒	☐ Animals and other organisms
☐	☒ Clinical data
☒	☐ Dual use research of concern
☒	☐ Plants

Methods

n/a	Involved in the study
☒	☐ ChIP-seq
☒	☐ Flow cytometry
☒	☐ MRI-based neuroimaging

Clinical data

Policy information about clinical studies

All manuscripts should comply with the ICMJE guidelines for publication of clinical research and a completed CONSORT checklist must be included with all submissions.

Clinical trial registration: ClinicalTrials.gov registration: NCT04052594

Study protocol: Provided in the Supplemental Information

Data collection: 4 Sept 2019 - 31 Mar 2021

Outcomes:

Primary endpoints: Safety and adverse events
Serial assessments of AEs, vital signs, and results of 12-lead electrocardiograms, single-lead telemetry, and clinical laboratory testing (including hematology, urinalysis, clinical chemistry, and serology) were performed throughout the study. To assess immunogenicity, ADAs against LY3475766 were measured using an affinity capture elution bridging assay developed based on existing methods. Serum for ADA measurements was collected at baseline and at regular intervals throughout the study. Treatment-emergent ADAs were defined by a titer two-fold (one dilution) greater than the minimum required dilution of the assay if no ADAs were detected at baseline (treatment-induced ADA) or a four-fold (two dilution) increase in titer compared to baseline if ADAs were detected at baseline (treatment-boosted ADA).

Secondary endpoints: pharmacokinetics and pharmacodynamics
Fasting plasma samples for LY3475766 were collected at 1 and 6 hours and on days 3, 8, 15, and 29 after dosing, with LY3475766 concentrations determined using a validated liquid chromatography–tandem mass spectrometry method with a lower limit of quantification of 50 ng/mL. Pharmacokinetics were determined by noncompartmental methods (Phoenix WinNonlin version 8.1), and parameters included Cmax and time to Cmax.
For pharmacodynamic measurements, overnight fasting samples were collected on day 1 (before dosing) and days 2, 3, 4, 8, 15, 22, and 29 after dosing to evaluate changes in triglyceride, total cholesterol, LDL-C, HDL-C, non–HDL-C, ApoA-I, ApoB, and ApoC-III levels. Remnant cholesterol was calculated as total cholesterol minus LDL-C minus HDL-C. In each case, the percentage change from baseline was calculated. Because ApoA-V circulates at much lower concentrations than other apolipoproteins, immunoassays are required to measure it. However, because no neutralizing antihuman ApoA-V antibodies have been described, human ApoA-V assays are susceptible to interference with ANGPTL3/8 (the levels of which increase dramatically with LY3475766 dosing). Therefore, ApoA-V levels were not measured. Percent changes in lipoprotein subclasses assessed by nuclear magnetic resonance spectroscopy, changes in cholesterol efflux capacity, and changes in the LP-IR score were also evaluated. ANGPTL3 and ANGPTL4/8 levels were measured using dedicated assays as previously described, with the ANGPTL3 assay detecting total serum levels of ANGPTL3, including ANGPTL3 present in the ANGPTL3/8 complex. Total circulating ANGPTL3/8 levels were measured using a drug-tolerant target engagement assay based on previously described techniques.

Plants

Seed stocks: *Report on the source of all seed stocks or other plant material used. If applicable, state the seed stock centre and catalogue number. If plant specimens were collected from the field, describe the collection location, date and sampling procedures.*

Novel plant genotypes: *Describe the methods by which all novel plant genotypes were produced. This includes those generated by transgenic approaches, gene editing, chemical/radiation-based mutagenesis and hybridization. For transgenic lines, describe the transformation method, the number of independent lines analyzed and the generation upon which experiments were performed. For gene-edited lines, describe the editor used, the endogenous sequence targeted for editing, the targeting guide RNA sequence (if applicable) and how the editor was applied.*

Authentication: *Describe any authentication procedures for each seed stock used or novel genotype generated. Describe any experiments used to assess the effect of a mutation and, where applicable, how potential secondary effects (e.g. second site T-DNA insertions, mosiacism, off-target gene editing) were examined.*
